# Synthesis of 5-(2-methoxy-1-naphthyl)- and 5-[2-(methoxymethyl)-1-naphthyl]-11*H*-benzo[*b*]fluorene as 2,2'-disubstituted 1,1'-binaphthyls via benzannulated enyne–allenes

**DOI:** 10.3762/bjoc.7.58

**Published:** 2011-04-19

**Authors:** Yu-Hsuan Wang, Joshua F Bailey, Jeffrey L Petersen, Kung K Wang

**Affiliations:** 1 C. Eugene Bennett Department of Chemistry, West Virginia University, Morgantown, West Virginia 26506-6045, USA

**Keywords:** benzannulated enediynyl alcohols, benzannulated enyne–allenes, 2,2'-disubstituted 1,1'-binaphthyls, 5-(1-naphthyl)-11*H*-benzo[*b*]fluorenes, Schmittel cascade cyclizations

## Abstract

5-(2-Methoxy-1-naphthyl)- and 5-[2-(methoxymethyl)-1-naphthyl]-11*H*-benzo[*b*]fluorene were synthesized by treatment of the corresponding benzannulated enediynes with potassium *tert-*butoxide in refluxing toluene to give benzannulated enyne–allenes for the subsequent Schmittel cascade cyclization reactions. The structures of these two 5-(1-naphthyl)-11*H*-benzo[*b*]fluorenes could be regarded as 2,2'-disubstituted 1,1'-binaphthyls with the newly constructed benzofluorenyl group serving as a naphthyl moiety.

## Introduction

**Scheme 1:**
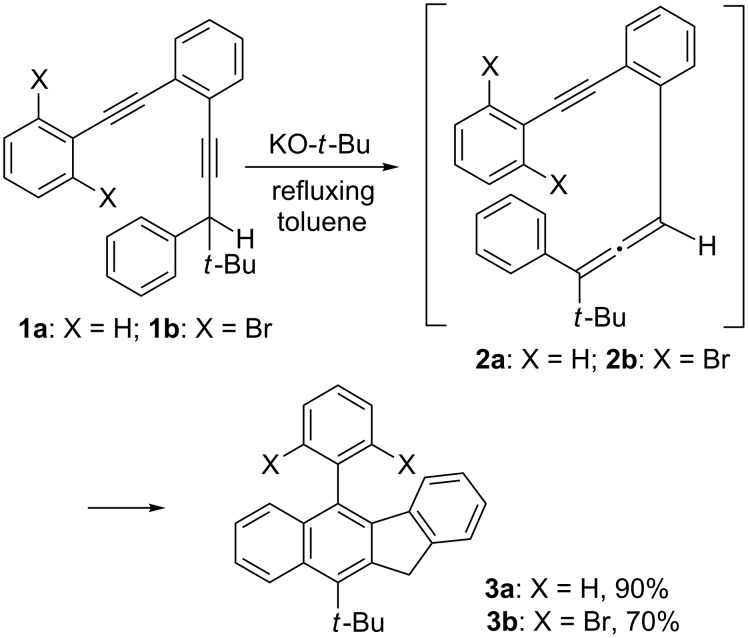
Synthesis of 5-aryl-11*H*-benzo[*b*]fluorenes via benzannulated enyne–allenes.

Benzannulated enyne–allenes bearing an aryl substituent at the alkynyl terminus are excellent precursors of 5-aryl-11*H*-benzo[*b*]fluorenes [1–5]. Several synthetic pathways to the benzannulated enyne–allene systems have been reported, including generation in situ from the corresponding benzannulated enediynes. Specifically, treatment of the benzannulated enediyne **1a** with potassium *tert*-butoxide in refluxing toluene for six hours promoted a 1,3-prototropic rearrangement to produce, in situ, the benzannulated enyne–allene **2a**, which in turn underwent a sequence of Schmittel cascade cyclization reactions to form 5-phenyl-11*H*-benzo[*b*]fluorene (**3a**) in a single operation (Scheme 1) [5]. It is interesting to note that the newly formed benzo[*b*]fluorenyl moiety in **3a** could also be regarded as a 1-arylnaphthyl derivative with three additional substituents at the 2, 3, and 4 positions.

**Scheme 2:**
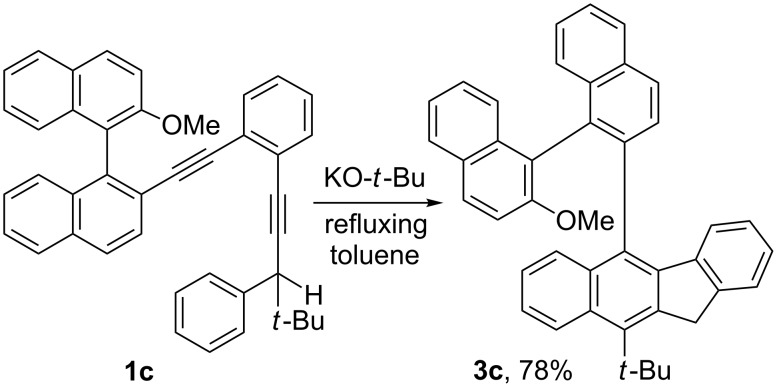
Synthesis of 1,1'-binaphthyl-substituted 11*H*-benzo[*b*]fluorene **3c**.

The reaction is not sensitive to the steric requirement of the substituent at the alkynyl terminus. The benzannulated enediyne **1b** with a sterically demanding 2,6-dibromophenyl substituent was also smoothly converted to **3b** [6]. With **1c** having a 1,1'-binaphthyl substituent, a 1:1 mixture of the *syn* and the *anti* atropisomers of **3c** was likewise obtained (Scheme 2) [7].

We now have successfully extended the cascade sequence to the synthesis of other sterically congested analogues with an ortho-methoxy or an ortho-methoxymethyl group on the phenyl substituent. In addition, by placing a 2-methoxy-1-naphthyl or a 2-(methoxymethyl)-1-naphthyl group at one of the alkynyl termini of the benzannulated enediyne system, the resulting naphthyl-substituted benzo[*b*]fluorenes could be regarded as 2,2'-disubstituted 1,1'-binaphthyls with two additional substituents at the 3 and 4 positions. The versatility of 1,1'-binaphthyl-2,2'-diol (BINOL) and BINOL derivatives as reagents in asymmetric synthesis has stimulated the development of new synthetic methods for 2,2'-disubstituted 1,1'-binaphthyls [8–16]. However, the great majority of the reported methods involved coupling of two properly substituted 1-naphthyl derivatives. Construction of a new 1-naphthyl ring as an essential step toward 1,1'-binaphthyls is rare [17–19].

## Results and Discussion

**Scheme 3:**
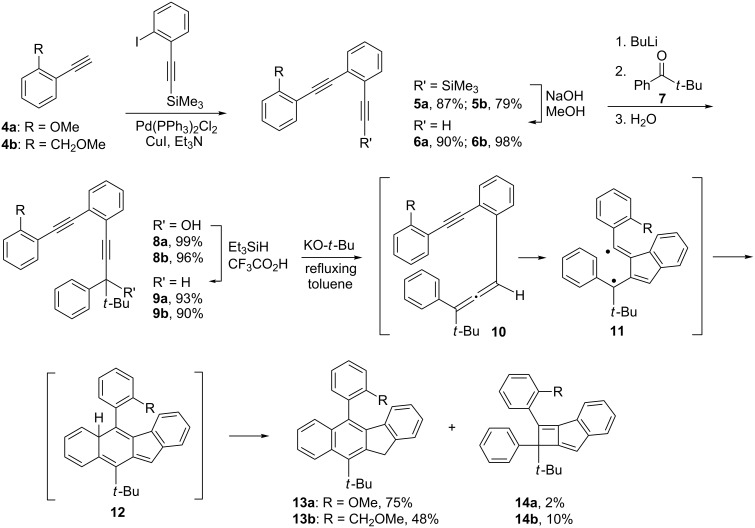
Synthesis of 5-(2-methoxyphenyl)- and 5-[2-(methoxymethyl)phenyl]-11*H*-benzo[*b*]fluorene **13a** and **13b**.

The Sonogashira reaction between 1-ethynyl-2-methoxybenzene (**4a**) and 1-iodo-2-[2-(trimethylsilyl)ethynyl]benzene produced **5a**, which was desilylated to give **6a** (Scheme 3). Condensation between **6a** and pivalophenone (**7**) then furnished the benzannulated enediynyl alcohol **8a**. Subsequent reduction with triethylsilane in the presence of trifluoroacetic acid afforded the benzannulated enediyne **9a**. Similarly, the benzannulated enediyne **9b** was synthesized from 1-ethynyl-2-(methoxymethyl)benzene (**4b**). On exposure to potassium *tert*-butoxide in refluxing toluene for five hours, **9a** was transformed to 5-(2-methoxyphenyl)-11*H*-benzo[*b*]fluorene **13a** along with a small amount (ca. 2%) of **14a** in a single operation. Presumably, the cascade sequence involved an initial 1,3-protropic rearrangement to form the corresponding benzannulated enyne–allene **10a**. A Schmittel cyclization reaction [1–4] generates biradical **11a,** which then undergoes an intramolecular radical–radical coupling to afford **12a**. This is followed by a second prototropic rearrangement to restore the aromaticity to furnish **13a** as proposed previously [5]. An intramolecular [2 + 2] cycloaddition reaction of **10a** or a direct radical–radical coupling of **11a** could account for the formation of **14a** [5]. From **9b**, 5-[2-(methoxymethyl)phenyl]-11*H*-benzo[*b*]fluorene **13b** and the [2 + 2] cycloaddition adduct **14b** were produced in a 5:1 ratio. The presence of the carbon–carbon double bonds in **14b** allows easy removal of **14b** by treatment of the resulting mixture with BH_3_·THF followed by silica gel column chromatography. The presence of a benzofluorenyl moiety and a methoxy or a methoxymethyl group in **13a** and **13b** could allow these compounds to serve as hetero-bidentate ligands for complex formation with transition metals [20].

**Scheme 4:**
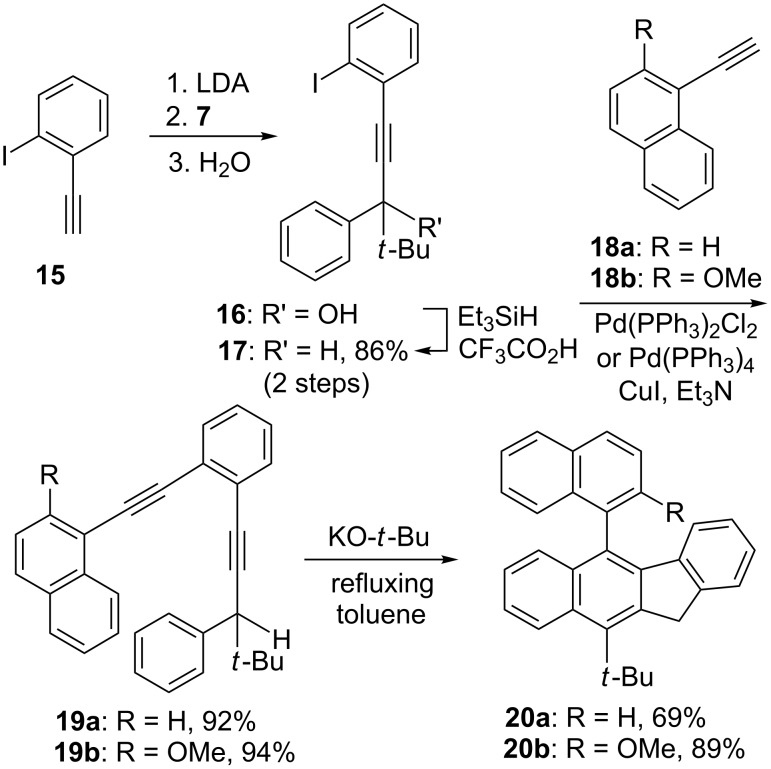
Synthesis of 5-(1-naphthyl)- and 5-(2-methoxy-1-naphthyl)-11*H*-benzo[*b*]fluorene **20a** and **20b**.

**Scheme 5:**
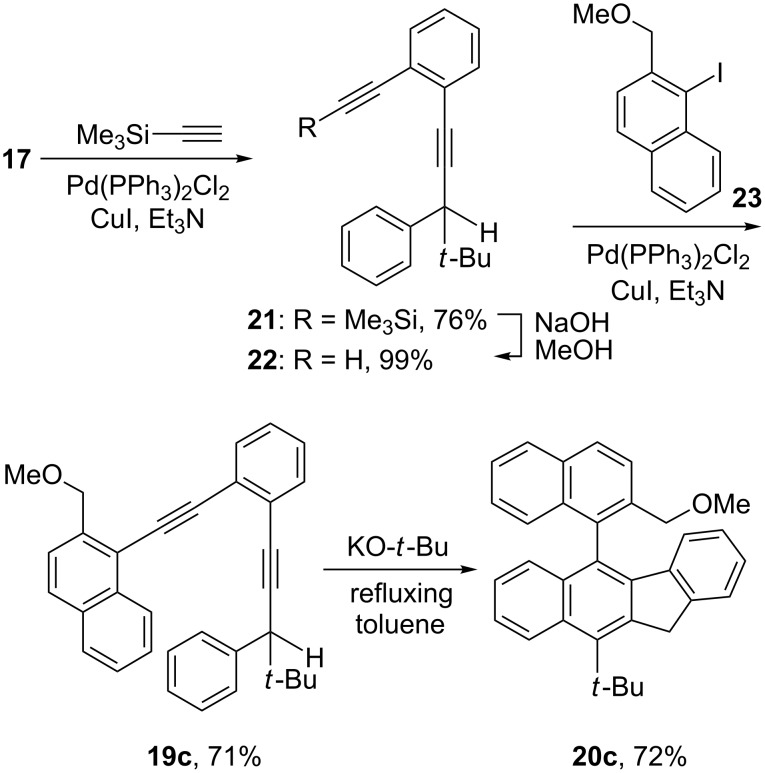
Synthesis of 5-[2-(methoxymethyl)-1-naphthyl]-11*H*-benzo[*b*]fluorene **20c**.

We also investigated the possibility of using the benzannulated enediynes bearing a 1-naphthyl, a 2-methoxy-1-naphthyl, or a 2-(methoxymethyl)-1-naphthyl substituent at one of the alkynyl termini for the cascade cyclization reaction. Using a slightly different synthetic sequence from that shown in Scheme 3 but reminiscent of a sequence developed for the synthesis of 4,5-diheteroarylphenanthrenes [21], the benzannulated enediynes **19a** and **19b** were obtained (Scheme 4). It was gratifying to observe that on treatment with potassium *tert-*butoxide, **19a** and **19b** were smoothly converted to **20a** and **20b**, respectively. Similarly **20c** was obtained via the synthetic sequence outlined in Scheme 5. It is worth noting that the synthetic sequences outlined in Scheme 4 and Scheme 5 represent new routes to 2,2'-disubstituted 1,1'-binaphthyls with the newly constructed benzofluorenyl serving as one of the naphthyl groups.

The ^1^H NMR spectrum of **13a** in C_6_D_6_ recorded on a 600 MHz NMR spectrometer exhibited an AB system with signals at δ 4.21 (*J* = 21.0 Hz) and 4.13 (*J* = 21.0 Hz), attributable to the methylene hydrogens on the five-membered ring. AB systems from the methylene hydrogens were also observed in other similar 11*H*-benzo[*b*]fluorenyl structures [7,22–24]. The AB pattern remained unchanged at 70 °C, suggesting a relatively slow rate of rotation, on the NMR time scale, around the carbon–carbon single bond connecting the 2-methoxyphenyl substituent to the C5 of the benzofluorenyl moiety. The rotational barrier was calculated to be at least 16.7 kcal/mol at 70 °C on the basis of the lack of coalescence of signals at this temperature. This lowest possible rotational barrier is significantly higher than that of 1-phenylnaphthalene, which was calculated by MM2' to be 12.4 kcal/mol [25–26]. Similarly, the ^1^H NMR spectrum of **13b** taken in CDCl_3_ showed a clear AB system at δ 4.07 (*J* = 13.8 Hz) and 4.04 (*J* = 13.8 Hz), attributable to the methylene hydrogens on the carbon attached to the methoxy group. The signals of the methylene hydrogens on the five-membered ring could barely be discerned as an AB system with the two inner signals overlapped at δ 4.51 and two small outer signals at δ 4.55 and 4.47.

The rotational barrier of the parent 1,1'-binaphthyl in *N*,*N*-dimethylformamide was determined to be 23.5 kcal/mol at 50 °C, corresponding to a half-life of 14.5 minutes for racemization [27–28]. Because the structure of **20a** could be regarded as a 2,3,4-trisubstituted 1,1'-binaphthyl, the rate of rotation can be expected to be even slower. Again, in C_6_D_6_ recorded on a 600 MHz NMR spectrometer, the signals of the methylene hydrogens on the five-membered ring could be discerned as an AB system at δ 4.27 (*J* = 21 Hz) and 4.25 (*J* = 21 Hz).

The rotational barrier of BINOL as a member of the 2,2'-disubstituted 1,1'-binaphthyls was determined to be 37.2 kcal/mol at 195 °C in naphthalene, corresponding to a half-life of 4.5 hours for racemization [28]. The high stability of the configuration even at such an elevated temperature allows BINOL to be used in a variety of synthetic applications. The configurational stability of **20b** and **20c**, which could be regarded as 2,2'-disubstituted 1,1'-binaphthyls with two additional substituents at the 3 and 4 positions, could also be expected to be high. AB patterns were observed for the methylene hydrogens on the five-membered ring of **20b** and on the carbon bearing the methoxy group of **20c**.

**Scheme 6:**
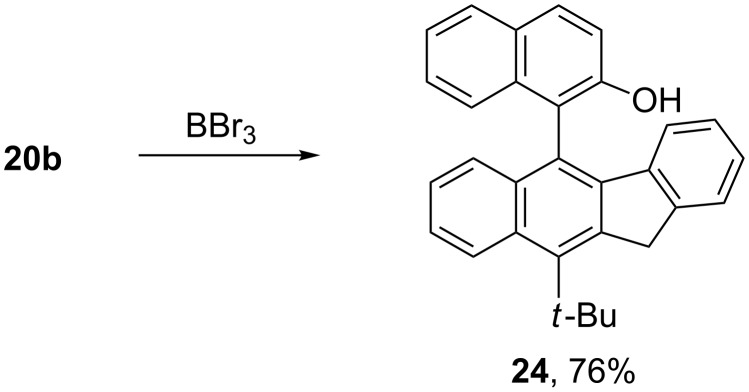
Demethylation of **22b** to form 5-(2-hydroxy-1-naphthyl)-11*H*-benzo[*b*]fluorene **24**.

Treatment of **20b** with boron tribromide converted the methoxy group to the hydroxy group, providing a handle for resolution of **24** with (1*S*)-(−)-camphanoyl chloride (Scheme 6) [29]. It was possible to achieve partial separation of a small fraction of the two diastereomeric (1*S*)-camphanates in a 5:1 ratio by silica gel column chromatography.

## Conclusion

In conclusion, the use of benzannulated enediynes as precursors to 2,2'-disubstituted 1,1'-binaphthyls represents a new synthetic approach to these sterically hindered molecules. The assembly of the enediynyl precursors from three separate aromatic fragments allows the possibility of placing a variety of functional groups at various positions of the 1,1'-binaphthyl system. Transformation of the methoxy group in **20b** to a hydroxy group provides a handle for resolution with optically active reagents.

## Experimental

### General information

All organometallic reactions were conducted in oven-dried (120 °C) glassware under a nitrogen atmosphere. Diethyl ether and tetrahydrofuran (THF) were distilled from benzophenone ketyl prior to use. 1-Ethynyl-2-methoxybenzene (**4a**), pivalophenone (**7**), *n*-butyllithium (1.6 M) in hexanes, 1-bromo-2-[2-(trimethylsilyl)ethynyl]benzene, triethylsilane, trifluoroacetic acid, potassium *tert-*butoxide (1.0 M) in 2-methyl-2-propanol, lithium diisopropylamide (LDA, 2.0 M) in heptane/THF/ethylbenzene, Pd(PPh_3_)_2_Cl_2_, Pd(PPh_3_)_4_, copper(I) iodide, triethylamine, 1.0 M BH_3_·THF solution in THF, 1-ethynylnaphthalene (**18a**), 2-methoxy-1-naphthaldehyde, 1-bromo-2-(methoxymethyl)naphthalene, (trimethylsilyl)acetylene, boron tribromide, and (1*S*)-(−)-camphanoyl chloride were purchased from chemical suppliers and were used as received. 1-Iodo-2-[2-(trimethylsilyl)ethynyl]benzene was prepared by treatment of 1-bromo-2-[2-(trimethylsilyl)ethynyl]benzene in THF with *n*-butyllithium at −78 °C followed by treatment with iodine [7]. 1-Ethynyl-2-iodo-benzene (**15**) [30] was prepared in quantitative yield by desilylation of 1-iodo-2-[2-(trimethylsilyl)ethynyl]benzene with sodium hydroxide in methanol. 1**-**Ethynyl**-**2**-**(methoxymethyl)benzene (**4b**) [31] and dimethyl (1-diazo-2-oxopropyl)phosphonate [32] were prepared according to the reported procedures.

**1-Iodo-2-(4,4-dimethyl-3-phenyl-1-pentynyl)benzene (17).** To 1.032 g (4.528 mmol) of **15** in 10 mL THF under a nitrogen atmosphere at 0 °C, was added 3.77 mL of a 2.0 M solution of LDA (7.55 mmol) in THF. After stirring for 30 min, a solution of 0.616 g of **7** (3.774 mmol) in 10 mL of THF was introduced via cannula, and the reaction mixture allowed to warm to room temperature. After an additional 3 h, 20 mL of water was introduced, and the reaction mixture extracted with diethyl ether. The combined organic extracts were washed successively with brine and water, dried over sodium sulfate, and concentrated. The residue was purified by flash chromatography (silica gel/10% diethyl ether in hexanes) to afford 1.476 g of crude **16** as a light yellow liquid. Crude **16** without any further purification was treated with 0.810 g of triethylsilane (6.983 mmol) and 2.1 g of trifluoroacetic acid (18.4 mmol) to afford 1.382 g (3.699 mmol, 86% for 2 steps) of **17** as a colorless liquid: IR 2966, 1463 cm^−1^; ^1^H NMR (CDCl_3_, 270 MHz) δ 7.83 (1H, dd, *J* = 7.9, 1.2 Hz), 7.47–7.41 (3H, m), 7.36–7.23 (4H, m), 6.96 (1H, td, *J* = 7.7, 1.7 Hz), 3.69 (1H, s), 1.09 (9H, s); ^13^C NMR (CDCl_3_, 67.9 MHz) δ 138.9, 138.6, 132.9, 130.5, 129.9, 128.8, 127.6, 126.7, 100.5, 95.5, 85.5, 50.6, 35.7, 27.9.

**Benzannulated enediyne 19b.** To a mixture of 0.307 g of **17** (0.822 mmol), Pd(PPh_3_)_4_ (0.040 g, 0.035 mmol), and copper(I) iodide (0.015 g, 0.080 mmol) in 10 mL of toluene, was added via cannula a solution of 0.150 g of **18b** (0.824 mmol) in 5 mL of triethylamine. After stirring at 120 °C for 12 h, 15 mL of a saturated ammonium chloride solution and 15 mL of diethyl ether were added. The organic layer was separated and the aqueous layer back extracted with diethyl ether. The combined organic layers were washed successively with brine and water, dried over sodium sulfate, and concentrated. Purification of the residue by flash column chromatography (silica gel/30% methylene chloride in hexanes) afforded 0.331 g of **19b** (0.773 mmol, 94%) as a colorless liquid: IR 2207, 1271, 1078 cm^−1^; ^1^H NMR (CDCl_3_, 270 MHz) δ 8.43–8.39 (1H, m), 7.85 (1H, d, *J* = 9.2 Hz), 7.82–7.77 (1H, m), 7.70–7.65 (1H, m), 7.56–7.52 (1H, m), 7.43–7.25 (7H, m), 7.10–7.00 (3H, m), 4.00 (3H, s), 3.66 (1H, s), 0.95 (9H, s); ^13^C NMR (CDCl_3_, 67.9 MHz) δ 158.9, 139.0, 134.5, 132.3, 130.2, 129.7, 128.4, 127.9, 127.8, 127.3, 127.2, 126.4, 126.3, 126.0, 125.7, 124.1, 112.6, 106.5, 97.8, 95.5, 87.3, 82.6, 56.6, 50.5, 35.5, 27.7; HRMS *m*/*z* [M + H]^+^ calcd for C_32_H_29_O, 429.2218; found, 429.2217.

**Benzannulated enediyne 19c.** To a mixture of 0.242 g of **23** (0.812 mmol), Pd(PPh_3_)_2_Cl_2_ (0.030 g, 0.043 mmol), and copper(I) iodide (0.015 g, 0.080 mmol) in 6 mL of triethylamine, was added via cannula a solution of 0.265 g of **22** (0.974 mmol) in 2 mL of triethylamine. After stirring at 60 °C for 12 h, 15 mL of a saturated ammonium chloride solution and 15 mL of diethyl ether were added. The organic layer was separated and the aqueous layer back extracted with diethyl ether. The combined organic layers were washed successively with brine and water, dried over sodium sulfate, and concentrated. Purification of the residue by flash column chromatography (silica gel/5% methylene chloride in hexanes) afforded 0.255 g of **19c** (0.577 mmol, 71%) as a colorless liquid: ^1^H NMR (CDCl_3_, 270 MHz) δ 8.51 (1H, d, *J* = 7.7 Hz), 7.88–7.83 (2H, m), 7.67–7.57 (3H, m), 7.53–7.42 (2H, m), 7.38–7.32 (4H, m), 7.12–7.05 (3H, m), 4.92 (1H, d, *J* = 13.1 Hz), 4.83 (1H, d, *J* = 12.9 Hz), 3.69 (2H, s), 3.41 (3H, s), 0.97 (9H, s); ^13^C NMR (CDCl_3_, 67.9 MHz) δ 139.1, 138.9, 133.2, 132.5, 132.1, 129.6, 128.7, 128.1, 128.0, 127.4, 126.8, 126.6, 126.3, 126.2, 125.5, 125.0, 119.0, 98.3, 96.0, 88.4, 82.7, 72.8, 58.3, 50.5, 35.5, 27.7.

**5-(2-Methoxy-1-naphthyl)-10-(1,1-dimethylethyl)-11*H*-benzo[*b*]fluorene (20b).** To 0.295 g of **19b** (0.689 mmol) in 10 mL of anhydrous toluene under a nitrogen atmosphere, was added 0.77 mL of a 1.0 M solution of potassium *tert-*butoxide (0.77 mmol) in 2-methyl-2-propanol. The reaction mixture was then heated under reflux for 6 h. After the reaction mixture was allowed to cool to room temperature, 10 mL of water and 40 mL of methylene chloride were introduced. The organic layer was separated, dried over sodium sulfate and concentrated. The residue was purified by flash column chromatography (silica gel/5% methylene chloride in hexanes) to provide 0.263 g of **20b** (0.614 mmol, 89%) as a light yellow liquid: IR 1267, 1250, 766 cm^−1^; ^1^H NMR (CDCl_3_, 600 MHz) δ 8.66 (1H, d, *J* = 9.0 Hz), 8.11 (1H, d, *J* = 9.6 Hz), 7.92 (1H, d, *J* = 8.4 Hz), 7.53 (1H, d, *J* = 9.0 Hz), 7.44 (1H, d, *J* = 7.2 Hz), 7.40 (1H, ddd, *J* = 8.4, 6.6, 1.8 Hz), 7.35 (1H, d, *J* = 9.0 Hz), 7.31 (1H, td, *J* = 6.6, 1.2 Hz), 7.18 (1H, t, *J* = 7.8 Hz), 7.13–7.05 (3H, m), 6.77 (1H, t, *J* = 7.8 Hz), 6.08 (1H, d, *J* = 7.8 Hz), 4.55 (2H, s), 3.68 (3H, s), 1.97 (9H, s); ^1^H NMR (C_6_D_6_, 600 MHz) δ 8.67 (1H, d, *J* = 9.0 Hz), 7.90 (1H, d, *J* = 9.6 Hz), 7.79 (1H, d, *J* = 8.4 Hz), 7.70 (1H, d, *J* = 8.4 Hz), 7.42 (1H, d, *J* = 8.4 Hz), 7.29 (1H, t, *J* = 7.8 Hz), 7.23 (1H, d, *J* = 7.2 Hz), 7.17 (1H, d, *J* = 6.6 Hz), 7.12 (1H, t, *J* = 7.5 Hz), 7.06 (1H, t, *J* = 7.5 Hz), 6.98 (1H, t, *J* = 7.2 Hz), 6.89 (1H, t, *J* = 7.8 Hz), 6.72 (1H, t, *J* = 7.5 Hz), 6.53 (1H, d, *J* = 7.8 Hz), 4.25 (1H, d, *J* = 21.6 Hz), 4.19 (1H, d, *J* = 21.6 Hz), 3.13 (3H, s), 1.77 (9H, s); ^13^C NMR (CDCl_3_, 150 MHz) δ 154.8, 144.2, 141.0, 140.3, 139.3, 137.9, 134.7, 133.9, 131.8, 129.8, 129.5, 128.1, 127.8, 127.2, 127.0, 126.7, 126.6, 126.4, 125.2, 124.1, 123.9, 123.7, 123.3, 122.8, 122.2, 114.4, 56.9, 40.3, 38.9, 34.5; MS *m*/*z* 428 (M^+^), 413, 400, 371; HRMS *m*/*z* calcd for C_32_H_28_O, 428.2140; found, 428.2126.

Recrystallization from a mixture of isopropyl alcohol and methylene chloride produced a crystal for X-ray structure analysis. Although the weakly diffracting crystal limited the amount of observed data, the analysis of these data supports the structural assignment of **20b**.

**5-[2-(Methoxymethyl)-1-naphthyl]-10-(1,1-dimethylethyl)-11*H*-benzo[*b*]fluorene (20c).** The same procedure as described for **20b** was repeated except that 0.142 g of **19c** (0.321 mmol) was treated with 0.48 mL of a 1.0 M solution of potassium *tert-*butoxide (0.48 mmol) in 2-methyl-2-propanol to afford 0.102 g of **20c** (0.231 mmol, 72%) as a light yellow liquid: IR 2943, 1273, 1248, 774 cm^−1^; ^1^H NMR (CDCl_3_, 270 MHz) δ 8.67 (1H, d, *J* = 8.9 Hz), 8.12 (1H, d, *J* = 8.7 Hz), 7.97 (1H, d, *J* = 8.2 Hz), 7.91 (1H, d, *J* = 8.6 Hz), 7.47–7.39 (3H, m), 7.28–7.08 (5H, m), 6.75 (1H, t, *J* = 7.7 Hz), 5.89 (1H, d, *J* = 7.9 Hz), 4.56 (2H, s), 4.16 (1H, d, *J* = 13.4 Hz), 4.10 (1H, d, *J* = 13.3 Hz), 3.09 (3H, s), 1.98 (9H, s); ^13^C NMR (CDCl_3_, 67.9 MHz) δ 144.1, 141.5, 139.7, 139.2, 137.7, 135.3, 134.3, 134.0, 133.2, 132.7, 131.6, 128.4, 128.1, 127.9, 126.95, 126.91, 126.6, 126.4, 126.0, 125.9, 124.9, 124.5, 123.8, 123.6, 122.9, 71.9, 58.4, 40.3, 39.0, 34.5; MS *m*/*z* 442 (M^+^), 427, 395; HRMS *m*/*z* calcd for C_33_H_30_O, 442.2297; found, 442.2283.

## Supporting Information

File 1 Experimental procedures, spectroscopic data, and ^1^H and/or ^13^C NMR spectra of **5a**,**b**, **6a**,**b**, **8a**,**b**, **9a**,**b**, **13a**,**b**, **17**, **18b**, **19a**–**c**, **20a**–**c**, **21**–**24**, and the (1*S*)-camphanates of **24**.
