# Supplementary material for: Synthesis of 5-(2-methoxy-1-naphthyl)- and 5-[2-(methoxymethyl)-1-naphthyl]-11H-benzo[b]fluorene as 2,2'-disubstituted 1,1'-binaphthyls via benzannulated enyne–allenes
Source: Beilstein J Org Chem. 2011 Apr 19;7:496–502. doi: 10.3762/bjoc.7.58 (PMC3107508; doi:10.3762/bjoc.7.58)
Supplement: File 1 — Experimental procedures, spectroscopic data, and 1H and/or 13C NMR spectra of 5a,b, 6a,b, 8a,b, 9a,b, 13a,b, 17, 18b, 19a–c, 20a–c, 21–24, and the (1S)-camphanates of 24. [file Beilstein_J_Org_Chem-07-496-s001.pdf]

Supporting Information

for

Synthesis of 5-(2-methoxy-1-naphthyl)- and 5-[2-(methoxymethyl)-1-naphthyl]-11*H*-benzo[*b*]fluorene as 2,2'-disubstituted 1,1'-binaphthyls via benzannulated enyne–allenes

Yu-Hsuan Wang, Joshua F. Bailey, Jeffrey L. Petersen and Kung K. Wang*

Address: C. Eugene Bennett Department of Chemistry, West Virginia University, Morgantown, West Virginia 26506-6045, USA

Email: Kung K. Wang* - kung.wang@mail.wvu.edu

*Corresponding author

Dedicated to Professor Anthony Winston on the occasion of his 85th birthday.

Experimental procedures, spectroscopic data, and ^{1}H and/or ^{13}C NMR spectra of **5a**,**b**, **6a**,**b**, **8a**,**b**, **9a**,**b**, **13a**,**b**, **17**, **18b**, **19a**–**c**, **20a**–**c**, **21**–**24**, and the (1*S*)-camphanates of **24**.

Table of Contents

Page	
S5	Experimental Procedure for Trimethyl[[2-[(2-methoxyphenyl)ethynyl]phenyl]ethynyl]silane (**5a**).
S5	Experimental Procedure for Benzannulated Enediyne **6a**.
S6	Experimental Procedure for α-[[2-[(2-Methoxyphenyl)ethynyl]phenyl]ethynyl]-α-(1,1-dimethylethyl)benzenemethanol (**8a**).
S6	Experimental Procedure for Benzannulated Enediyne **9a**.
S7	Experimental Procedure for 5-(2-Methoxyphenyl)-10-(1,1-dimethylethyl)-11*H*-benzo[*b*]fluorene (**13a**).
S8	Experimental Procedure for Trimethyl[[2-[[2-(methoxymethyl)phenyl] ethynyl]phenyl]ethynyl]silane (**5b**).
S8	Experimental Procedure for Benzannulated Enediyne **6b**.
S8	Experimental Procedure for α-[[2-[[2-(Methoxymethyl)phenyl]ethynyl] phenyl]ethynyl]-α-(1,1-dimethylethyl)benzenemethanol (**8b**).
S9	Experimental Procedure for Benzannulated Enediyne **9b**.
S9	Experimental Procedure for 5-[2-(Methoxymethyl)phenyl]-10-(1,1-dimethylethyl)-11*H*-benzo[*b*]fluorene (**13b**).
S10	Experimental Procedure for Benzannulated Enediyne **19a**.
S11	Experimental Procedure for 10-(1,1-Dimethylethyl)-5-(1-naphthyl)-11*H*-benzo[*b*]fluorene (**20a**).
S12	Experimental Procedure for 1-Ethynyl-2-methoxynaphthalene (**18b**).

S12 Experimental Procedure for Benzannulated Enediyne **21**.

S13 Experimental Procedure for Benzannulated Enediyne **22**.

S13 Experimental Procedure for 1-Iodo-2-(methoxymethyl)naphthalene (**23**).

S13 Experimental Procedure for 1,1′-Binaphthyl **24**.

S14 Experimental Procedure for (1*S*)-Camphanates of **24**.

S16−S17 ^{1}H and ^{13}C NMR Spectra of Trimethyl[[2-[(2-methoxyphenyl)ethynyl]phenyl]ethynyl]silane (**5a**).

S18−S19 ^{1}H and ^{13}C NMR Spectra of Benzannulated Enediyne **6a**.

S20−S21 ^{1}H and ^{13}C NMR Spectra of α-[[2-[(2-Methoxyphenyl)ethynyl]phenyl]ethynyl]-α-(1,1-dimethylethyl)benzenemethanol (**8a**).

S22−S23 ^{1}H and ^{13}C NMR Spectra of Benzannulated Enediyne **9a**.

S24−S25 ^{1}H and ^{13}C NMR Spectra of 5-(2-Methoxyphenyl)-10-(1,1-dimethylethyl)-11*H*-benzo[*b*]fluorene (**13a**).

S26−S27 ^{1}H and ^{13}C NMR Spectra of Trimethyl[[2-[[2-(methoxymethyl)phenyl] ethynyl]phenyl]ethynyl]silane (**5b**).

S28−S29 ^{1}H and ^{13}C NMR Spectra of Benzannulated Enediyne **6b**.

S30−S31 ^{1}H and ^{13}C NMR Spectra of α-[[2-[[2-(Methoxymethyl)phenyl]ethynyl] phenyl]ethynyl]-α-(1,1-dimethylethyl)benzenemethanol (**8b**).

S32−S33 ^{1}H and ^{13}C NMR Spectra of Benzannulated Enediyne **9b**.

S34−S35 ^{1}H and ^{13}C NMR Spectra of 5-[2-(Methoxymethyl)phenyl]-10-(1,1-dimethylethyl)-11*H*-benzo[*b*]fluorene (**13b**).

S36−S37 ^{1}H and ^{13}C NMR Spectra of 1-Iodo-2-(4,4-dimethyl-3-phenyl-1-pentynyl)benzene (**17**).

S38−S39 ^{1}H and ^{13}C NMR Spectra of Benzannulated Enediyne **19a**.

S40−S42 ^{1}H and ^{13}C NMR Spectra of 10-(1,1-Dimethylethyl)-5-(1-naphthyl)-11*H*-benzo[*b*]fluorene (**20a**).

S43−S44 ^{1}H and ^{13}C NMR Spectra of 1-Ethynyl-2-methoxynaphthalene (**18b**).

S45−S46 ^{1}H and ^{13}C NMR Spectra of Benzannulated Enediyne **19b**.

S47−S49 ^{1}H and ^{13}C NMR Spectra of 5-(2-Methoxy-1-naphthyl)-10-(1,1-dimethylethyl)-11*H*-benzo[*b*]fluorene (**20b**).

S50−S51 ^{1}H and ^{13}C NMR Spectra of Benzannulated Enediyne **21**.

S52−S53 ^{1}H and ^{13}C NMR Spectra of Benzannulated Enediyne **22**.

S54−S55 ^{1}H and ^{13}C NMR Spectra of 1-Iodo-2-(methoxymethyl)naphthalene (**23**).

S56−S57 ^{1}H and ^{13}C NMR Spectra of Benzannulated Enediyne **19c**.

S58−S59 ^{1}H and ^{13}C NMR Spectra of 5-[2-(Methoxymethyl)-1-naphthyl]-10-(1,1-dimethylethyl)-11*H*-benzo[*b*]fluorene (**20c**).

S60−S61 ^{1}H and ^{13}C NMR Spectra of 1,1′-Binaphthyl **24**.

S62 ^{1}H Spectrum of (1*S*)-Camphanates of **24**.

Trimethyl[[2-[(2-methoxyphenyl)ethynyl]phenyl]ethynyl]silane (5a). To a mixture of 0.318 g of 1-iodo-2-[2-(trimethylsilyl)ethynyl]benzene (1.059 mmol), $Pd(PPh_3)_2Cl_2$ (0.027 g, 0.039 mmol), and copper(I) iodide (0.008 g, 0.04 mmol) in 15 mL of triethylamine, was added via cannula a solution of 0.103 g of **4a** (0.780 mmol) in 5 mL of triethylamine. After stirring at 70 °C for 5 h, 20 mL of a saturated ammonium chloride solution and 20 mL of diethyl ether were added. The organic layer was separated and the aqueous layer back extracted with diethyl ether. The combined organic layers were washed successively with brine and water, dried over sodium sulfate, and concentrated. Purification of the residue by flash column chromatography (silica gel/30% methylene chloride in hexanes) afforded 0.206 g of **5a** (0.677 mmol, 87%) as a colorless liquid: ^{1}H ($CDCl_3$, 270 MHz) δ 7.59−7.47 (3 H, m), 7.35−7.20 (3 H, m), 6.94 (1 H, t, J = 7.7 Hz), 6.91 (1 H, d, J = 7.7 Hz), 3.91 (3 H, s), 0.26 (9 H, s) ; ^{13}C ($CDCl_3$, 67.9 MHz) δ 159.9, 133.9, 132.2, 131.9, 129.9, 128.1, 127.6, 126.4, 125.4, 120.4, 112.5, 110.7, 103.6, 98.4, 92.0, 89.9, 55.8, −0.1.

Benzannulated Enediyne 6a. To 0.201 g (0.660 mmol) of **5a** in 10 mL of diethyl ether were added 4 mL of a 10% sodium hydroxide solution and 10 mL of methanol. After stirring at room temperature for 30 min, 20 mL of water and 20 mL of diethyl ether were added. The organic layer was separated and the aqueous layer back extracted with diethyl ether. The combined organic layers were washed successively with brine and water, dried over sodium sulfate, and concentrated. Purification of the residue by flash column chromatography (silica gel/30% methylene chloride in hexanes) afforded 0.137 g of **6a** (0.591 mmol, 90%) as a yellow liquid: ^{1}H ($CDCl_3$, 270 MHz) δ 7.60−7.51 (3 H, m), 7.36−7.23 (3 H, m), 6.95 (1 H, t, J = 7.6 Hz), 6.90 (1 H, d, J = 8.2 Hz), 3.91 (3 H, s), 3.37

(1 H, s); ^{13}C ($CDCl_3$, 67.9 MHz) δ 160.0, 133.8, 132.5, 131.8, 130.0, 128.4, 127.7, 126.6, 124.4, 120.4, 112.3, 110.7, 91.8, 90.0, 82.3, 81.0, 55.8.

α-[[2-[(2-Methoxyphenyl)ethynyl]phenyl]ethynyl]-α-(1,1-dimethylethyl)benzenemethanol (8a). To 0.065 g (0.28 mmol) of **6a** in 5 mL of anhydrous diethyl ether under a nitrogen atmosphere at 0 °C, was added 0.18 mL of a 1.6 M solution of *n*-butyllithium (0.28 mmol) in hexanes. After stirring for 30 min, a solution of 0.064 g of **7** (0.39 mmol) in 10 mL of THF was introduced via cannula, and the reaction mixture allowed to warm to room temperature. After an additional 3 h, 15 mL of water was introduced and the reaction mixture extracted with diethyl ether. The combined organic extracts were washed successively with brine and water, dried over sodium sulfate, and concentrated. The residue was purified by flash column chromatography (silica gel/15% diethyl ether in hexanes) to provide 0.109 g (0.276 mmol, 99%) of **8a** as a light yellow liquid: ^{1}H ($CDCl_3$, 270 MHz) δ 7.78−7.71 (2 H, m), 7.61−7.57 (1 H, m), 7.53−7.49 (1 H, m), 7.41 (1 H, dd, J = 7.9, 1.7 Hz), 7.35−7.20 (6 H, m), 6.94−6.87 (2 H, m), 3.84 (3 H, s), 2.67 (1 H, s), 1.07 (9 H, s); ^{13}C ($CDCl_3$, 67.9 MHz) δ 159.9, 142.1, 133.7, 132.2, 131.9, 129.9, 127.9, 127.8, 127.2, 126.9, 126.1, 125.1, 120.5, 112.4, 110.9, 96.2, 92.1, 89.5, 84.6, 79.4, 55.9, 39.7, 25.5.

Benzannulated Enediyne 9a. To a mixture of **8a** (0.109 g, 0.276 mmol) and triethylsilane (0.048 g, 0.41 mmol) in 10 mL of methylene chloride, was added trifluoroacetic acid (0.126 g, 1.11 mmol). After stirring at room temperature for 1 h, sodium carbonate (0.11 g, 4.6 mmol) was added, followed by 10 mL of water and 10 mL of diethyl ether. The organic layer was separated, dried over sodium sulfate and concentrated. Purification of the residue by flash column chromatography (silica gel/25%

methylene chloride in hexanes) provided 0.097 g (0.256 mmol, 93%) of **9a** as a light yellow liquid: ^{1}H ($CDCl_3$, 270 MHz) δ 7.61−7.55 (1 H, m), 7.48−7.16 (10 H, m), 6.92−6.85 (2 H, m), 3.87 (3 H, s), 3.69 (1 H, s), 1.04 (9 H, s); ^{13}C ($CDCl_3$, 67.9 MHz) δ 160.0, 139.2, 133.7, 132.2, 132.0, 129.8, 129.7, 127.7, 127.5, 127.2, 126.5, 126.3, 125.9, 120.3, 112.5, 110.5, 95.5, 92.4, 89.1, 82.6, 55.7, 50.6, 35.5, 27.7.

5-(2-Methoxyphenyl)-10-(1,1-dimethylethyl)-11*H*-benzo[*b*]fluorene (13a) To 0.097 g of **9a** (0.256 mmol) in 10 mL of anhydrous toluene under a nitrogen atmosphere, was added 0.33 mL of a 1.0 M solution of potassium *t*-butoxide (0.33 mmol) in 2-methyl-2-propanol. The reaction mixture was then heated under reflux for 5 h. After the reaction mixture was allowed to cool to room temperature, 10 mL of water and 20 mL of methylene chloride were introduced. The organic layer was separated, dried over sodium sulfate and concentrated. The residue was purified by flash column chromatography (silica gel/20 % methylene chloride in hexanes) to provide 0.073 g of **13a** (0.192 mmol, 75%) as a light yellow liquid and 0.002g of **14a** (0.005 mmol, 2%). **13a**: IR 1330, 811, 739 cm^{-1}; ^{1}H (C_6D_6, 600 MHz) δ 8.62 (1 H, d, *J* = 9.0 Hz), 7.89 (1 H, d, *J* = 8.4 Hz), 7.35−7.27 (4 H, m), 7.22 (1 H, t, *J* = 7.2 Hz), 7.11 (1 H, t, *J* = 7.2 Hz), 7.04−7.02 (2 H, m), 6.86 (1 H, d, *J* = 7.8 Hz), 6.78 (1 H, d, *J* = 8.4 Hz), 4.21 (1 H, d, *J* = 21.0 Hz), 4.13 (1 H, d, *J* = 21.0 Hz), 3.06 (3 H, s), 1.71 (9 H, s); ^{13}C ($CDCl_3$, 67.9 MHz) δ 157.7, 144.2, 140.7, 140.5, 138.4, 137.7, 134.4, 131.8, 131.5, 129.6, 129.3, 128.3, 128.0, 127.2, 126.7, 126.3, 124.0, 123.8, 123.2, 123.1, 121.5, 111.6, 55.8, 40.2, 38.8, 34.4; MS *m/z* 378 (M^+), 363, 349, 321; HRMS calcd for $C_{28}H_{26}O$ 378.1984, found 378.1971. The ^{1}H NMR signals attributable to **14a** (ca. 2%) were observed at δ ($CDCl_3$) 6.25 (1 H, s), 3.86 (3 H, s), and 1.23 (9 H, s).

Trimethyl[[2-[[2-(methoxymethyl)phenyl]ethynyl]phenyl]ethynyl]silane (5b). The same procedure as described for **5a** was repeated except that 0.445 g of 1-iodo-2-[2-(trimethylsilyl)ethynyl]benzene (1.482 mmol), $Pd(PPh_3)_2Cl_2$ (0.040 g, 0.056 mmol), and copper(I) iodide (0.015 g, 0.080 mmol) in 10 mL of triethylamine were treated with a solution of 0.167 g of **4b** (1.140 mmol) in 5 mL of triethylamine to afford 0.287 g of **5b** (0.900 mmol, 79%) as a colorless liquid: ^{1}H ($CDCl_3$, 600 MHz) δ 7.31 (1 H, d, J = 7.2 Hz), 7.28−7.23 (2 H, m), 7.11 (1 H, t, J = 7.2 Hz), 7.06−7.00 (4 H, m), 4.52 (2 H, s), 3.22 (3 H, s), 0.01 (9 H, s); ^{13}C ($CDCl_3$, 150 MHz) δ 140.0, 132.6, 132.3, 131.9, 128.7, 128.2, 127.9, 127.4, 127.2, 125.9, 125.4, 121.8, 103.6, 98.6, 92.5, 90.9, 72.6, 58.5, −0.04.

Benzannulated Enediyne 6b. The same procedure as described for **6a** was repeated except that a solution of 0.255 g (0.800 mmol) **5b** in 15 mL of diethyl ether was treated with 6 mL of a 10% sodium hydroxide solution and 15 mL of methanol to afford 0.193 g of **6b** (0.785 mmol, 98%) as a yellow liquid: ^{1}H ($CDCl_3$, 600 MHz) δ 7.56 (1 H, dd, J = 7.8, 1.2 Hz), 7.55−7.53 (2 H, m), 7.50 (1 H, d, J = 8.4 Hz), 7.36 (1 H, td, J = 7.7, 1.2 Hz), 7.33 (1 H, td, J = 7.8, 1.2 Hz), 7.30−7.26 (2 H, m), 4.80 (2 H, s), 3.47 (3 H, s), 3.37 (1 H, s); ^{13}C ($CDCl_3$, 150 MHz) δ 140.3, 132.7, 132.2, 131.9, 128.8, 128.6, 128.0, 127.21, 127.18, 126.3, 124.3, 121.5, 92.2, 91.0, 82.5, 81.1, 72.6, 58.5.

α-[[2-[[2-(Methoxymethyl)phenyl]ethynyl]phenyl]ethynyl]-α-(1,1-dimethylethyl)benzenemethanol (8b). The same procedure as described for **8a** was repeated except that 0.165 g (1.015 mmol) of **7** was treated with the lithium acetylide derived from 0.178 g (0.725 mmol) of **6b** and 0.45 mL of a 1.6 M solution of *n*-butyllithium (0.72 mmol) in hexanes to afford 0.284 g (0.696 mmol, 96%) of **8b** as a light yellow liquid: IR 3410, 2961, 1490, 758 cm^{-1}; ^{1}H ($CDCl_3$, 270 MHz) δ 7.74−7.69 (2 H,

m), 7.57−7.53 (2 H, m), 7.46−7.21 (7 H, m), 4.67 (1 H, d, J =12.6 Hz), 4.40 (1 H, d, J =12.4 Hz), 3.31 (3 H, s), 1.68 (1 H, s, broad), 1.07 (9 H, s); ^{13}C ($CDCl_3$, 67.9 MHz) δ 142.4, 139.4, 132.4, 132.2, 128.5, 128.2, 128.07, 128.02, 127.8, 127.5, 127.2, 127.0, 125.6, 125.3, 122.3, 96.8, 92.6, 90.8, 84.4, 79.3, 72.5, 57.7, 39.7, 25.6.

Benzannulated Enediyne 9b. The same procedure as described for **9a** was repeated except that 0.163 g of **8b** (0.400 mmol) and 0.070 g of triethylsilane (0.601 mmol) was treated with 0.182 g of trifluoroacetic acid (1.602 mmol) to afford 0.141 g (0.360 mmol, 90%) of **9b** as a light yellow liquid: ^{1}H ($CDCl_3$, 600 MHz) δ 7.54−7.52 (1 H, m), 7.50−7.48 (1 H, m), 7.47 (1 H, d, J = 7.2 Hz), 7.41−7.38 (3 H, m), 7.34 (1 H, t, J =7.8 Hz), 7.29−7.27 (2 H, m), 7.21 (1 H, t, J =7.8 Hz), 7.19−7.17 (3 H, m), 4.70 (1 H, d, J =12.6 Hz), 4.63 (1 H, d, J =13.2 Hz), 3.69 (1 H, s), 3.41 (3 H, s), 1.03 (9 H, s); ^{13}C ($CDCl_3$, 150 MHz) δ 140.2, 139.1, 132.3, 132.11, 132.05, 129.7, 128.5, 128.0, 127.6, 127.4, 127.2, 127.1, 126.7, 126.3, 125.5, 121.7, 95.7, 93.0, 90.3, 82.5, 72.5, 58.5, 50.6, 35.5, 27.7.

5-[2-(Methoxymethyl)phenyl]-10-(1,1-dimethylethyl)-11*H*-benzo[*b*]fluorene (13b). The same procedure as described for **13a** was repeated except that 0.119 g of **9b** (0.303 mmol) was treated with 0.39 mL of a 1.0 M solution of potassium *t*-butoxide (0.39 mmol) in 2-methyl-2-propanol to afford 0.075 g of a mixture of **13b** and **14b** in a 5:1 ratio as a light yellow liquid. The mixture was then dissolved in 8 mL of THF and treated with 1 mL of a 1.0 M $BH_3 \cdot THF$ solution under a nitrogen atmosphere. After stirring at room temperature for 5 h, 10 mL of water and 10 mL of diethyl ether were added. The organic layer was separated and the aqueous layer back extracted with diethyl ether. The combined organic layers were washed successively with brine and water, dried over

sodium sulfate, and concentrated. Purification of the residue by flash column chromatography (silica gel/30% methylene chloride in hexanes) afforded 0.057 g of **13b** (0.145 mmol, 48%) as a colorless liquid: IR 2923, 1088, 764, 732 cm^{-1}; ^{1}H ($CDCl_3$, 600 MHz) δ 8.62 (1 H, d, *J* = 9.6 Hz), 7.77 (1 H, d, *J* = 7.8 Hz), 7.60 (1 H, t, *J* = 7.8 Hz), 7.49−7.41 (4 H, m), 7.29 (1 H, t, *J* = 7.2 Hz), 7.22 (1 H, d, *J* = 7.8 Hz), 7.19 (1 H, t, *J* = 7.2 Hz), 6.96 (1 H, t, *J* = 7.8 Hz), 6.22 (1 H, d, *J* = 7.8 Hz), 4.522 (1 H, d, *J* = 21 Hz), 4.504 (1 H, d, *J* = 21 Hz), 4.07 (1 H, d, *J* = 13.8 Hz), 4.04 (1 H, d, *J* = 13.8 Hz), 3.10 (3 H, s), 1.93 (9 H, s); ^{13}C ($CDCl_3$, 67.9 MHz) δ 144.1, 141.2, 140.1, 138.3, 137.9, 137.5, 137.4, 134.1, 131.4, 130.7, 130.3, 128.2, 128.0, 127.0, 126.5, 124.3, 123.9, 123.4, 123.1, 71.7, 58.3, 40.2, 38.9, 34.4; MS *m/z* 392 (M$^+$), 377, 343; HRMS calcd for $C_{29}H_{28}O$ 392.2140, found 392.2135. The ^{1}H NMR signals attributable to **14b** (ca. 10%) before treatment with BH_3·THF were observed at δ ($CDCl_3$) 6.36 (1 H, s), 4.31 (1 H, d, *J* = 13.2 Hz), 3.83 (1 H, d, *J* = 13.2 Hz), 2.9 (3 H, s), and 1.21 (9 H, s).

Benzannulated Enediyne 19a. To a mixture of 0.193 g of **17** (0.516 mmol), $Pd(PPh_3)_2Cl_2$ (0.018 g, 0.026 mmol), and copper(I) iodide (0.010 g, 0.053 mmol) in 10 mL of triethylamine, was added via cannula a solution of 0.102 g of **18a** (0.670 mmol) in 3 mL of triethylamine. After stirring at 60 °C for 12 h, 15 mL of a saturated ammonium chloride solution and 15 mL of diethyl ether were added. The organic layer was separated and the aqueous layer back extracted with diethyl ether. The combined organic layers were washed successively with brine and water, dried over sodium sulfate, and concentrated. Purification of the residue by flash column chromatography (silica gel/5% methylene chloride in hexanes) afforded 0.189 g of **19a** (0.475 mmol, 92%) as a light yellow liquid: IR 2227, 1481, 758 cm^{-1}; ^{1}H ($CDCl_3$, 270 MHz) δ 8.53−8.46 (1 H, m),

7.90−7.82 (2 H, m), 7.68−7.62 (2 H, m), 7.56−7.48 (3 H, m), 7.44−7.38 (3 H, m), 7.34−7.30 (2 H, m), 7.16−7.12 (3 H, m) 3.71 (1 H, s), 1.01 (9 H, s); ^{13}C ($CDCl_3$, 67.9 MHz) δ 138.9, 133.2, 133.1, 132.3, 132.1, 130.4, 129.7, 128.7, 128.1, 128.0, 127.5, 127.4, 126.7, 126.6, 126.4, 126.3, 125.6, 125.2, 120.9, 95.8, 93.4, 90.9, 82.6, 50.6, 35.5, 27.7.

10-(1,1-Dimethylethyl)-5-(1-naphthyl)-11*H*-benzo[*b*]fluorene (20a). To 0.189 g of **19a** (0.475 mmol) in 10 mL of anhydrous toluene under a nitrogen atmosphere, was added 0.50 mL of a 1.0 M solution of potassium *t*-butoxide (0.50 mmol) in 2-methyl-2-propanol. The reaction mixture was then heated under reflux for 6 h. After the reaction mixture was allowed to cool to room temperature, 10 mL of water and 40 mL of methylene chloride were introduced, the organic layer separated, dried over sodium sulfate, and concentrated. The residue was purified by flash column chromatography (silica gel/5 % methylene chloride in hexanes) to provide 0.131 g of **20a** (0.328 mmol, 69%) as a bright yellow liquid: IR 1191, 1015 cm^{-1}; ^{1}H (C_6D_6, 600 MHz) δ 8.68 (1 H, d, *J* = 9.0 Hz), 7.84 (1 H, d, *J* =7.8 Hz), 7.76 (1 H, d, *J* = 8.4 Hz), 7.60 (1 H, dd, *J* = 8.4, 1.2 Hz), 7.54 (1 H, d, *J* = 8.4 Hz), 7.45 (1 H, dd, *J* = 6.6, 1.2 Hz), 7.41 (1 H, dd, *J* = 8.1, 6.9 Hz), 7.27 (1 H, ddd, *J* = 9.0, 6.6, 1.2 Hz), 7.23 (1 H, d, *J* = 7.2 Hz), 7.19 (1 H, ddd, *J* = 8.4, 6.6, 1.2 Hz), 7.01 (1 H, ddd, *J* = 8.4, 6.6, 1.2 Hz), 6.96 (1 H, td, *J* = 7.2, 1.2 Hz), 6.90 (1 H, ddd, *J* = 8.4, 6.6, 1.2 Hz), 6.65 (1 H, t, *J* = 7.2 Hz), 6.32 (1 H, d, *J* = 7.8 Hz), 4.27 (1 H, d, *J* = 21 Hz), 4.25 (1 H, d, *J* = 21 Hz), 1.81 (9 H, s); ^{1}H ($CDCl_3$, 270 MHz) δ 8.66 (1 H, d, *J* = 8.7 Hz), 8.07 (1 H, d, *J* = 8.4 Hz), 8.00 (1 H, d, *J* = 8.4 Hz), 7.68 (1 H, t, *J* = 7.7 Hz), 7.52−7.15 (10 H, m), 7.11 (1 H, td, *J* = 7.4, 1.0 Hz), 6.75 (1 H, t, *J* = 7.4 Hz), 5.89 (1 H, d, *J* = 8.2 Hz), 4.56 (2 H, s), 1.96 (9 H, s); ^{13}C ($CDCl_3$, 67.9 MHz) δ 144.2, 141.2, 139.9, 139.1, 137.6, 137.4, 134.9, 133.8, 132.8, 131.4, 130.8, 128.16, 128.03,

127.97, 127.88, 127.7, 126.7, 126.3, 126.2, 124.2, 123.8, 123.5, 123.4, 40.2, 38.9, 34.4; MS *m/z* 398 (M^+), 383, 341; HRMS calcd for $C_{31}H_{26}$ 398.2035, found 398.2028.

1-Ethynyl-2-methoxynaphthalene (18b). To a solution of 1.05 g (5.64 mmol) of 2-methoxy-1-naphthaldehyde and 2.80 g of potassium carbonate in 20 mL of anhydrous methanol, was added 1.37 g (7.15 mmol) of dimethyl (1-diazo-2-oxopropyl)phosphonate, and the reaction mixture was stirred at room temperature for 6 days. The reaction mixture was then diluted with diethyl ether, washed with a 5% aqueous sodium bicarbonate solution, dried over sodium sulfate and concentrated. Purification of the residue by flash column chromatography (silica gel/25% methylene chloride in hexanes) afforded 0.545 g of **18b** (3.00 mmol, 53%) as a white solid: IR 3260, 1274, 1083, 809 cm^{-1}; ^1H ($CDCl_3$, 270 MHz) δ 8.27 (1 H, d, J = 8.4 Hz), 7.86 (1 H, d, J = 8.9 Hz), 7.79 (1 H, d, J = 8.2 Hz), 7.56 (1 H, ddd, J = 8.2, 6.9, 1.2 Hz), 7.39 (1 H, ddd, J = 8.2, 6.9, 1.2 Hz), 7.27 (1 H, d, J = 9.2 Hz), 4.05 (3 H, s), 3.76 (1 H, s); ^{13}C ($CDCl_3$, 150 MHz) δ 159.8, 134.8, 130.7, 128.4, 128.1, 127.6, 125.1, 124.3, 112.5, 105.0, 86.5, 78.2, 56.6; HRMS calcd for $C_{13}H_{11}O$ (MH^+) 183.0810, found 183.0804.

Benzannulated Enediyne 21. The same procedure as described for **5a** was repeated except that 0.200 g of **17** (0.535 mmol), $Pd(PPh_3)_2Cl_2$ (0.016 g, 0.023 mmol), and copper(I) iodide (0.015 g, 0.080 mmol) in 10 mL of triethylamine was treated with a solution of 0.263 g of (trimethylsilyl)acetylene (2.675 mmol) in 5 mL of triethylamine to afford 0.140 g of **21** (0.407 mmol, 76%) as a colorless liquid: ^1H ($CDCl_3$, 270 MHz) δ 7.50−7.39 (4 H, m), 7.35−7.20 (5 H, m), 3.69 (1 H, s), 1.09 (9 H, s), 0.24 (9 H, s); ^{13}C ($CDCl_3$, 67.9 MHz) δ 139.1, 132.8, 132.2, 129.8, 128.0, 127.6, 127.2, 126.6, 126.4, 125.3, 104.0, 98.0, 95.5, 82.3, 50.6, 35.5, 27.9, −0.1.

Benzannulated Enediyne 22. The same procedure as described for **6a** was repeated except that a solution of 0.140 g (0.407 mmol) of **21** in 20 mL of diethyl ether was treated with 8 mL of a 10% sodium hydroxide solution and 20 mL of methanol to afford 0.110 g of **22** (0.403 mmol, 99%) as a light yellow liquid: ^{1}H ($CDCl_3$, 270 MHz) δ 7.55−7.44 (4 H, m), 7.38−7.22 (5 H, m), 3.72 (1 H, s), 3.31 (1 H, s), 1.11 (9 H, s); ^{13}C ($CDCl_3$, 67.9 MHz) δ 139.1, 132.5, 132.0, 129.8, 128.4, 127.5, 127.3, 127.1, 126.7, 124.4, 95.7, 82.8, 82.0, 80.7, 50.5, 35.5, 27.8.

1-Iodo-2-(methoxymethyl)naphthalene (23). A solution of *n*-butyllithium in hexanes (1.6 M, 1.8 mL, 3.06 mmol) was added dropwise to a solution of 0.500 g of 1-bromo-2-(methoxymethyl)naphthalene (1.99 mmol) in 10 mL of THF at −78 °C. After stirring at −78 °C for 1 h, a solution of 1.02 g of iodine (4.02 mmol) in 10 mL of THF was added dropwise via cannula. The reaction mixture then was allowed to warm to room temperature before the addition of 20 mL of a 5% sodium thiosulfate solution. The organic layer was separated and the aqueous layer back extracted with diethyl ether. The combined organic layers were washed successively with brine and water, dried over sodium sulfate, and concentrated. Purification of the residue by flash column chromatography (silica gel/30% methylene chloride in hexanes) afforded 0.516 g of **23** (1.73 mmol, 87%) as a pale yellow liquid: IR 2922, 1500, 1113, 814 cm^{-1}; ^{1}H ($CDCl_3$, 270 MHz) δ 8.26 (1 H, d, *J* = 7.7 Hz), 7.83 (1 H, d, *J* = 8.4 Hz), 7.78 (1 H, dd, *J* = 7.9, 1.5 Hz), 7.61−7.48 (3 H, m), 4.74 (2 H, s), 3.53 (3 H, s); ^{13}C ($CDCl_3$, 67.9 MHz) δ 139.8, 134.6, 133.6, 132.2, 128.7, 128.3, 127.7, 126.4, 125.8, 103.1, 79.8, 58.6.

1,1′-Binaphthyl 24. Boron tribromide (0.5 mL) was added dropwise to a solution of **20b** (0.234 g, 0.546 mmol) in 10 mL of methylene chloride of at 0 °C. The reaction mixture

was stirred at 0 °C for 2 h before 10 mL of water and 20 mL of methylene chloride were introduced. The organic layer was separated, dried over sodium sulfate and concentrated. The residue was purified by flash column chromatography (silica gel/50% methylene chloride in hexanes) to provide 0.172 g (0.42 mmol, 76%) of **24** as a pale yellow solid: ^{1}H ($CDCl_3$, 270 MHz) δ 8.69 (1 H, d, J = 8.9 Hz), 8.03 (1 H, d, J = 8.9 Hz), 7.92 (1 H, d, J = 7.9 Hz), 7.47 (1 H, d, J = 7.4 Hz), 7.48−7.12 (8 H, m), 7.05 (1 H, d, J = 8.4 Hz), 6.83 (1 H, t, J = 7.4 Hz), 6.21 (1 H, d, J = 7.9 Hz), 4.89 (1 H, br s), 4.59 (1 H, d, J = 21 Hz), 4.54 (1 H, d, J = 21 Hz), 1.97 (9 H, s); ^{13}C ($CDCl_3$, 67.9 MHz) δ 151.1, 144.2, 142.8, 141.1, 139.3, 138.2, 134.6, 133.5, 132.0, 130.1, 129.3, 128.3, 128.1, 127.5, 126.8, 126.6, 125.1, 124.8, 124.1, 123.9, 123.6, 123.0, 117.6, 117.5, 40.3, 39.1, 34.4; HRMS calcd for $C_{31}H_{26}ONa$ (MNa^+) 437.1881, found 437.1877.

(1*S*)-Camphanates of 24. To a solution of 0.039 g of **24** (0.095 mmol) and 0.2 mL of triethylamine (0.4 mmol) in 3 mL of anhydrous methylene chloride at room temperature under a nitrogen atmosphere, was added 0.062 g of (1*S*)-(−)-camphanoyl chloride (0.29 mmol). The mixture was stirred for 12 h and then quenched with a saturated sodium bicarbonate solution. After 5 mL of methylene chloride was added, the organic layer was separated and the aqueous layer back extracted with methylene chloride. The combined organic extracts were washed successively with brine and water, dried over sodium sulfate, and concentrated. The residue was purified by flash column chromatography (silica gel/20% ethyl acetate in hexanes). A small fraction of the eluent contained partially separated (1*S*)-camphanates in a 5:1 ratio. The solvent of this fraction was evaporated to afford a white solid: ^{1}H ($CDCl_3$, 600 MHz) δ 8.54 (1 H, d, J = 9.0 Hz), 8.08 (1 H, d, J = 9.0 Hz), 7.96 (1 H, d, J = 8.4 Hz), 7.48 (1 H, dd, J = 9.0, 1.8 Hz), 7.43 (1 H,

t, *J* = 7.2 Hz), 7.39−7.32 (2 H, m), 7.29−7.16 (4 H, m), 7.13 (1 H, t, *J* = 7.2 Hz), 7.05 (1 H, t, *J* = 7.8 Hz), 5.96 (1 H, d, *J* = 8.4 Hz), 4.47 (1 H, d, *J* = 21.6 Hz), 4.41 (1 H, d, *J* = 21.0 Hz), 1.87 (9 H, s), 1.63−1.57 (1 H, m), 1.50−1.45 (2 H, m), 1.38−1.32 (1 H, m), 0.80 (3 H, s), 0.07 (3 H, s), 0.00 (3 H, s). A minor set of the ^{1}H NMR signals at δ 5.93 (1 H, d, *J* = 8.4 Hz), 4.48 (1 H, d, *J* = 21.6 Hz), 4.43 (1 H, d, *J* = 21.0 Hz), 0.02 (3 H, s), and 0.01 (3 H, s) attributable to the other camphanate diastereomer were also observed.

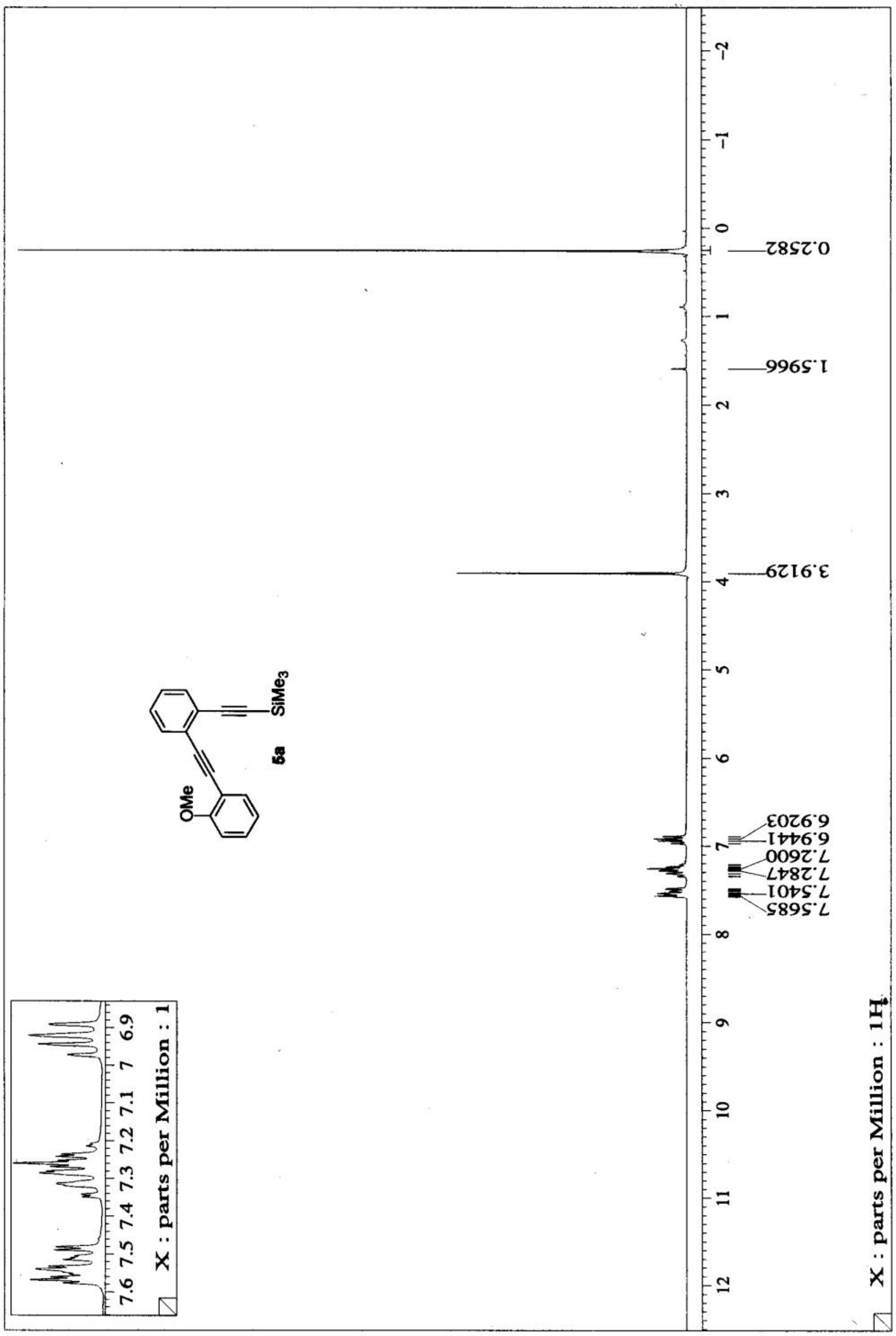

5a
OMe
SiMe3
0.2582
1.5966
3.9129
6.9203
6.9441
7.2600
7.2847
7.5401
7.5685
X : parts per Million : 1H
X : parts per Million : 1H

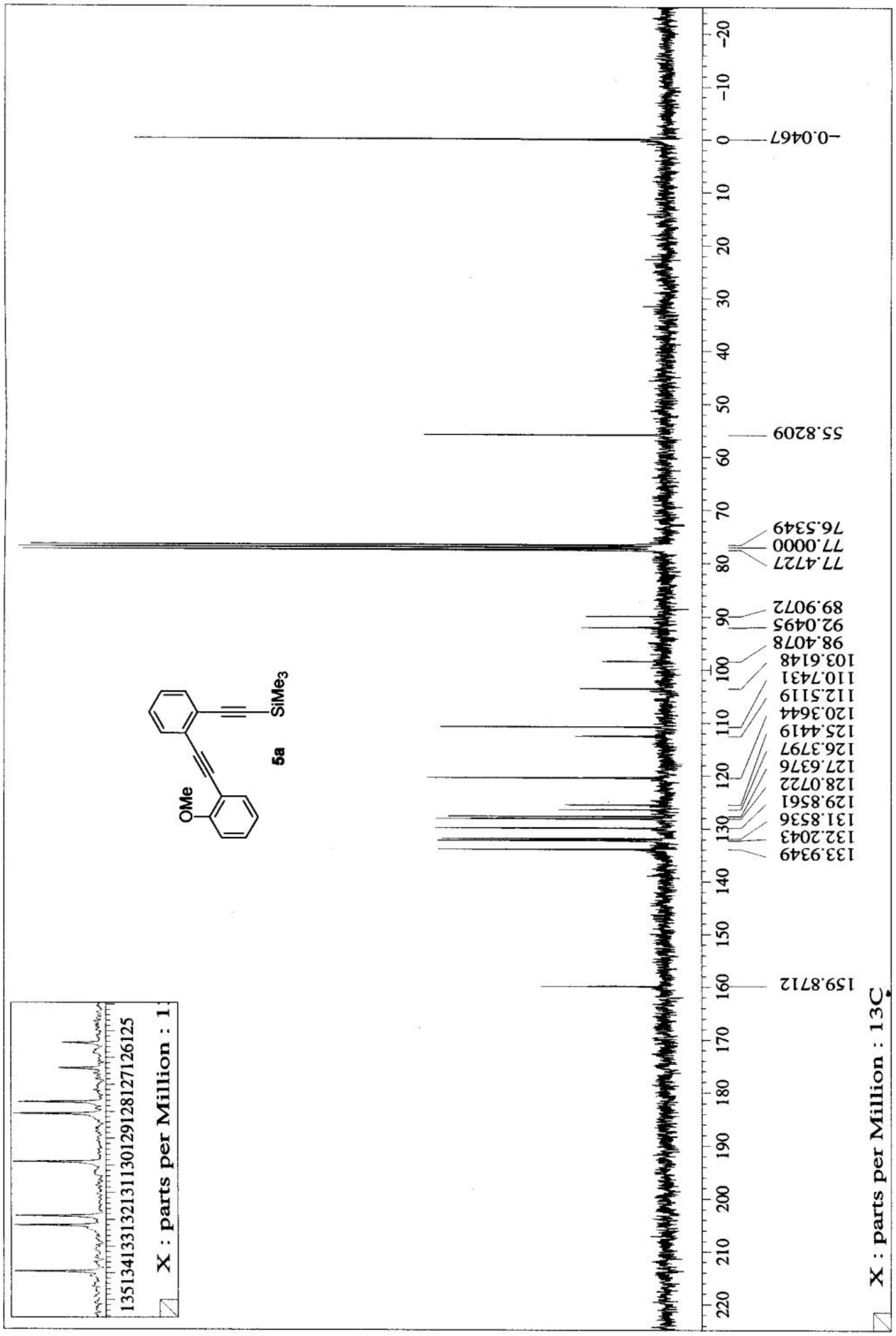
OMe
SiMe3
5a
-0.0467
55.8209
76.5349
77.0000
77.4727
89.9072
92.0495
98.4078
103.6148
110.7431
112.5119
120.3644
125.4419
126.3797
127.6376
128.0722
129.8561
131.8536
132.2043
133.9349
159.8712
X : parts per Million : 13C
X : parts per Million : 1

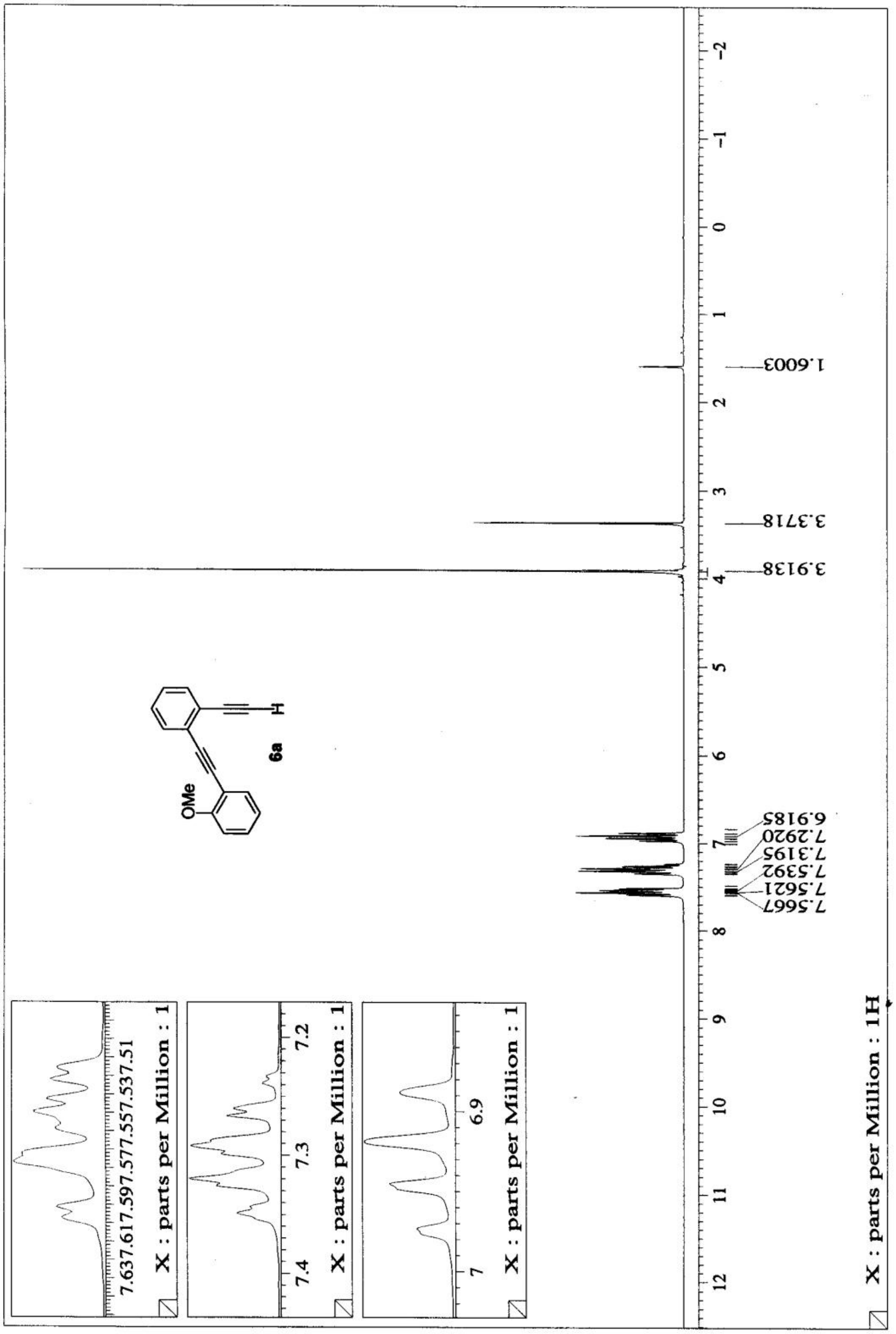

OMe
6a
1.6003
3.3718
3.9138
6.9185
7.2920
7.3195
7.5392
7.5621
7.5667
X : parts per Million : 1H

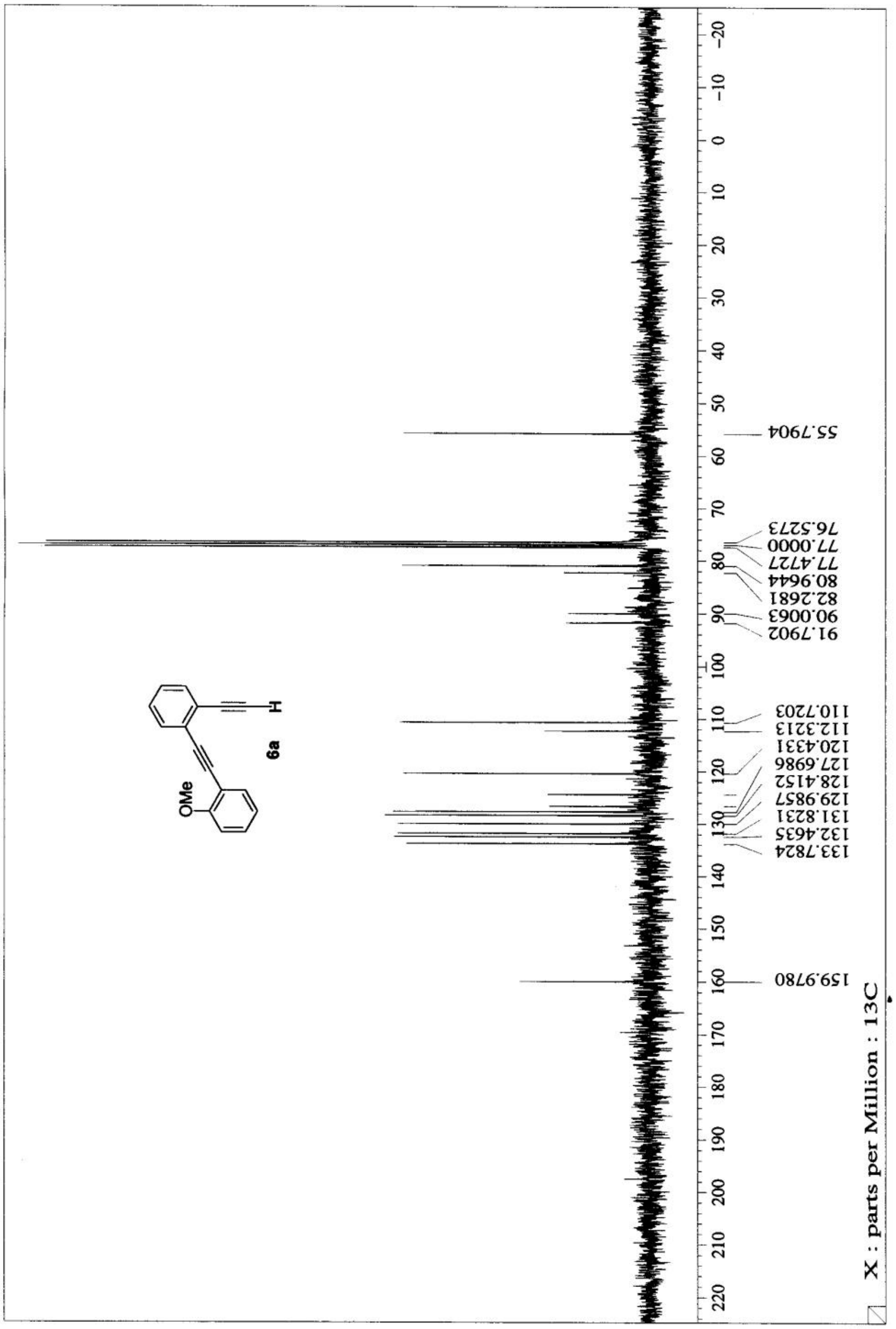
OMe
H
6a
55.7904
76.5273
77.0000
77.4727
80.9644
82.2681
90.0063
91.7902
110.7203
112.3213
120.4331
127.6986
128.4152
129.9857
131.8231
132.4635
133.7824
159.9780
X : parts per Million : 13C

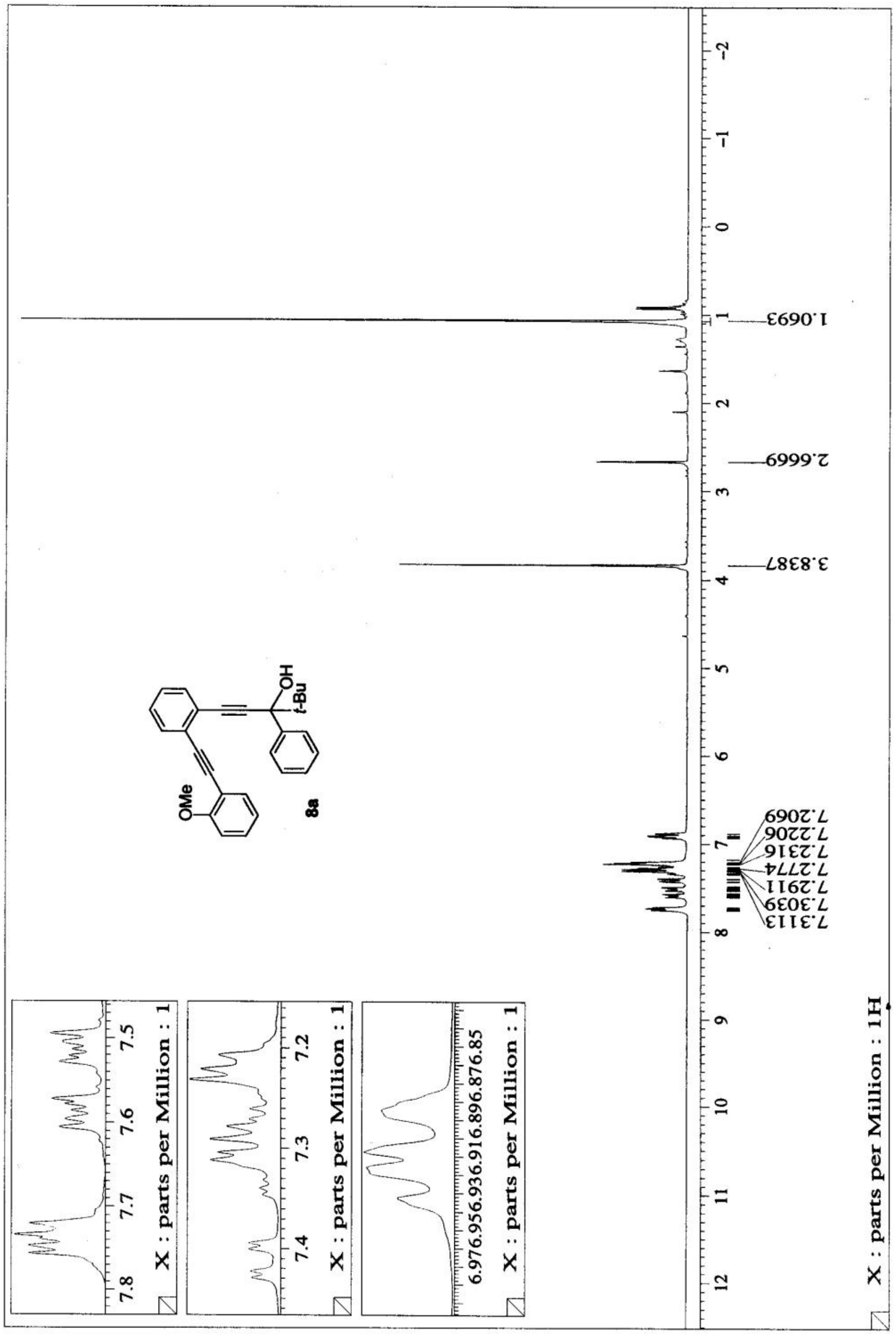
OMe
OH
t-Bu
8a
1.0693
2.6669
3.8387
7.2069
7.2206
7.2316
7.2774
7.2911
7.3039
7.3113
X : parts per Million : 1H

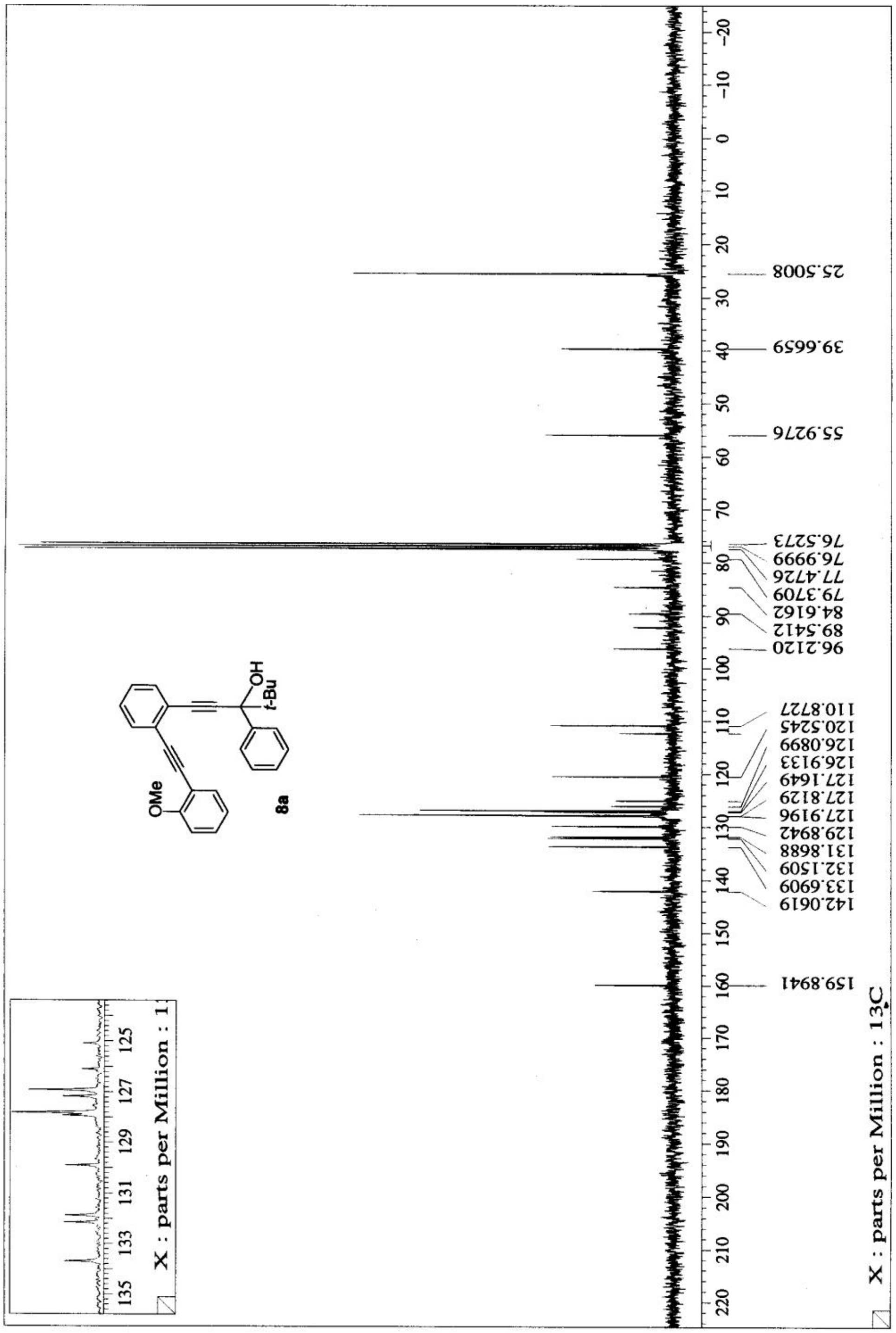
OMe
OH
t-Bu
8a
25.5008
39.6659
55.9276
76.5273
76.9999
77.4726
79.3709
84.6162
89.5412
96.2120
110.8727
120.5245
126.0899
126.9133
127.1649
127.8129
127.9196
129.8942
131.8688
132.1509
133.6909
142.0619
159.8941
X : parts per Million : 13C
X : parts per Million : 1

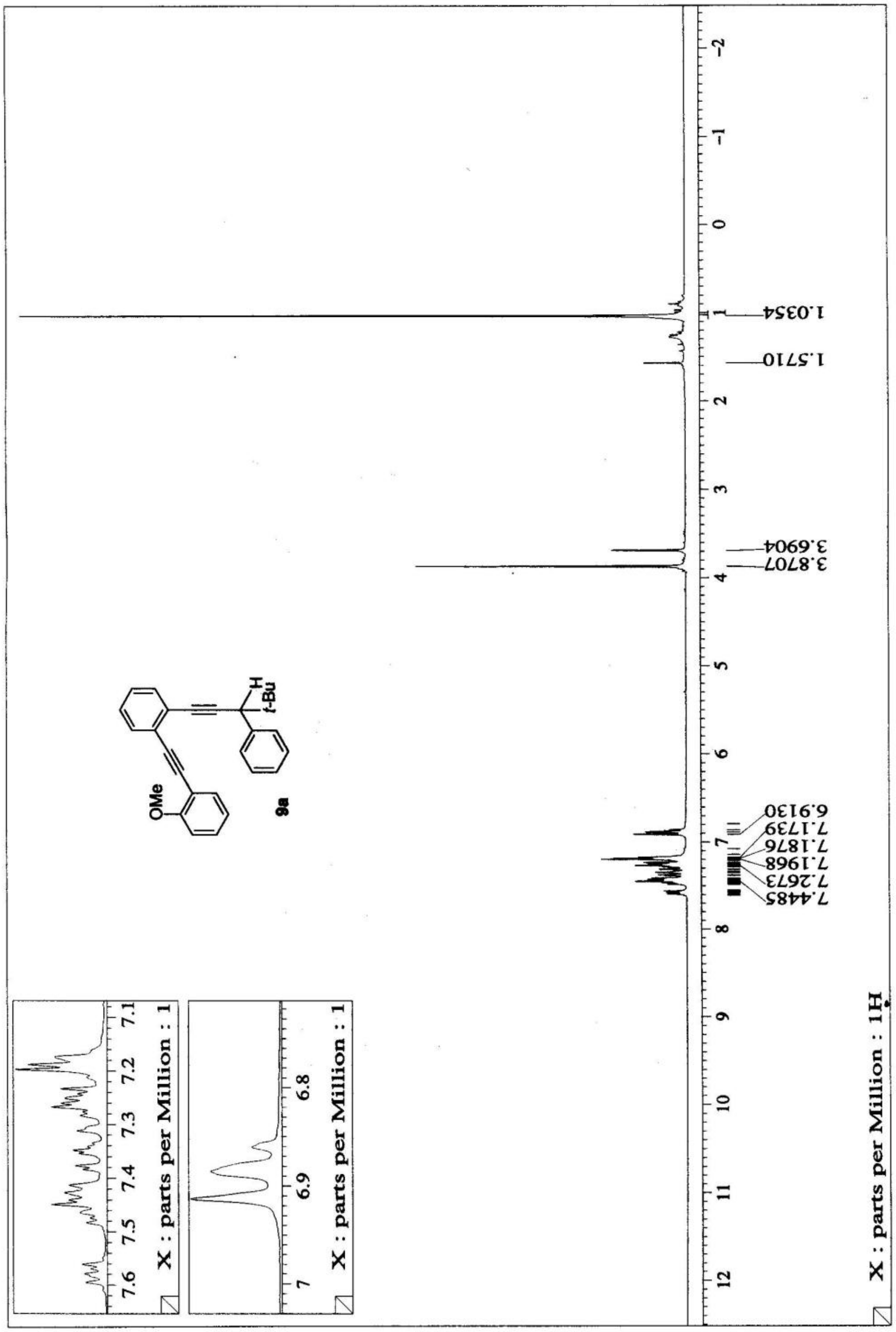
OMe
t-Bu
H
9a
1.0354
1.5710
3.6904
3.8707
6.9130
7.1739
7.1876
7.1968
7.2673
7.4485
X : parts per Million : 1H
X : parts per Million : 1
X : parts per Million : 1

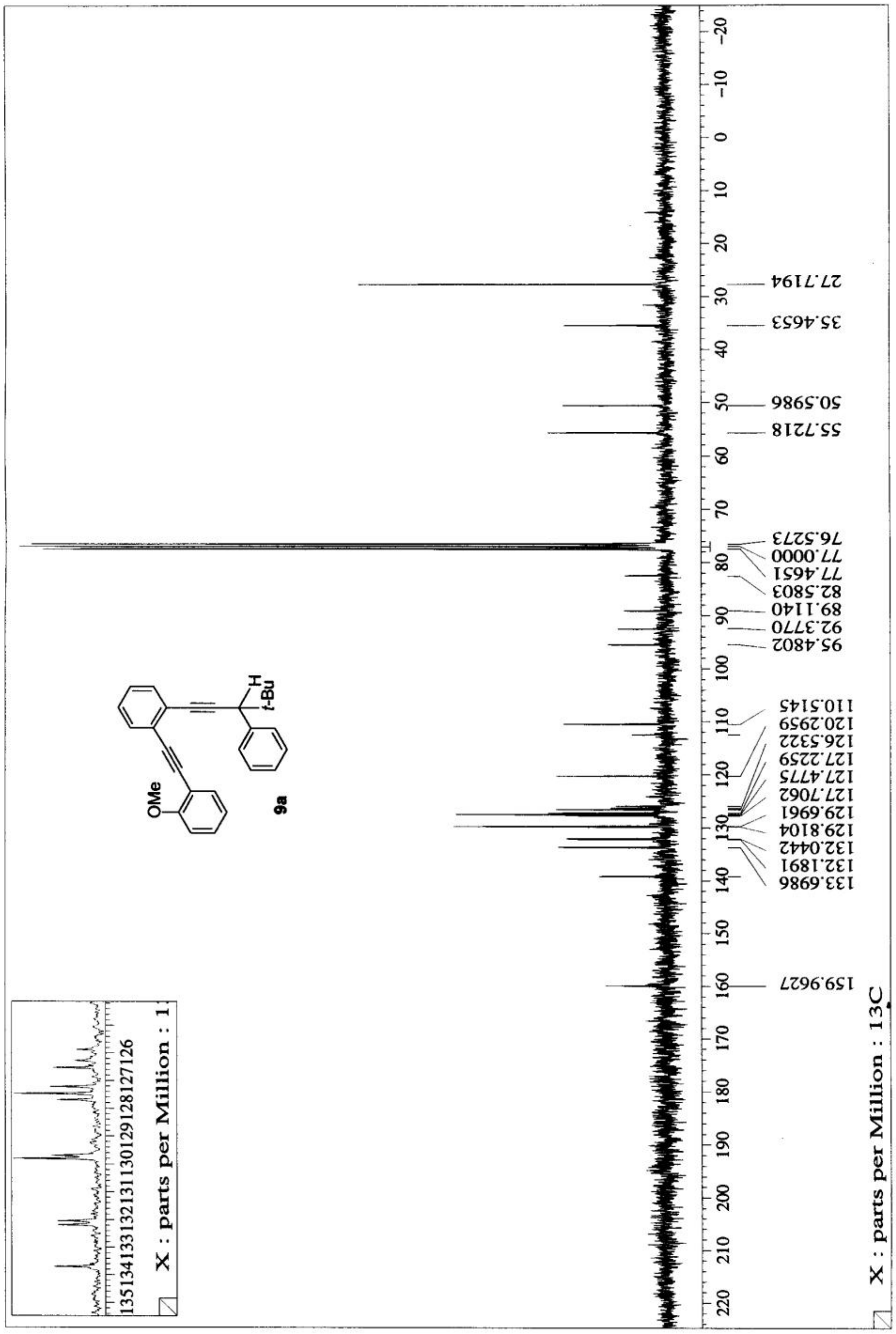

OMe
H
t-Bu
9a
27.7194
35.4653
50.5986
55.7218
76.5273
77.0000
77.4651
82.5803
89.1140
92.3770
95.4802
110.5145
120.2959
126.5322
127.2259
127.4775
127.7062
129.6961
129.8104
132.0442
132.1891
133.6986
159.9627
X : parts per Million : 13C
X : parts per Million : 1

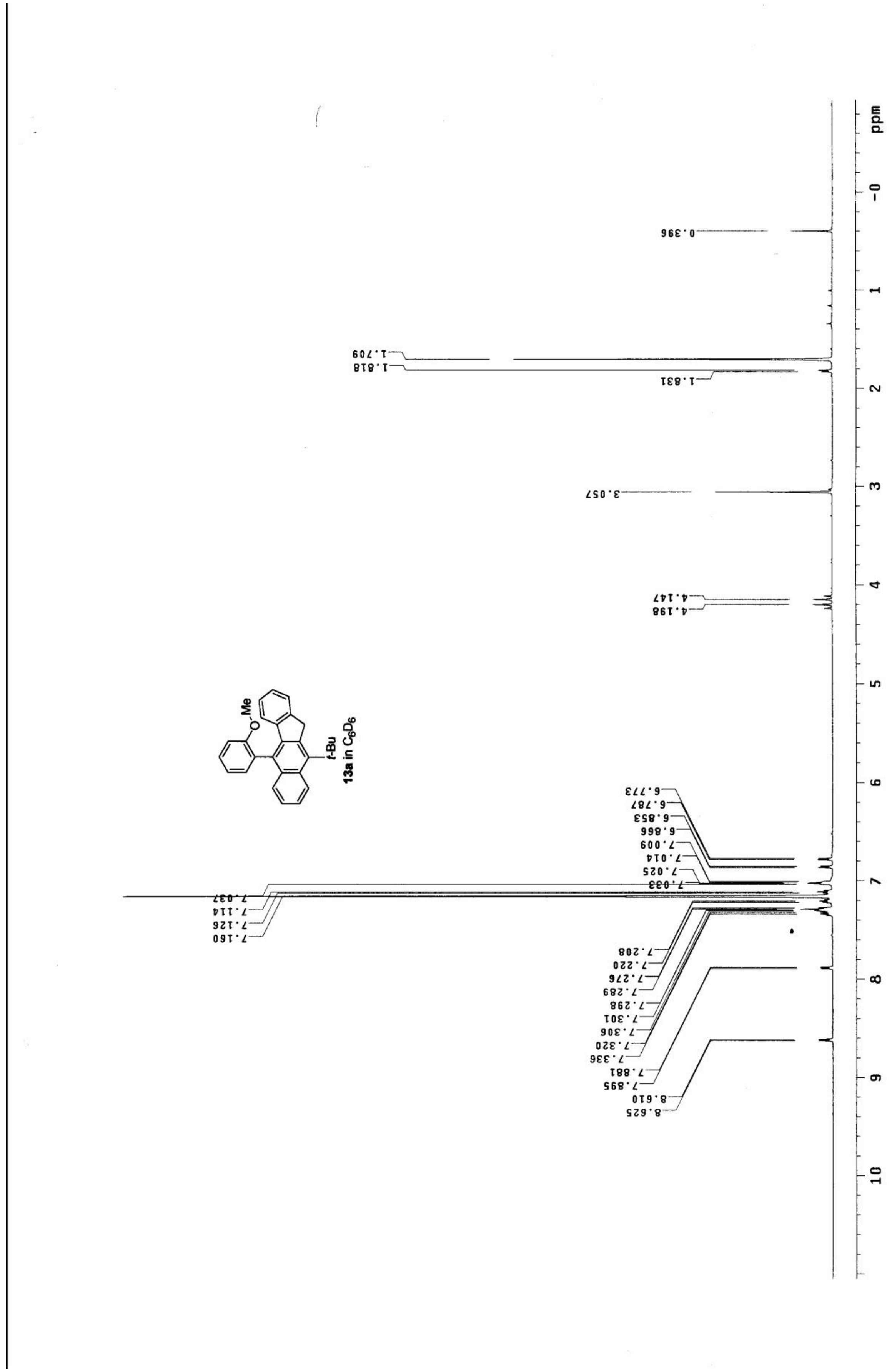

13a in C_6D_6
t-Bu
Me
O
0.396
1.709
1.818
1.831
3.057
4.147
4.198
6.773
6.787
6.853
6.866
7.009
7.014
7.025
7.033
7.037
7.114
7.126
7.160
7.208
7.220
7.276
7.289
7.298
7.301
7.306
7.320
7.336
7.881
7.895
8.610
8.625
ppm

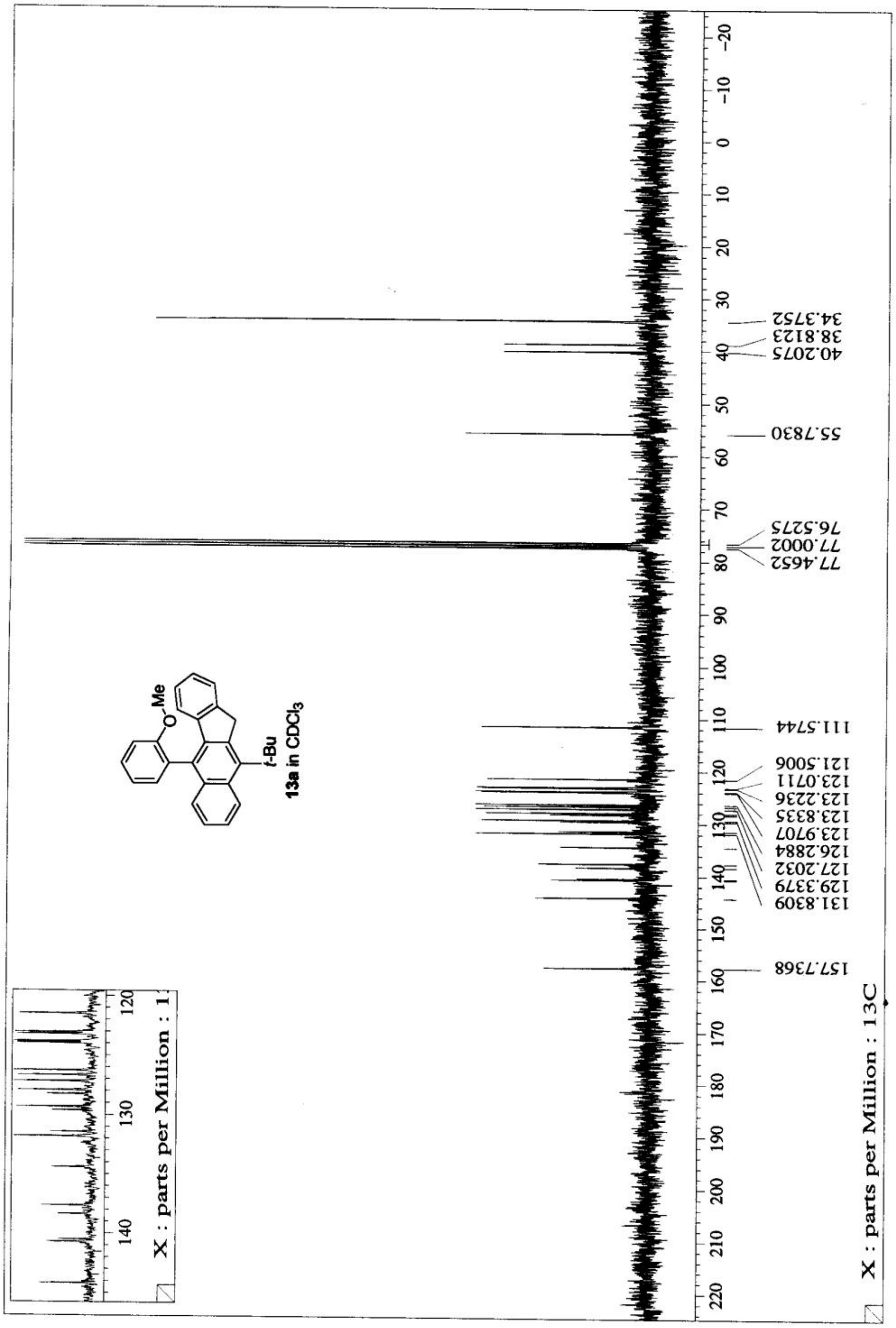

OMe
t-Bu
13a in CDCl3
34.3752
38.8123
40.2075
55.7830
76.5275
77.0002
77.4652
111.5744
121.5006
123.0711
123.2236
123.8335
123.9707
126.2884
127.2032
129.3379
131.8309
157.7368
X : parts per Million : 13C

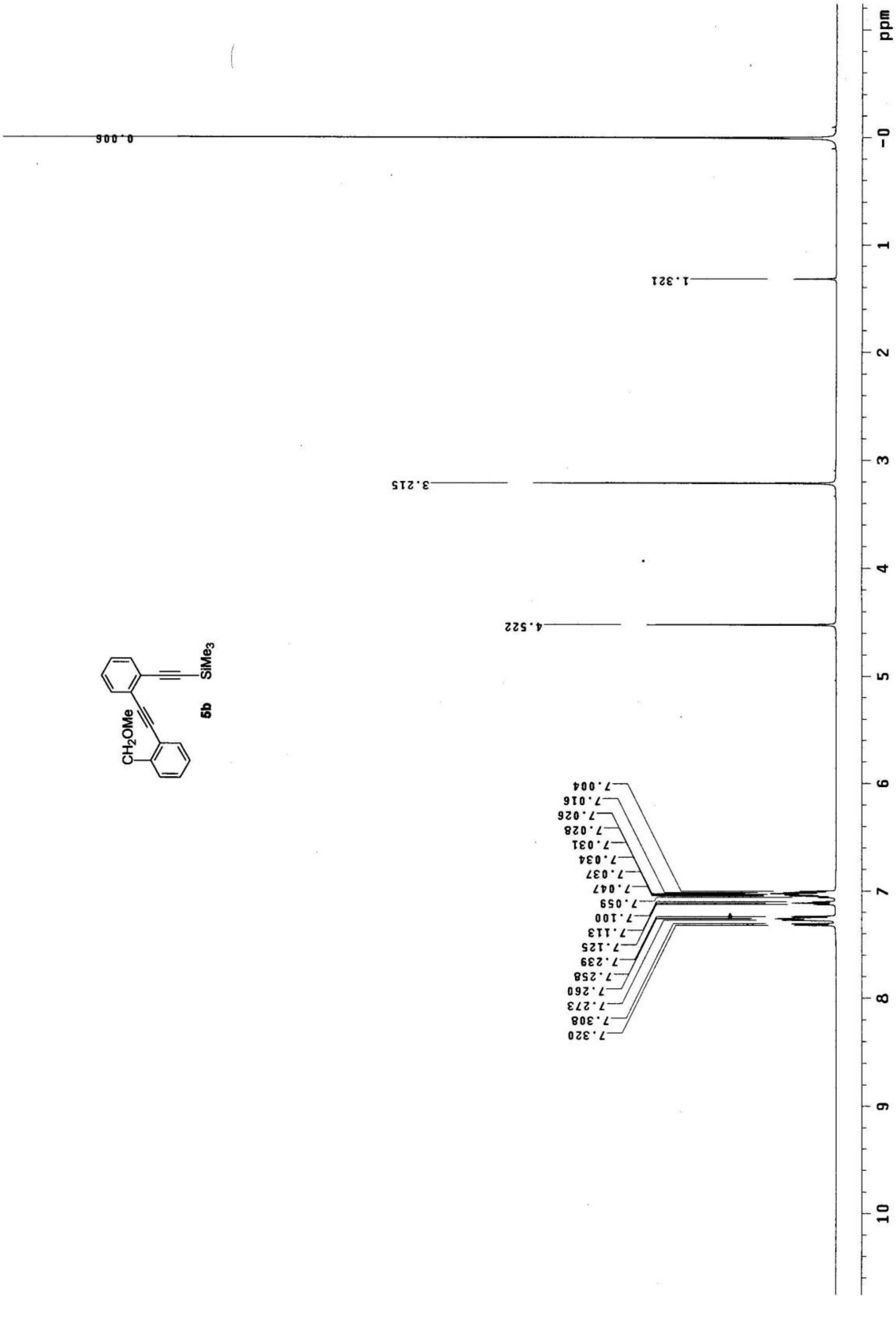

5b
CH2OMe
SiMe3
0.006
1.321
3.215
4.522
7.004
7.016
7.026
7.028
7.031
7.034
7.037
7.047
7.059
7.100
7.113
7.125
7.239
7.258
7.260
7.273
7.308
7.320
ppm

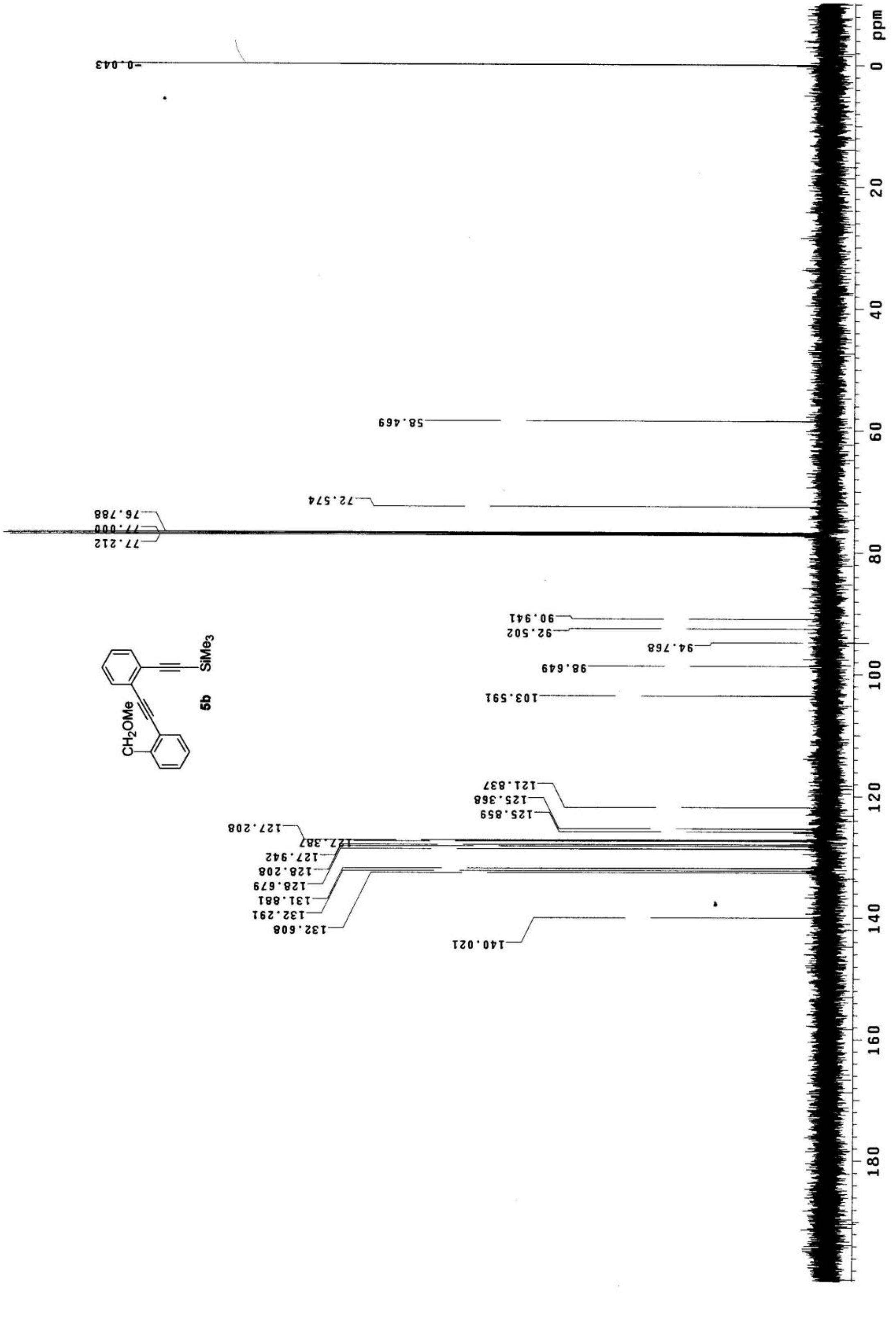
-0.043
58.469
72.574
76.788
77.000
77.212
90.941
92.502
94.768
98.649
103.591
121.837
125.368
125.859
127.208
127.387
127.942
128.208
128.679
131.881
132.291
132.608
140.021
CH2OMe
SiMe3
5b
ppm

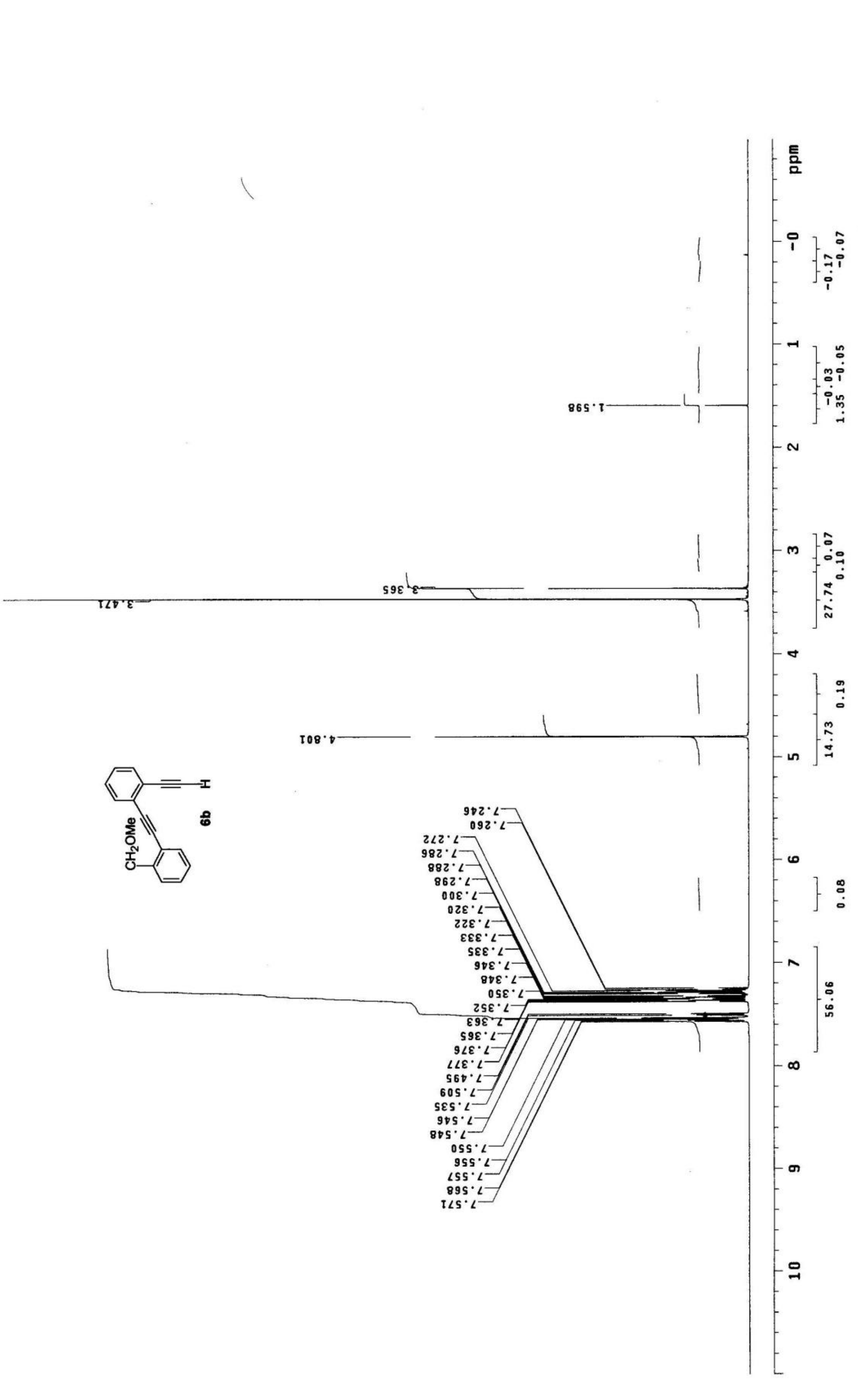

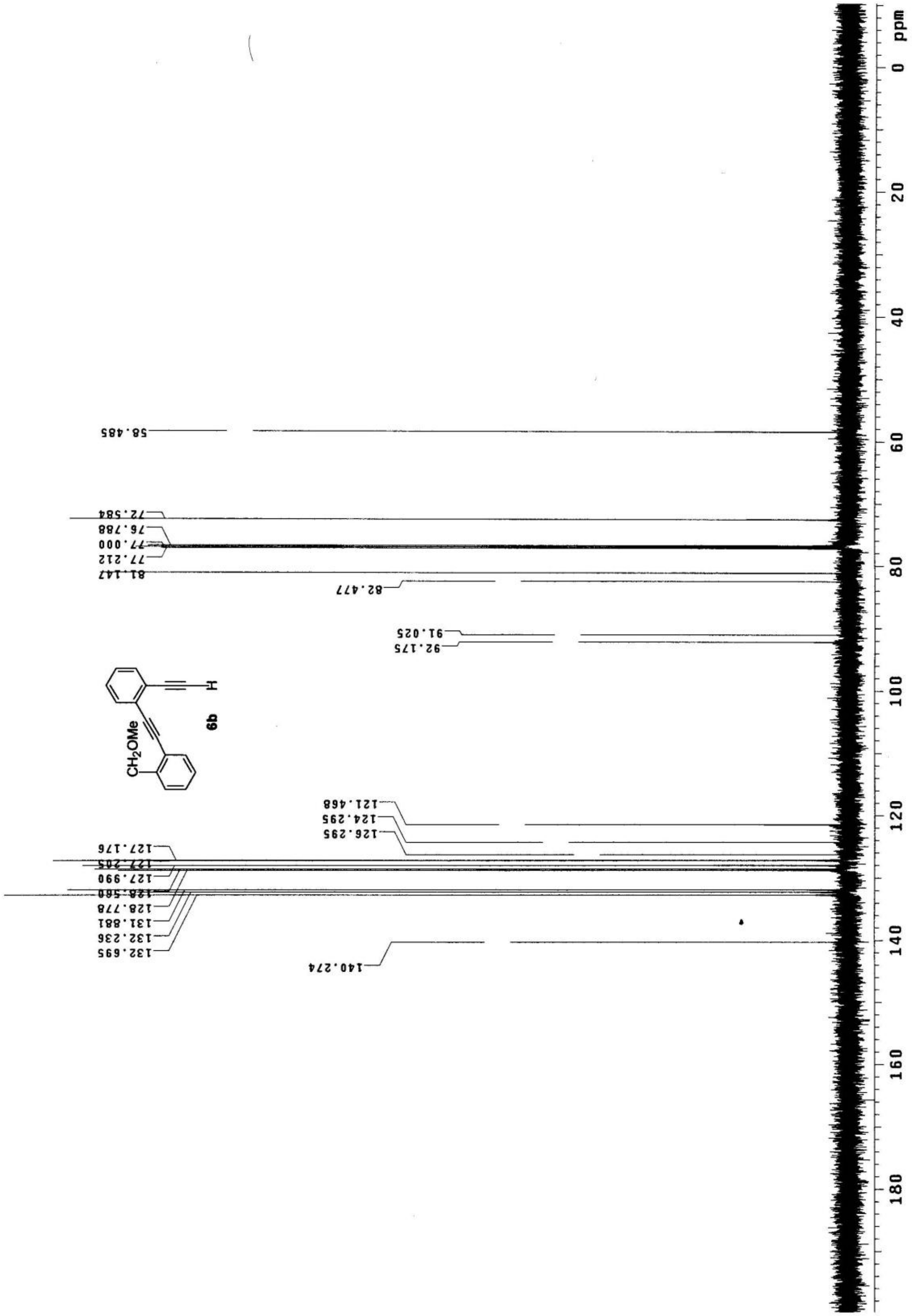
58.485
72.584
76.788
77.000
77.212
81.147
82.477
91.025
92.175
121.468
124.295
126.295
127.176
127.205
127.990
128.560
128.778
131.881
132.236
132.695
140.274
CH2OMe
H
6b
ppm

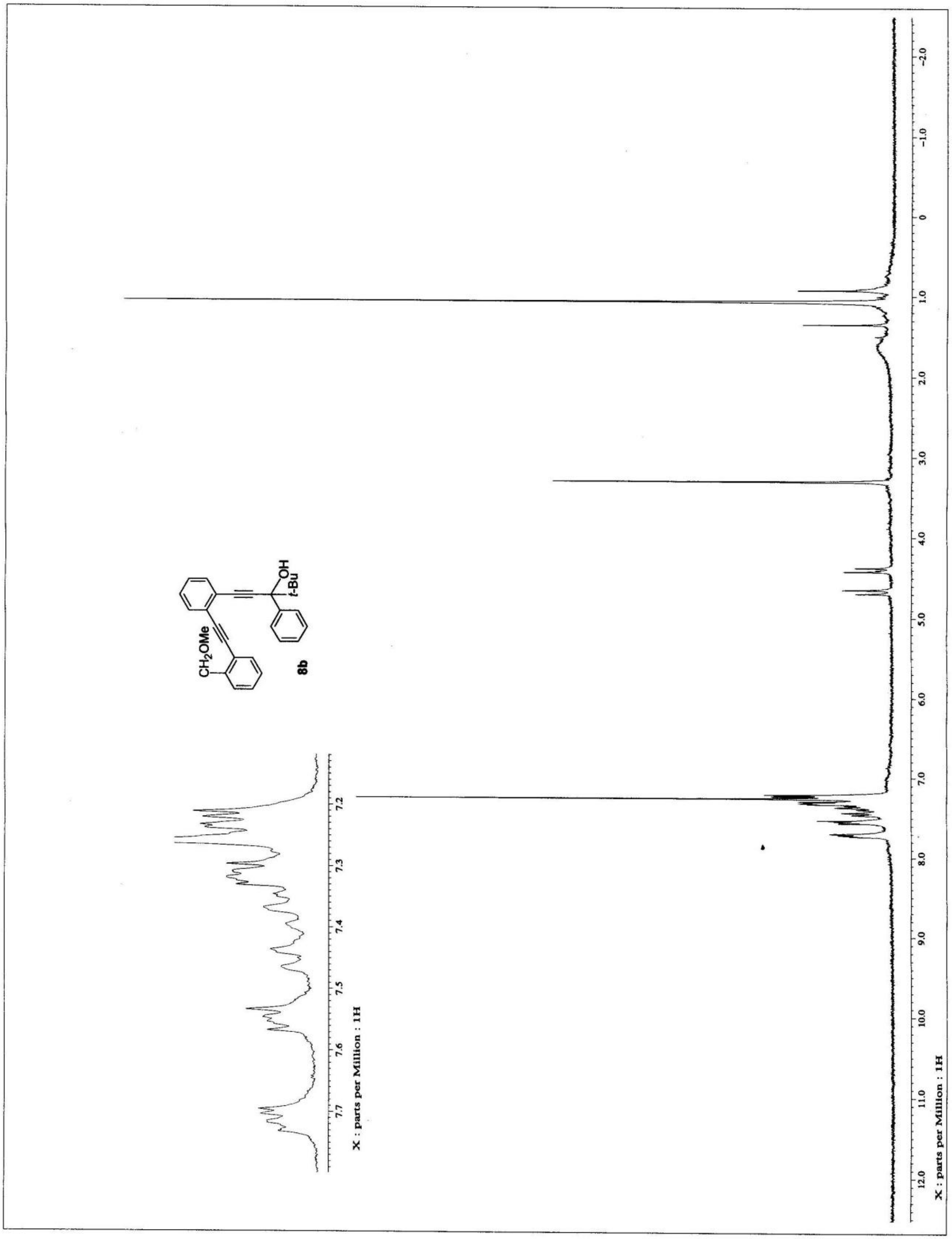
CH2OMe
OH
t-Bu
8b
X : parts per Million : 1H
X : parts per Million : 1H

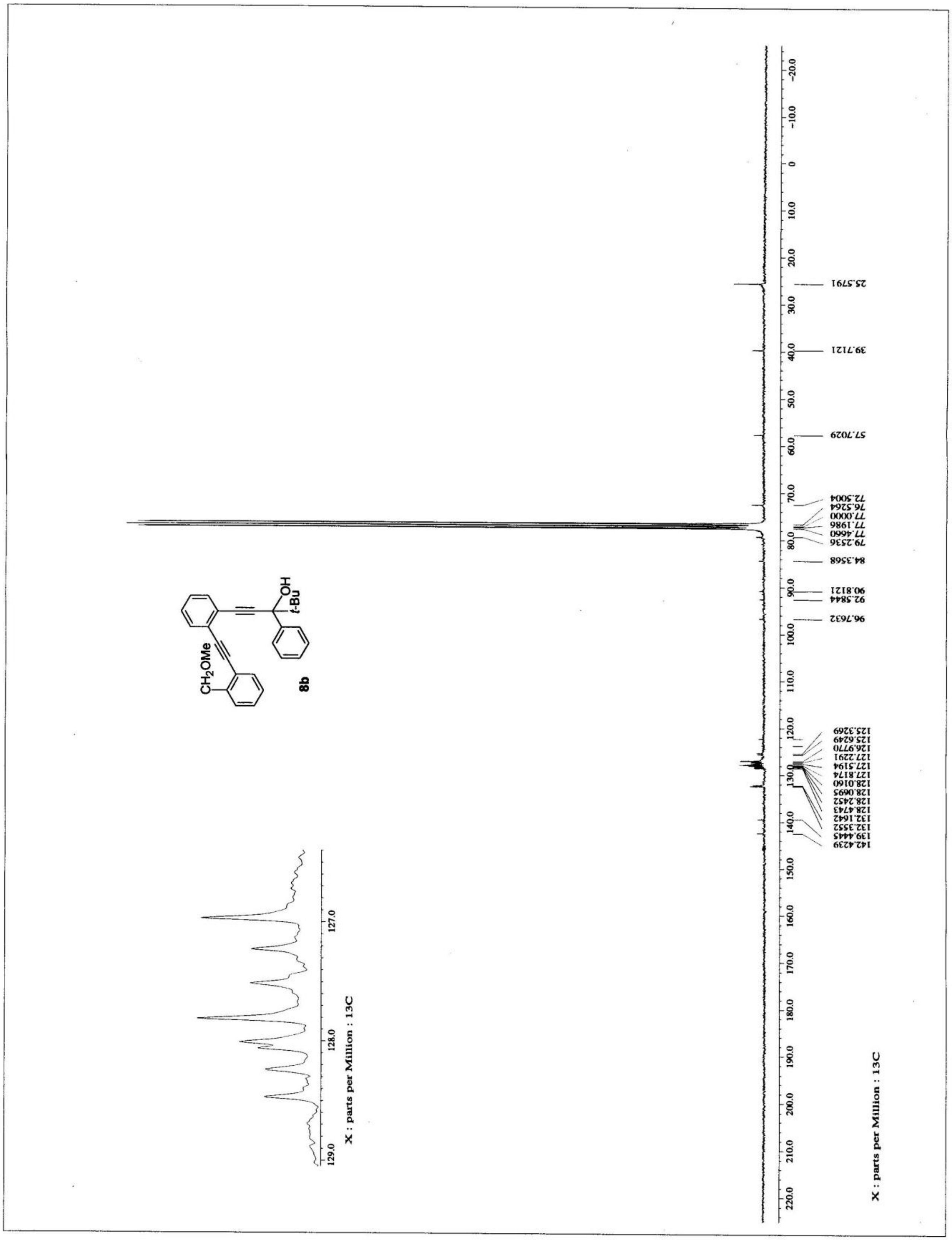
CH2OMe
OH
t-Bu
8b
X : parts per Million : 13C
25.5791
39.7121
57.7029
72.5004
76.5264
77.0000
77.1986
77.4660
79.2536
84.3568
90.8121
92.5844
96.7632
125.3269
125.6249
126.9770
127.2291
127.5194
127.8174
128.0160
128.0695
128.2452
128.4743
132.1642
132.3552
139.4445
142.4239

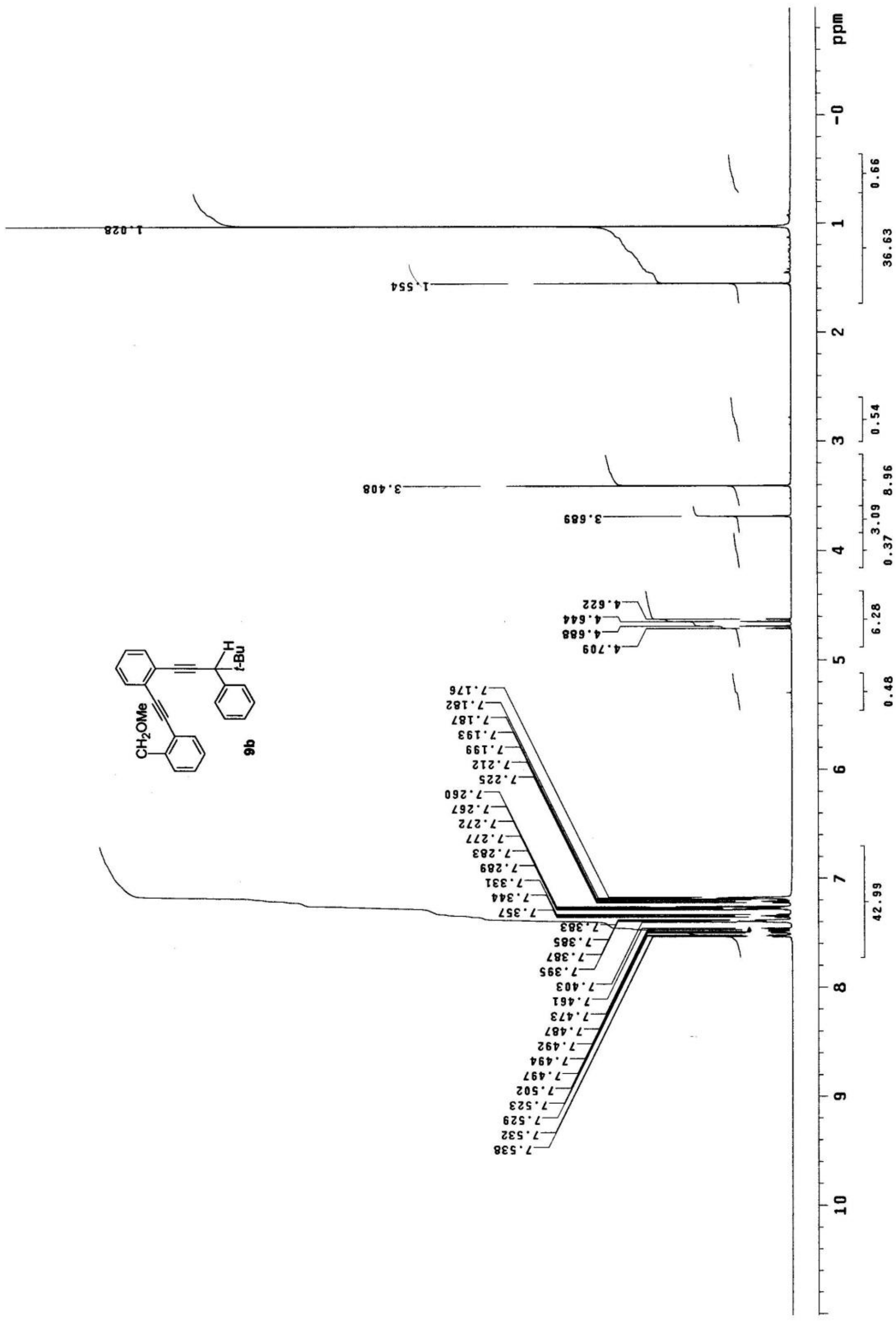
CH2OMe
H
t-Bu
9b
1.028
1.554
3.408
3.689
4.622
4.644
4.688
4.709
7.176
7.182
7.187
7.193
7.199
7.212
7.225
7.260
7.267
7.272
7.277
7.283
7.289
7.331
7.344
7.357
7.383
7.385
7.387
7.395
7.403
7.461
7.473
7.487
7.492
7.494
7.497
7.502
7.523
7.529
7.532
7.538
0.66
36.63
0.54
8.96
3.09
0.37
6.28
0.48
42.99
ppm
0
1
2
3
4
5
6
7
8
9
10

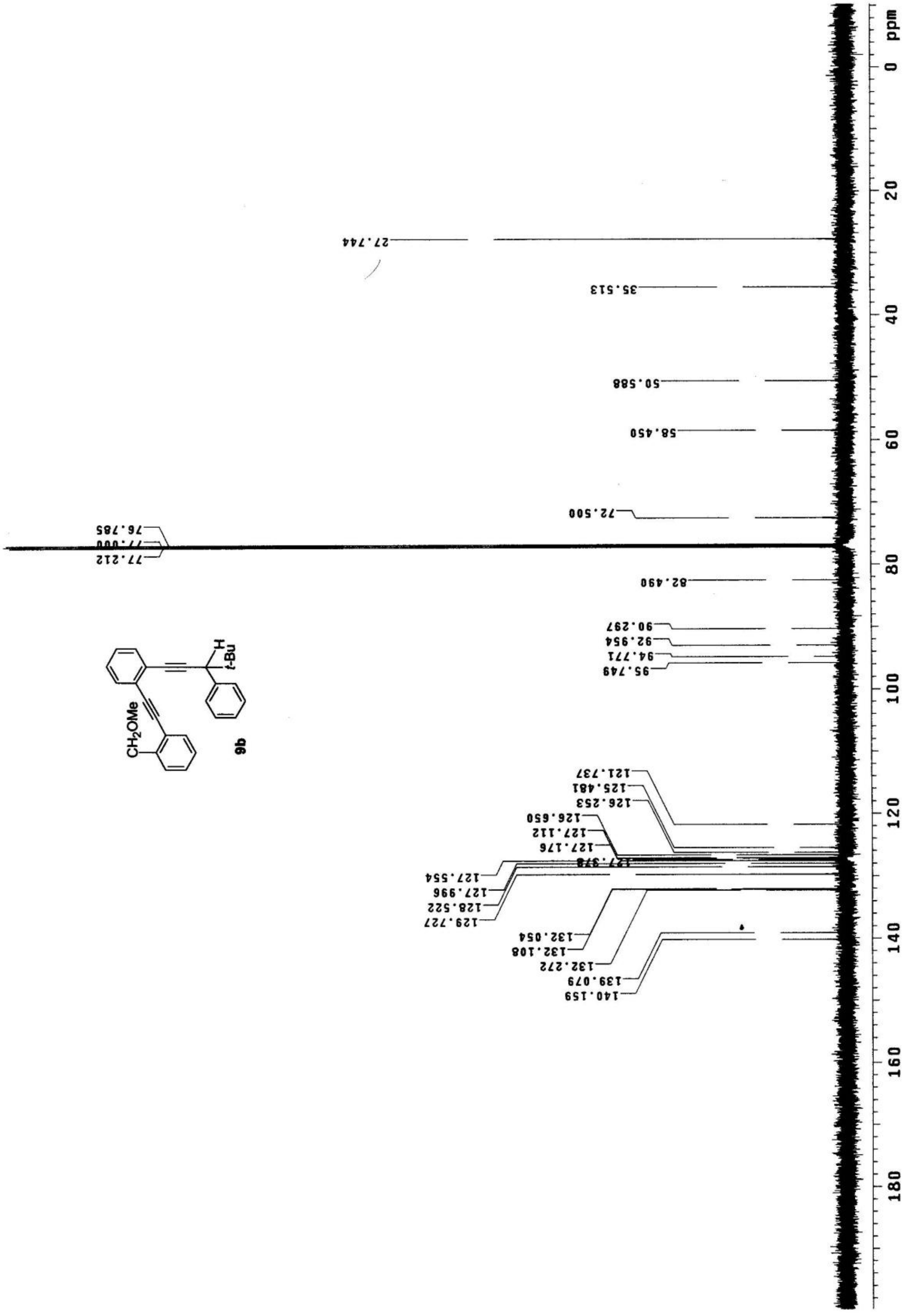

27.744
35.513
50.588
58.450
72.500
76.785
77.000
77.212
82.490
90.297
92.954
94.771
95.749
121.737
125.481
126.253
126.650
127.112
127.176
127.554
127.996
128.522
129.727
132.054
132.108
132.272
139.079
140.159
CH2OMe
H
t-Bu
9b
ppm

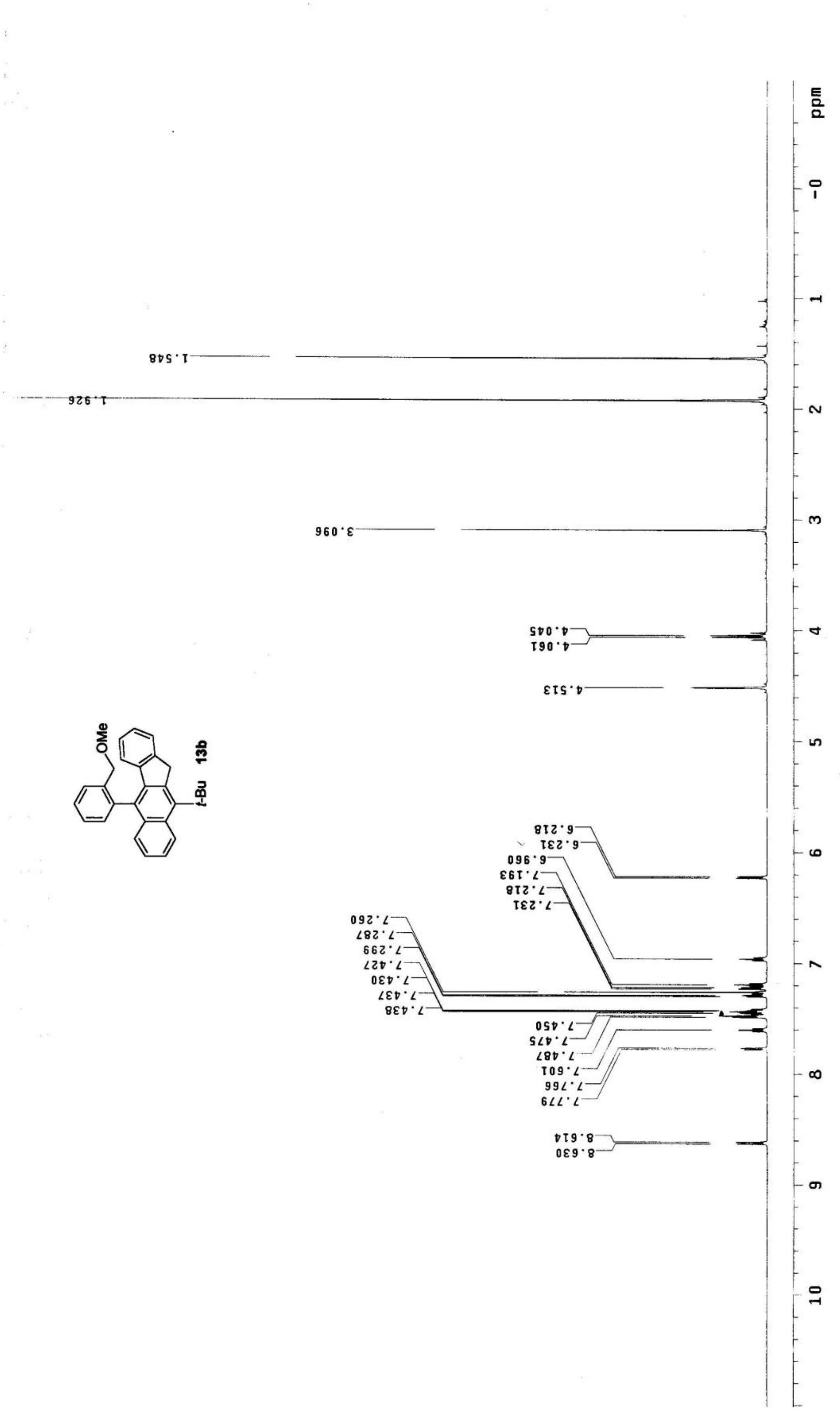
1.548
1.926
3.096
4.045
4.061
4.513
6.218
6.231
6.960
7.193
7.218
7.231
7.260
7.287
7.299
7.427
7.430
7.437
7.438
7.450
7.475
7.487
7.601
7.766
7.779
8.614
8.630
OMe
t-Bu
13b
ppm
0
1
2
3
4
5
6
7
8
9
10

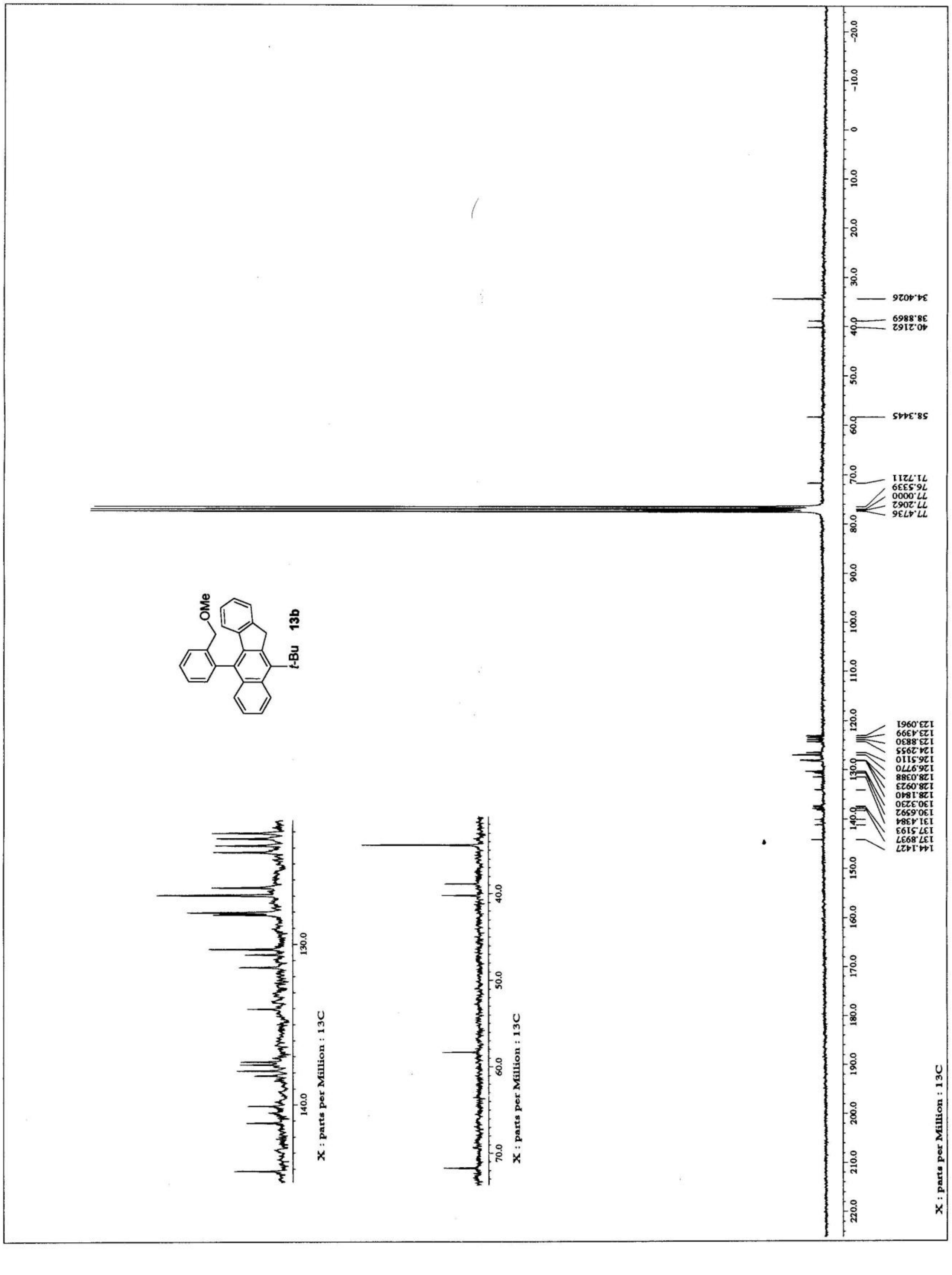
OMe
t-Bu
13b
34.4026
38.8869
40.2162
58.3445
71.7211
76.5339
77.0000
77.2062
77.4736
123.0961
123.4399
123.8830
124.2955
126.5110
126.9770
128.0388
128.0923
128.1840
130.3230
130.6595
131.4384
137.5193
137.8937
144.1427
X : parts per Million : 13C
X : parts per Million : 13C
X : parts per Million : 13C

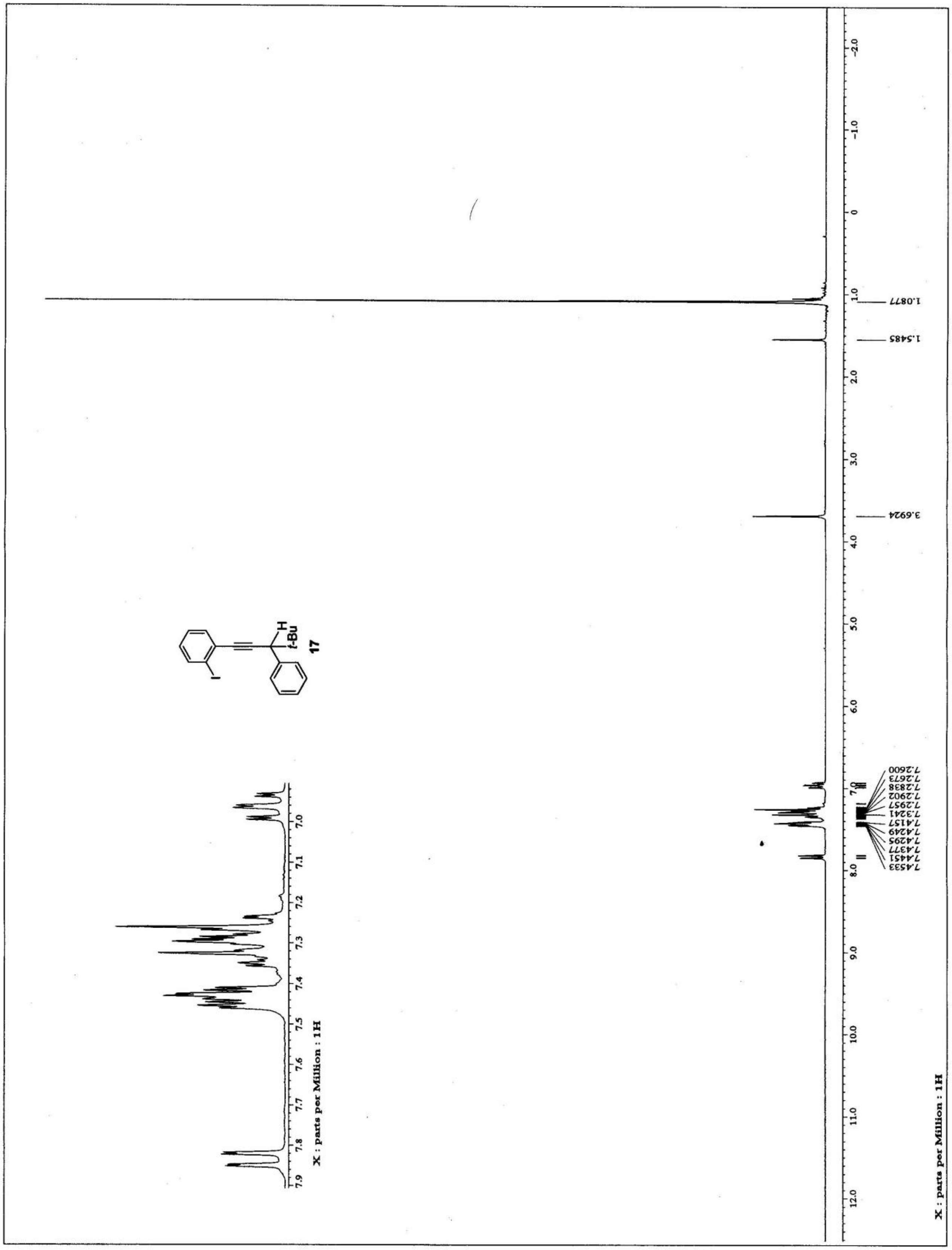

17
t-Bu
H
I
1.0877
1.5485
3.6924
7.2600
7.2673
7.2838
7.2902
7.2957
7.3241
7.4157
7.4249
7.4295
7.4377
7.4451
7.4533
X : parts per Million : 1H
X : parts per Million : 1H

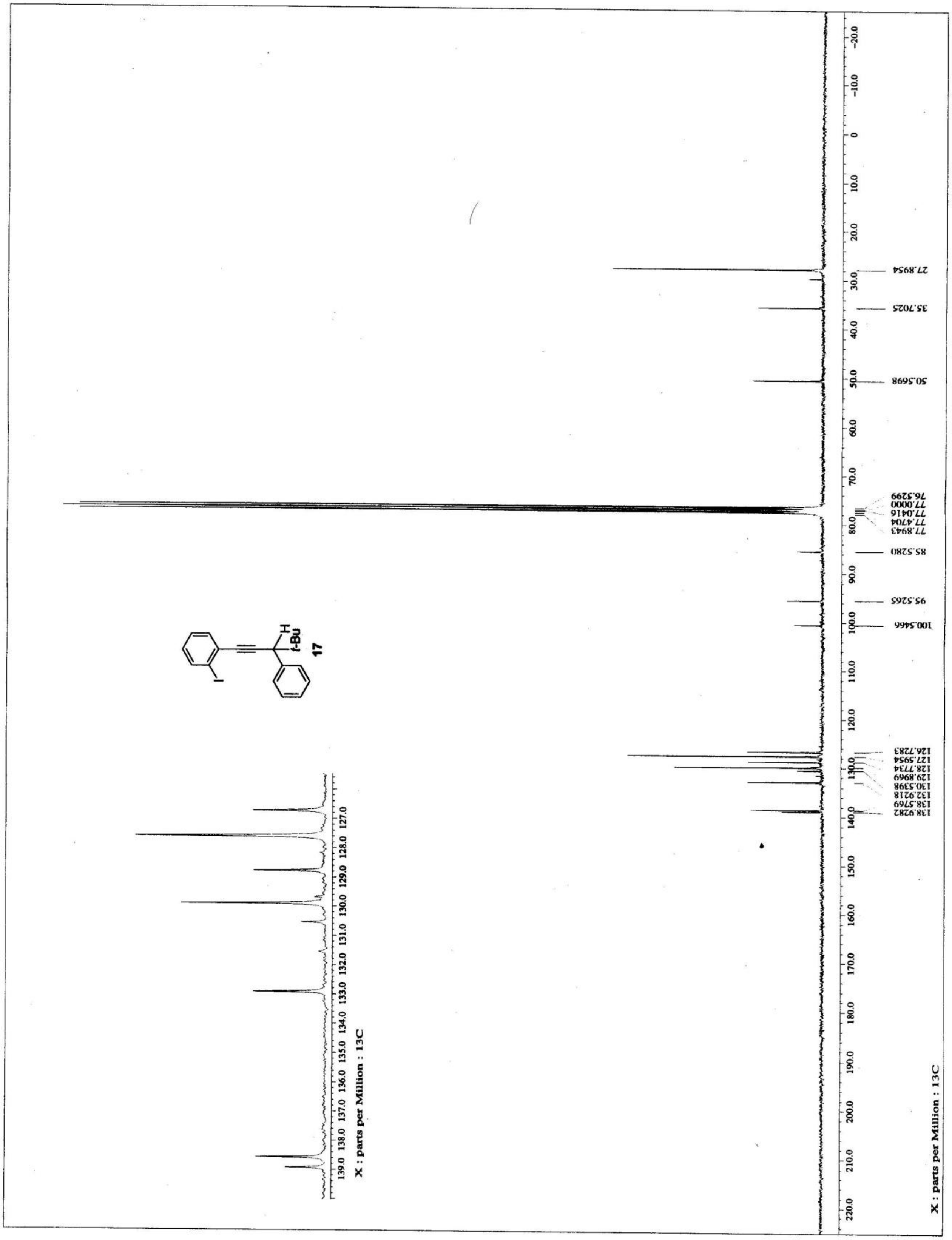
I
H
t-Bu
17
27.8954
35.7025
50.5698
76.5299
77.0000
77.0416
77.4704
77.8943
85.5280
95.5265
100.5466
126.7283
127.5954
128.7734
129.8969
130.5398
132.9218
138.5769
138.9282
X : parts per Million : 13C
X : parts per Million : 13C

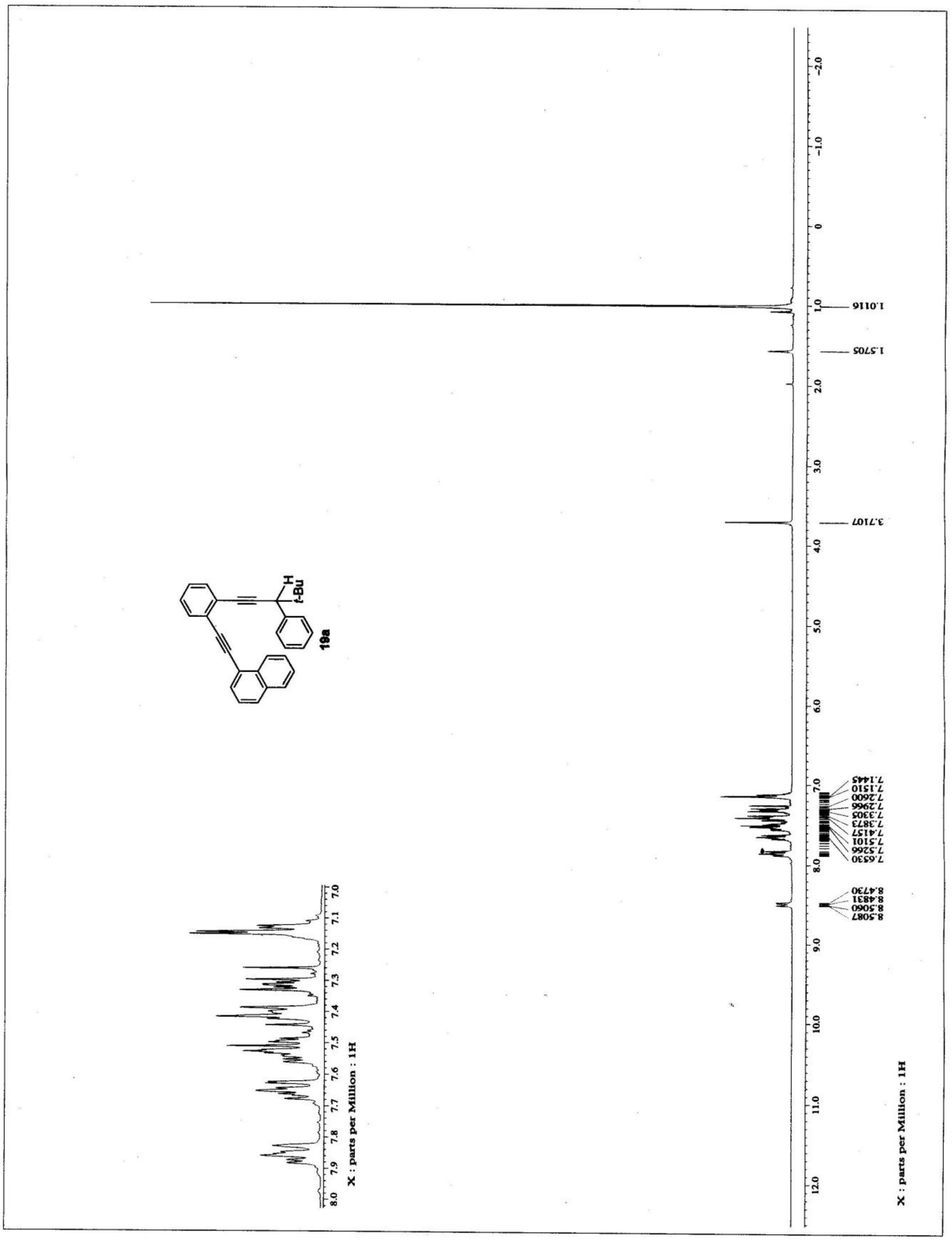

H
t-Bu
19a
1.0116
1.5705
3.7107
7.1445
7.1510
7.2600
7.2966
7.3305
7.3873
7.4157
7.5101
7.5266
7.6530
8.4730
8.4831
8.5060
8.5087
X : parts per Million : 1H
X : parts per Million : 1H

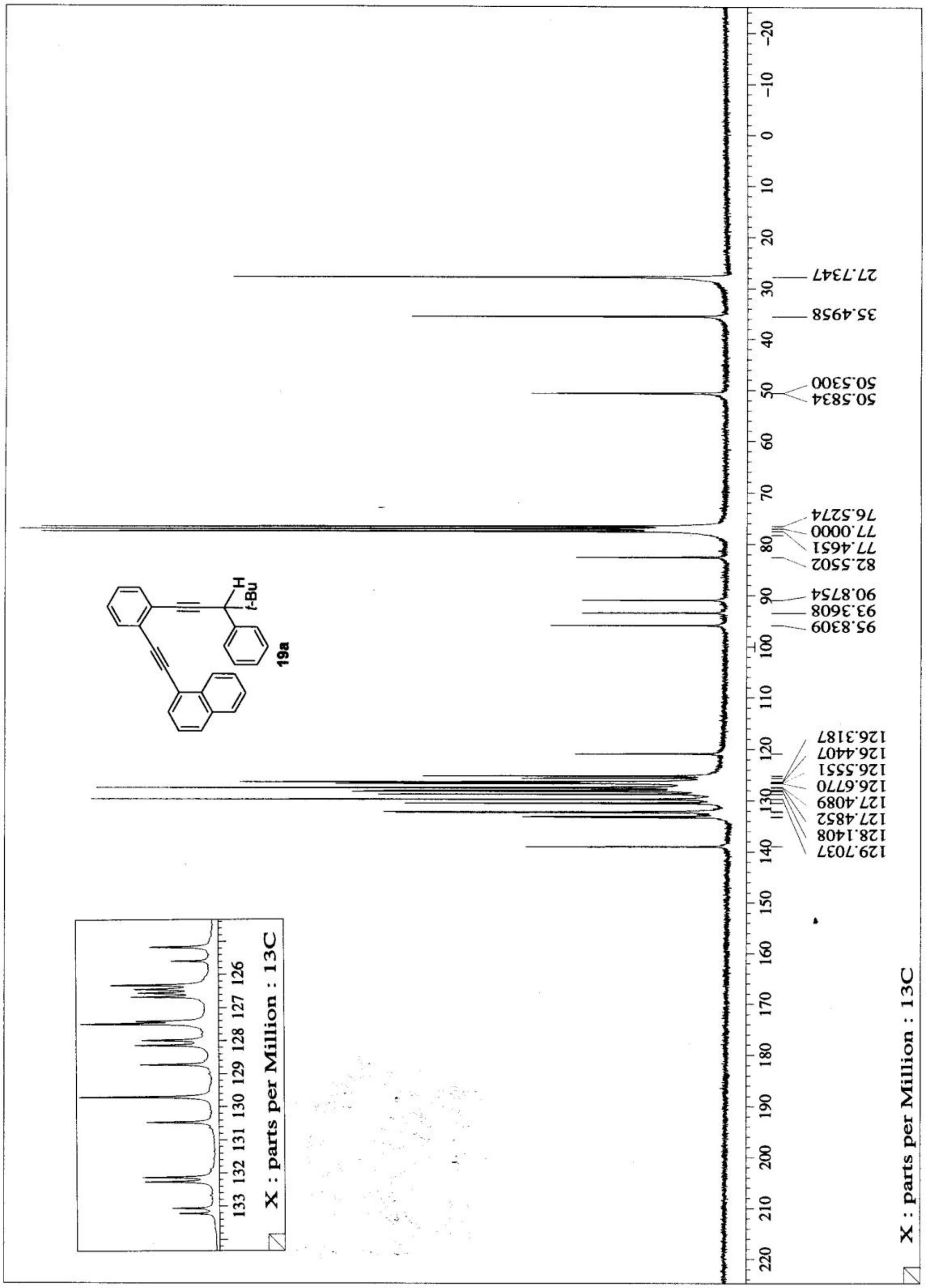
19a
t-Bu
H
27.7347
35.4958
50.5300
50.5834
76.5274
77.0000
77.4651
82.5502
90.8754
93.3608
95.8309
126.3187
126.4407
126.5551
126.6770
127.4089
127.4852
128.1408
129.7037
X : parts per Million : 13C
133 132 131 130 129 128 127 126
X : parts per Million : 13C

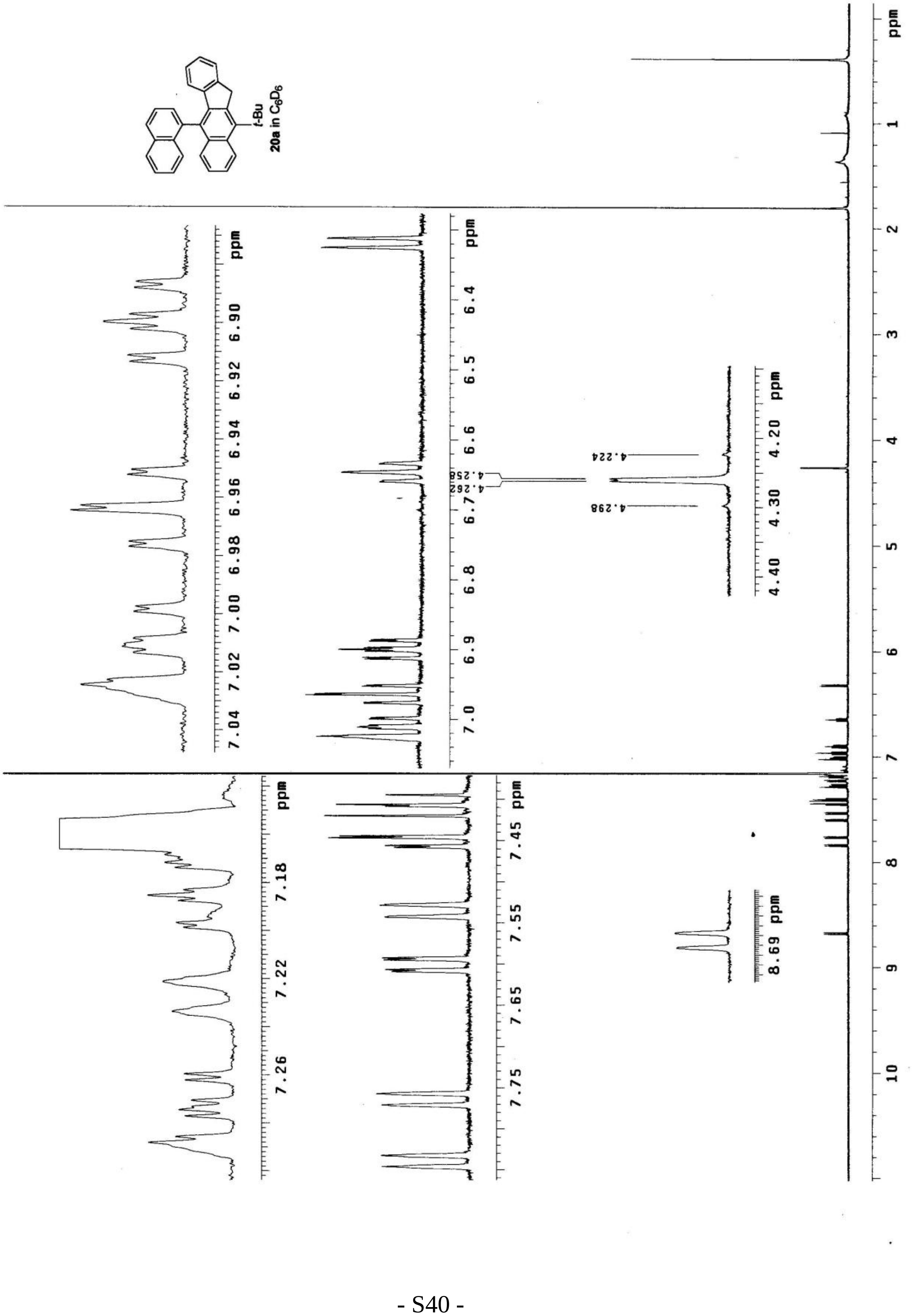
t-Bu
20a in C_6D_6

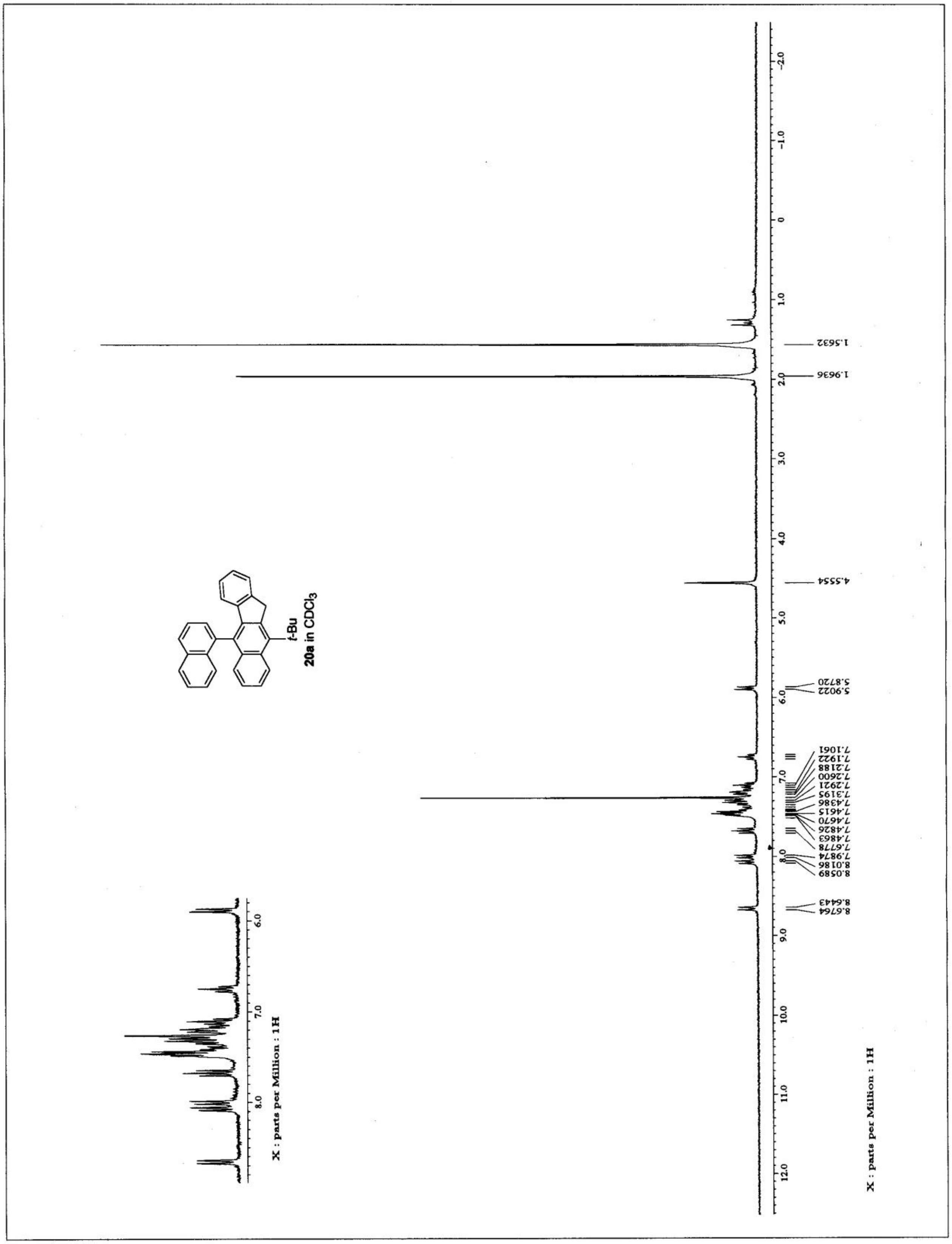
t-Bu
20a in CDCl3
1.5632
1.9636
4.5554
5.8720
5.9022
7.1061
7.1922
7.2188
7.2600
7.2921
7.3195
7.4386
7.4615
7.4670
7.4826
7.4863
7.6778
7.9874
8.0186
8.0589
8.6443
8.6764
X : parts per Million : 1H

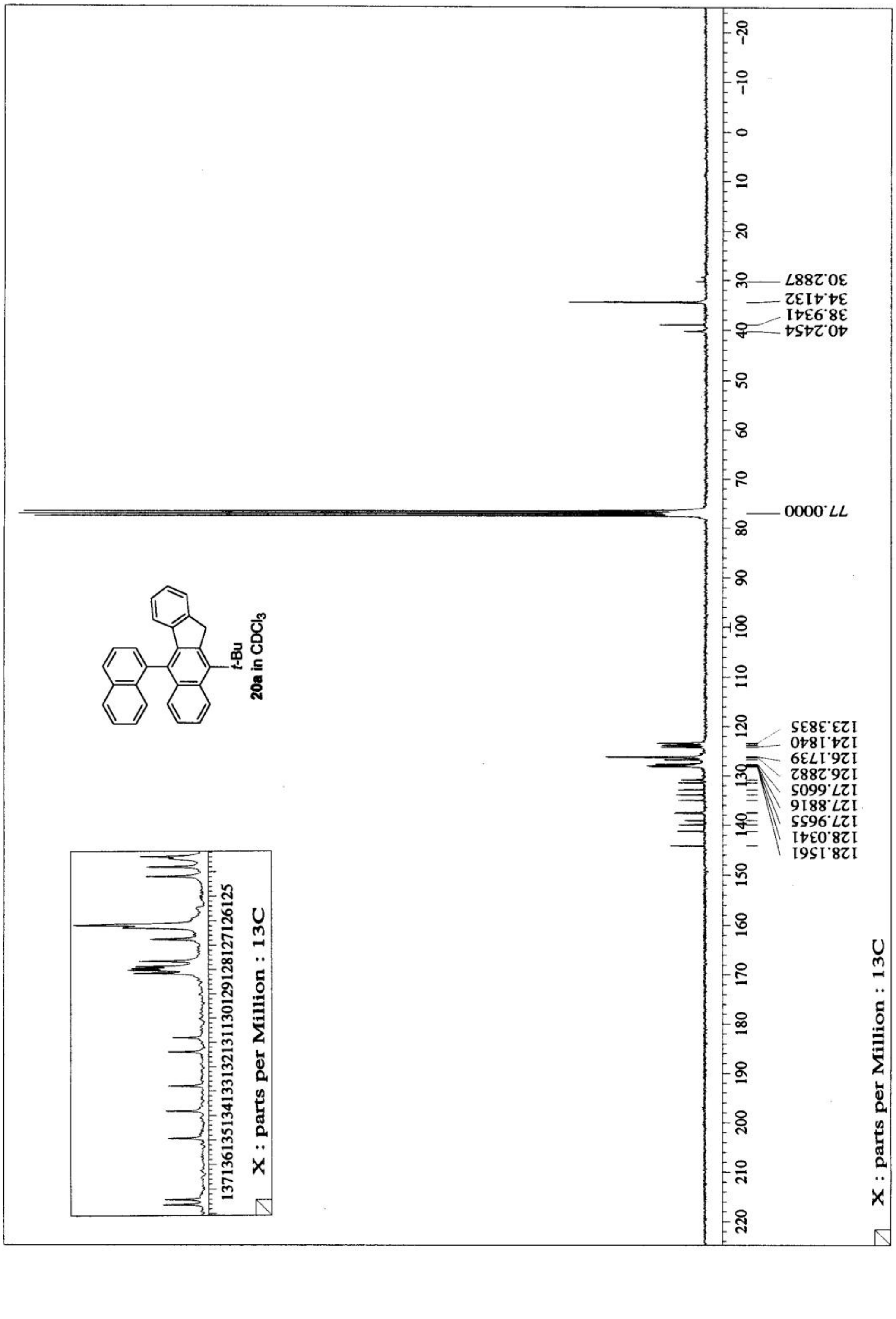

t-Bu
20a in CDCl3
30.2887
34.4132
38.9341
40.2454
77.0000
123.3835
124.1840
126.1739
126.2882
127.6605
127.8816
127.9655
128.0341
128.1561
X : parts per Million : 13C
X : parts per Million : 13C

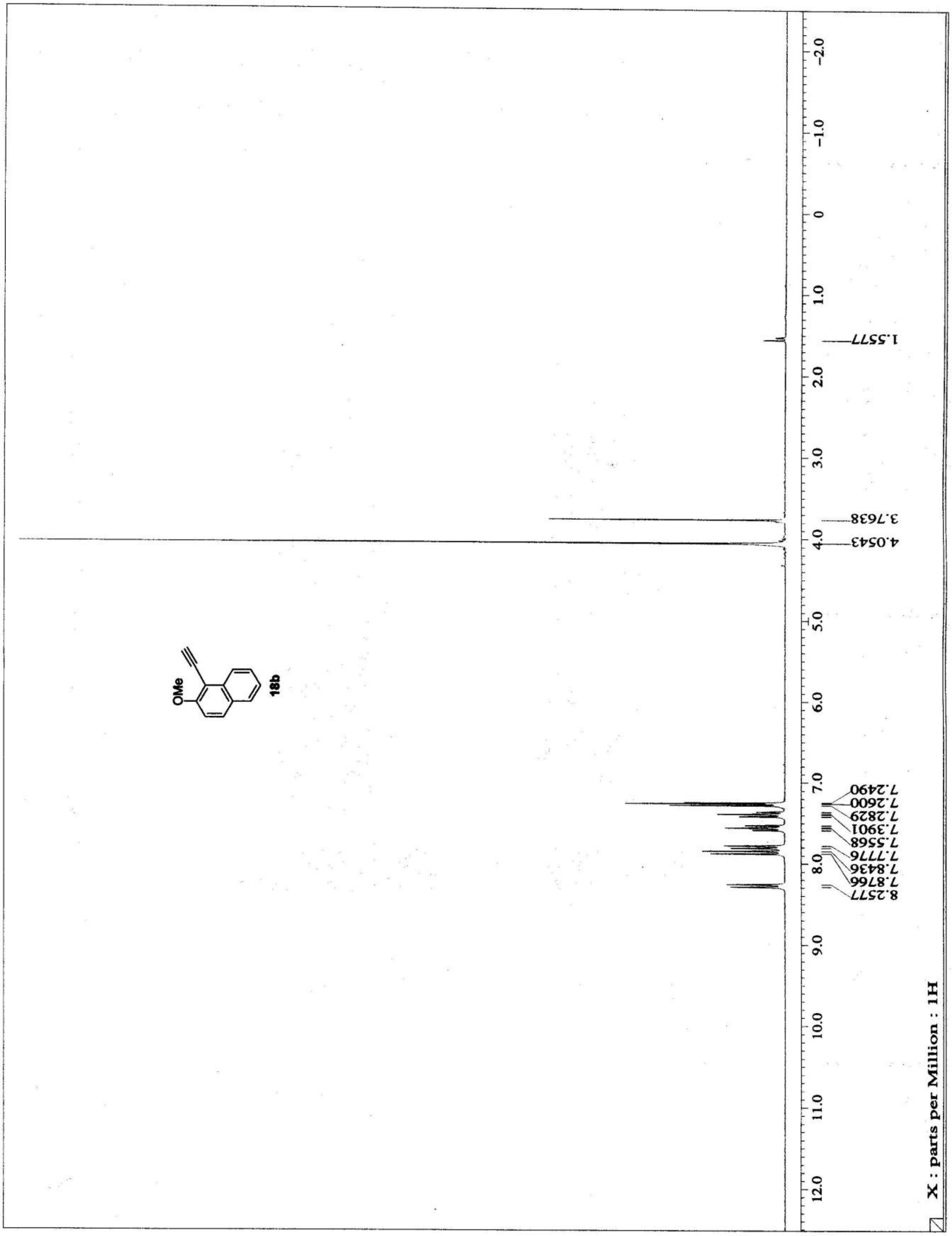
OMe
18b
1.5577
3.7638
4.0543
7.2490
7.2600
7.2829
7.3901
7.5568
7.7776
7.8436
7.8766
8.2577
X : parts per Million : 1H

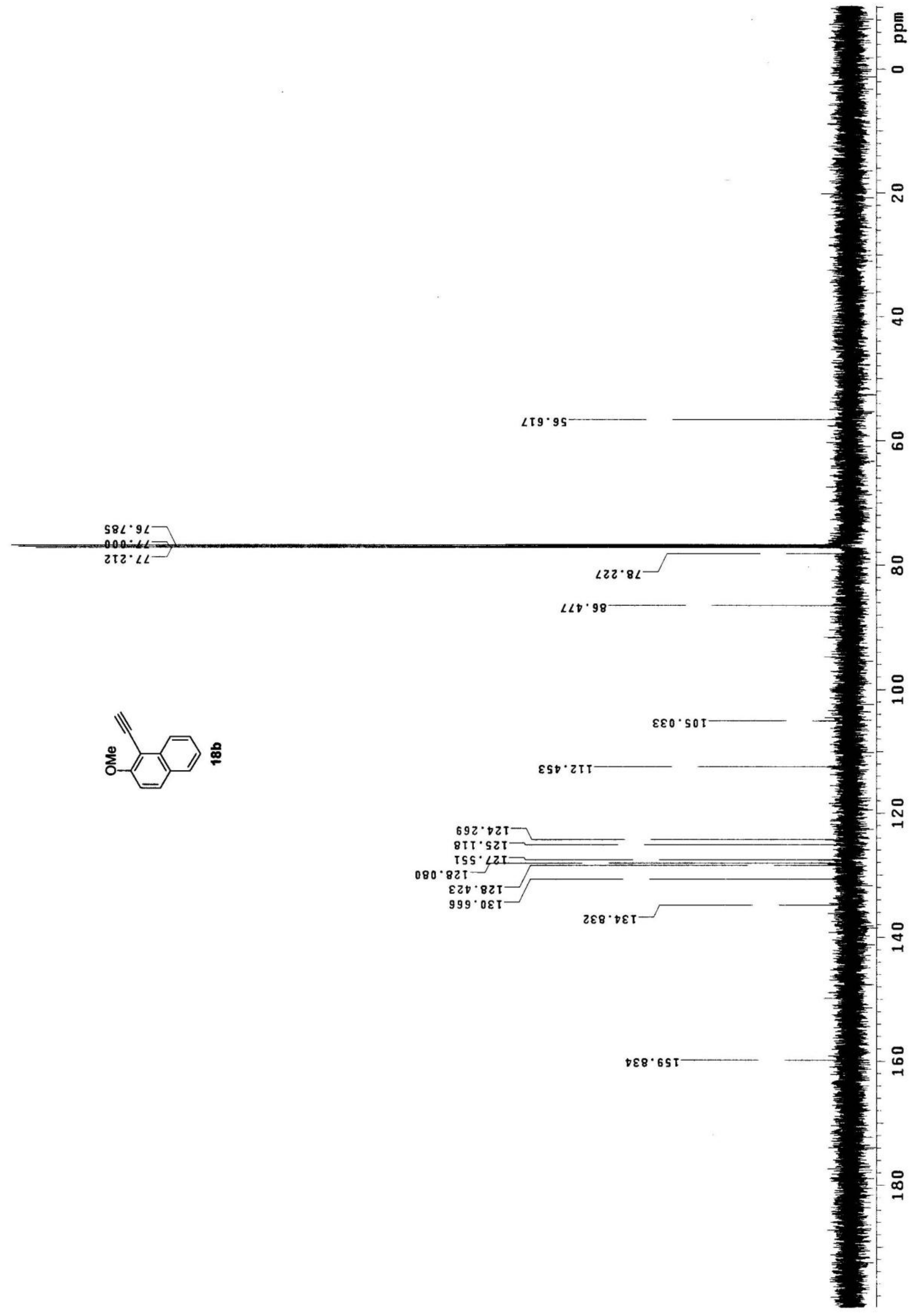

OMe
18b
159.834
134.832
130.666
128.423
128.080
127.551
125.118
124.269
112.453
105.033
86.477
78.227
77.212
77.000
76.785
56.617
ppm

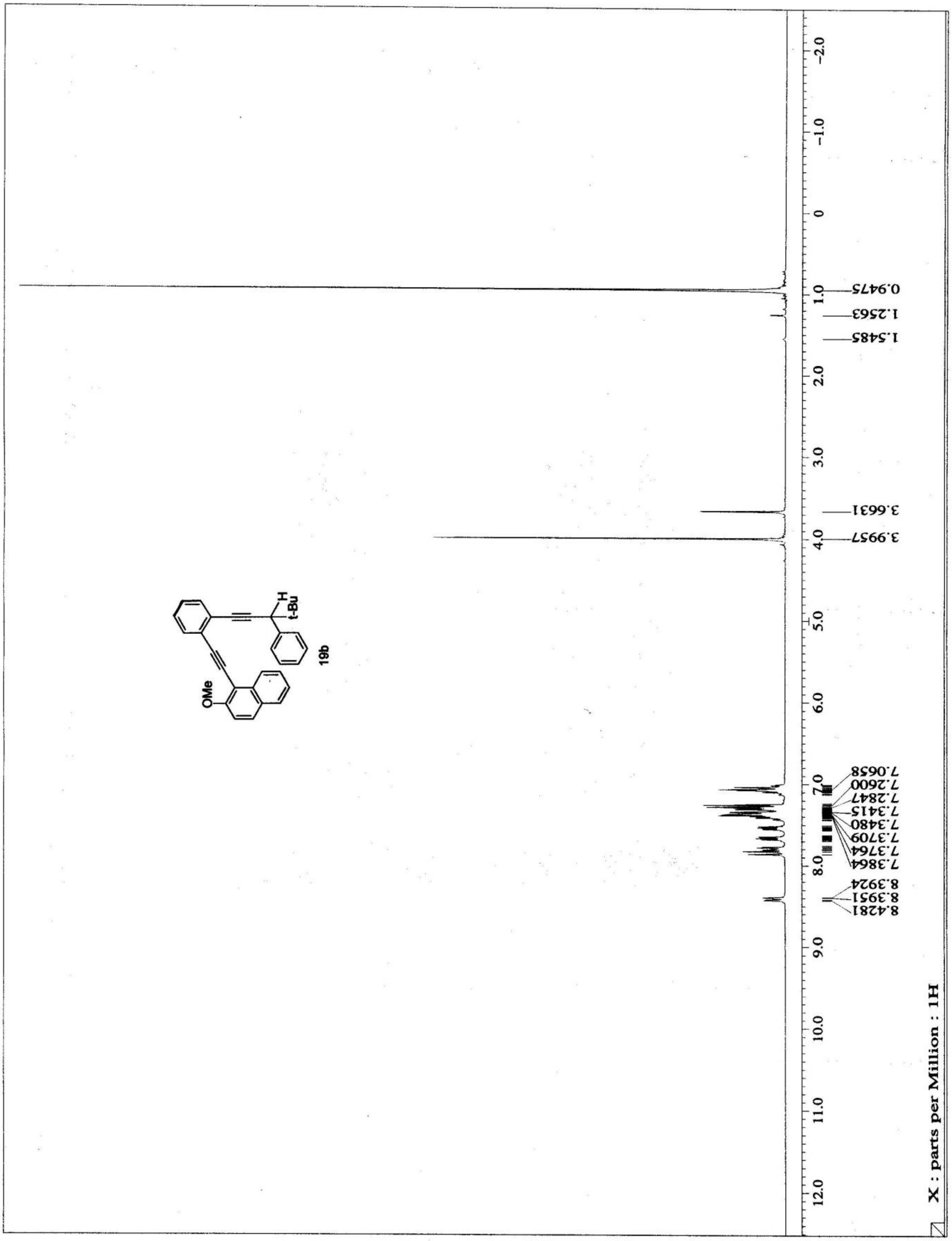
OMe
H
t-Bu
19b
0.9475
1.2563
1.5485
3.6631
3.9957
7.0658
7.2600
7.2847
7.3415
7.3480
7.3709
7.3764
7.3864
8.3924
8.3951
8.4281
-2.0
-1.0
0
1.0
2.0
3.0
4.0
5.0
6.0
7.0
8.0
9.0
10.0
11.0
12.0
X : parts per Million : 1H

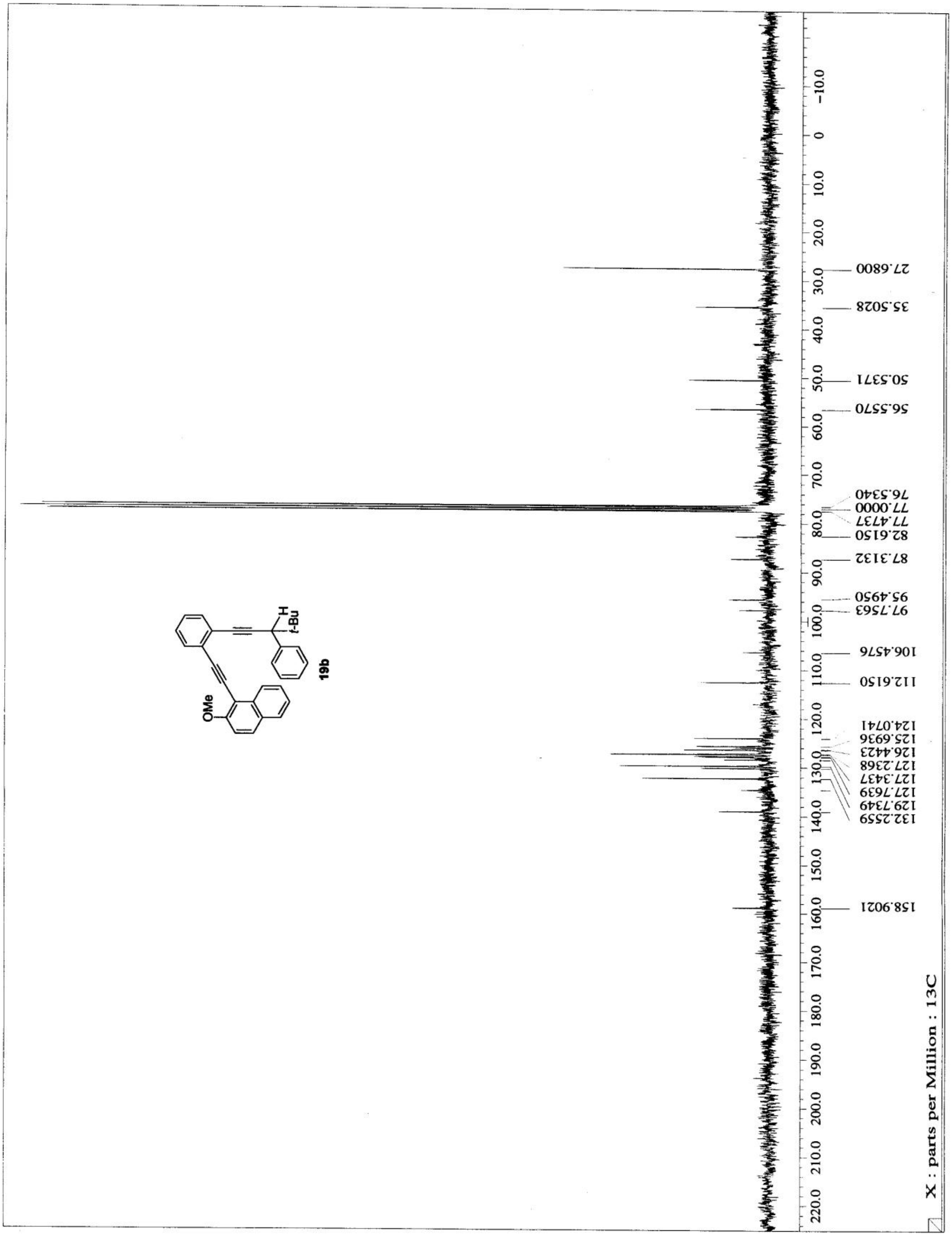

OMe
H
t-Bu
19b
27.6800
35.5028
50.5371
56.5570
76.5340
77.0000
77.4737
82.6150
87.3132
95.4950
97.7563
106.4576
112.6150
124.0741
125.6936
126.4423
127.2368
127.3437
127.7639
129.7349
132.2559
158.9021
X : parts per Million : 13C

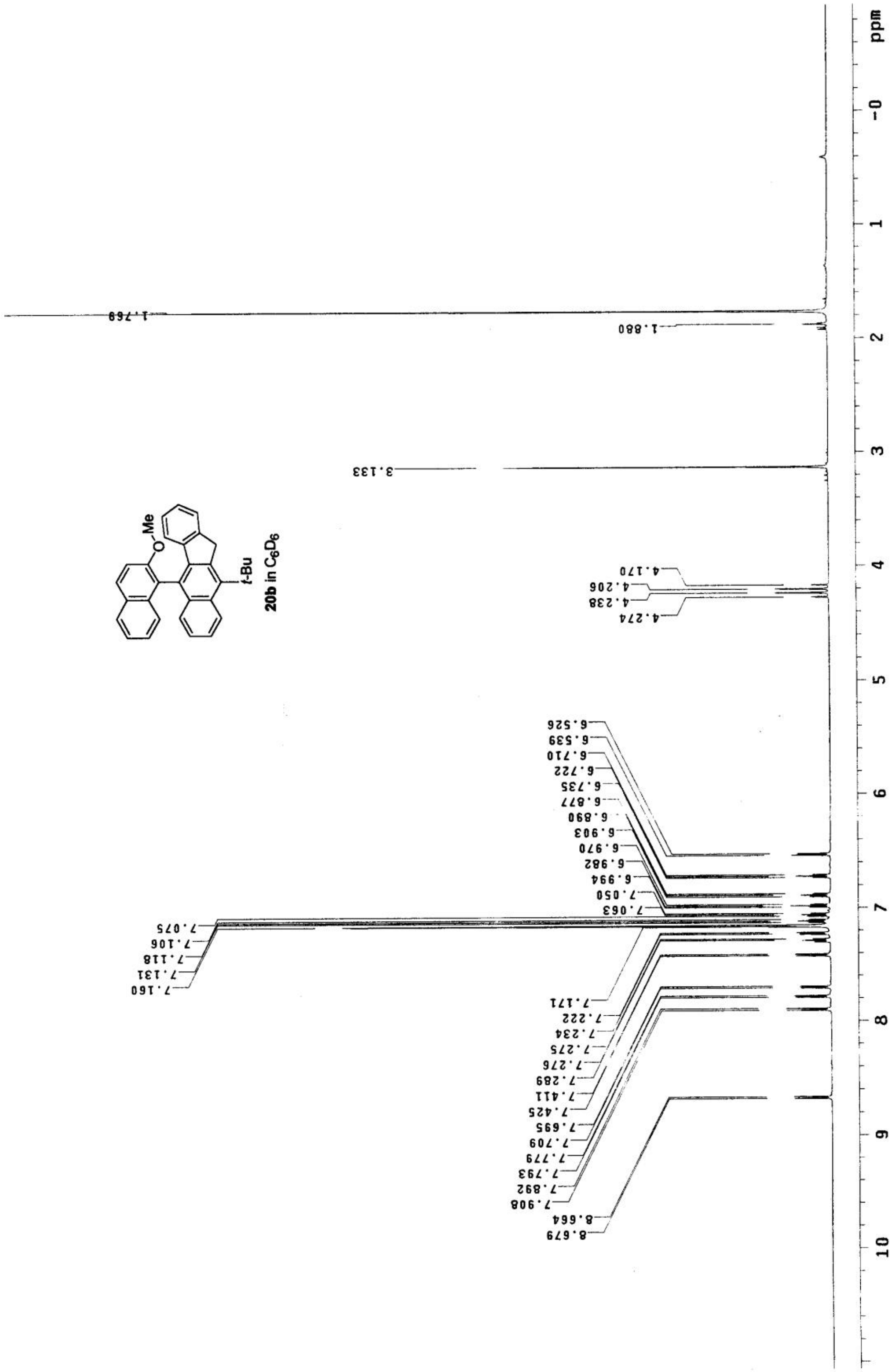
Me
O
t-Bu
20b in C$_6$D$_6$
1.769
1.880
3.133
4.170
4.206
4.238
4.274
6.526
6.539
6.710
6.722
6.735
6.877
6.890
6.903
6.970
6.982
6.994
7.050
7.063
7.075
7.106
7.118
7.131
7.160
7.171
7.222
7.234
7.275
7.276
7.289
7.411
7.425
7.695
7.709
7.779
7.793
7.892
7.908
8.664
8.679
ppm
-0
1
2
3
4
5
6
7
8
9
10

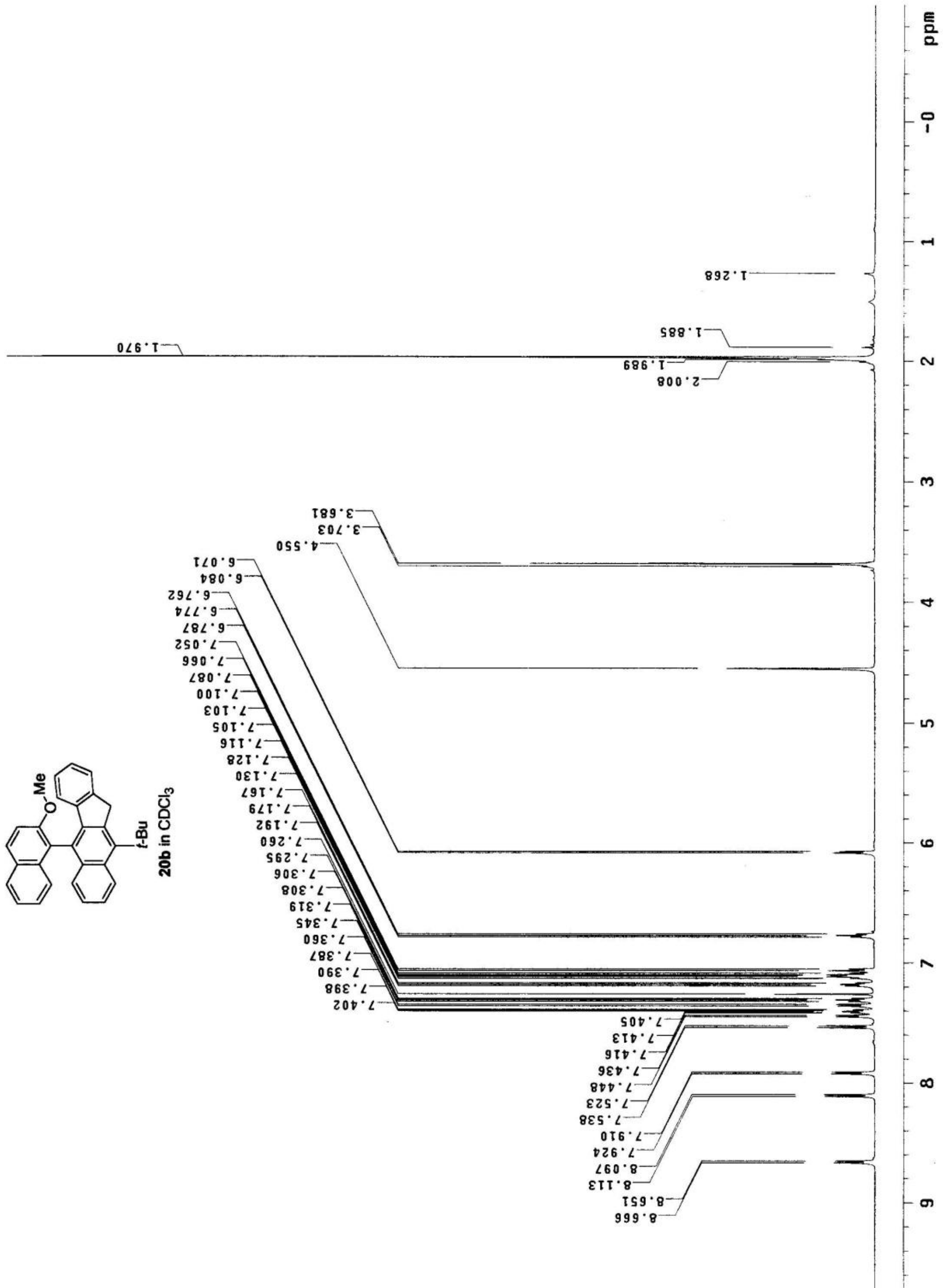
Me
t-Bu
20b in CDCl3
1.268
1.885
1.970
1.989
2.008
3.681
3.703
4.550
6.071
6.084
6.762
6.774
6.787
7.052
7.066
7.087
7.100
7.103
7.105
7.116
7.128
7.130
7.167
7.179
7.192
7.260
7.295
7.306
7.308
7.319
7.345
7.360
7.387
7.390
7.398
7.402
7.405
7.413
7.416
7.436
7.448
7.523
7.538
7.910
7.924
8.097
8.113
8.651
8.666
ppm

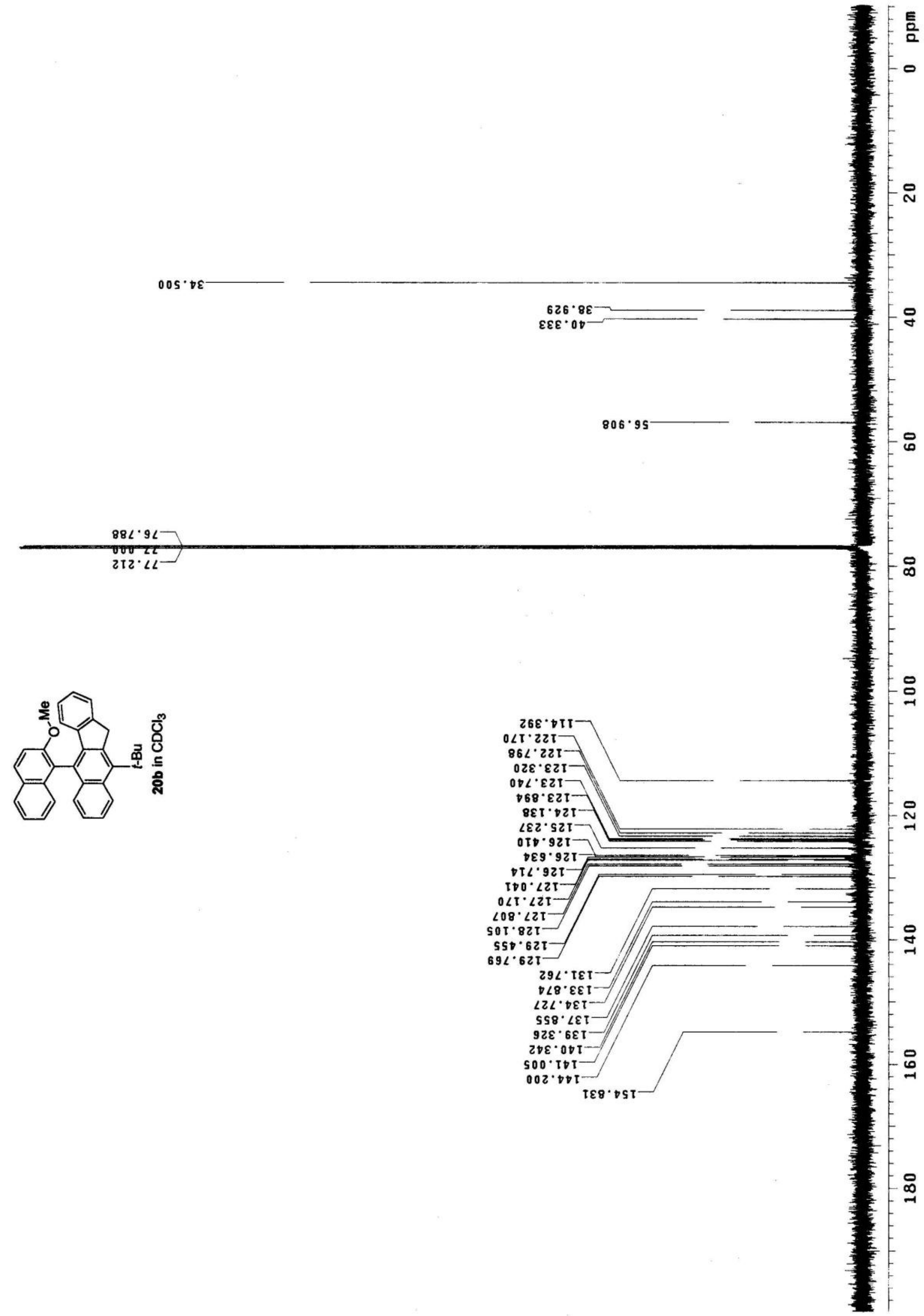

Me
t-Bu
20b in CDCl$_3$
34.500
38.929
40.333
56.908
76.788
77.000
77.212
114.392
122.170
122.798
123.320
123.740
123.894
124.138
125.237
126.410
126.634
126.714
127.041
127.170
127.807
128.105
129.455
129.769
131.762
133.874
134.727
137.855
139.326
140.342
141.005
144.200
154.831
ppm
0
20
40
60
80
100
120
140
160
180

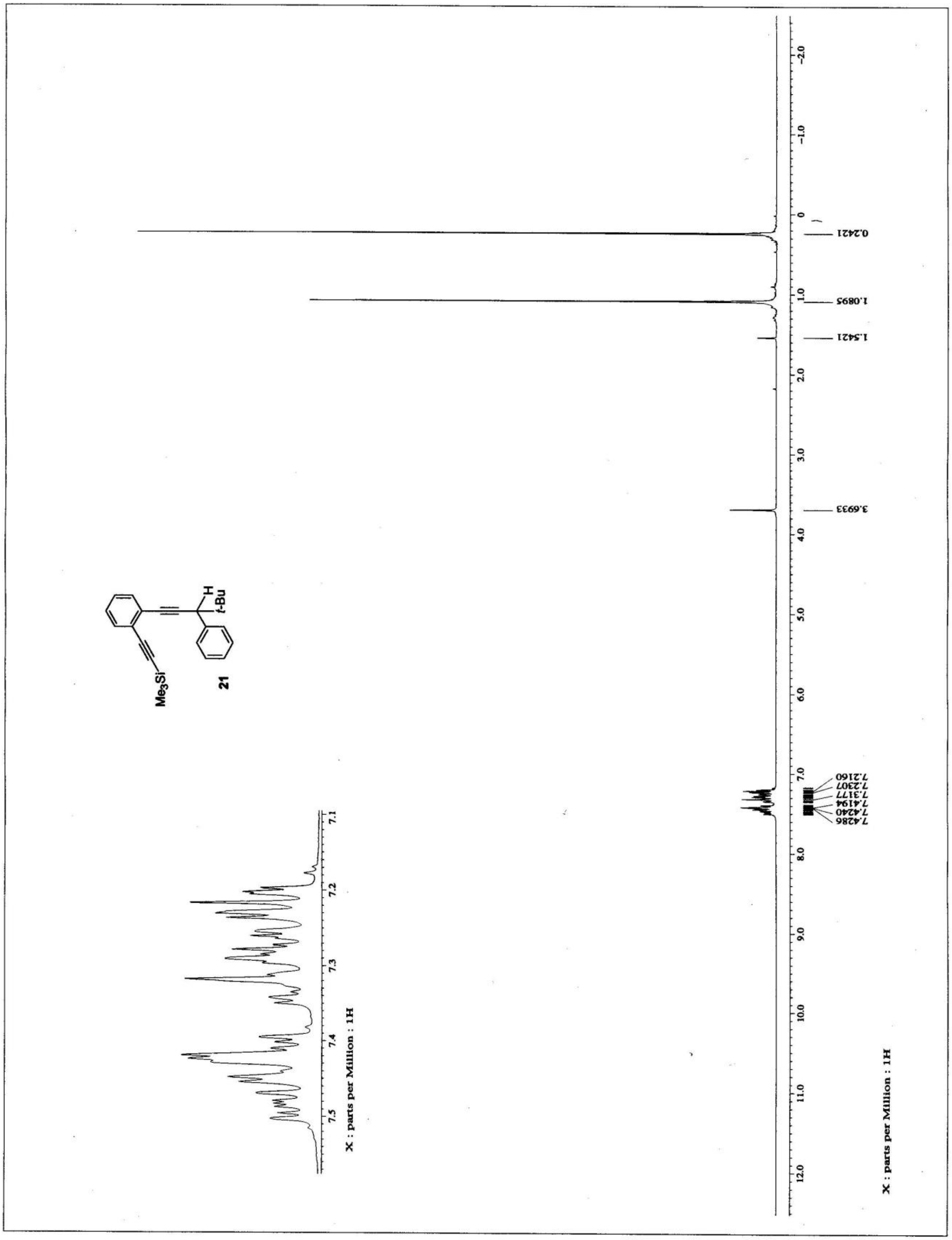
Me3Si
t-Bu
H
21
0.2421
1.0895
1.5421
3.6933
7.2160
7.2307
7.3177
7.4194
7.4240
7.4286
X : parts per Million : 1H
X : parts per Million : 1H

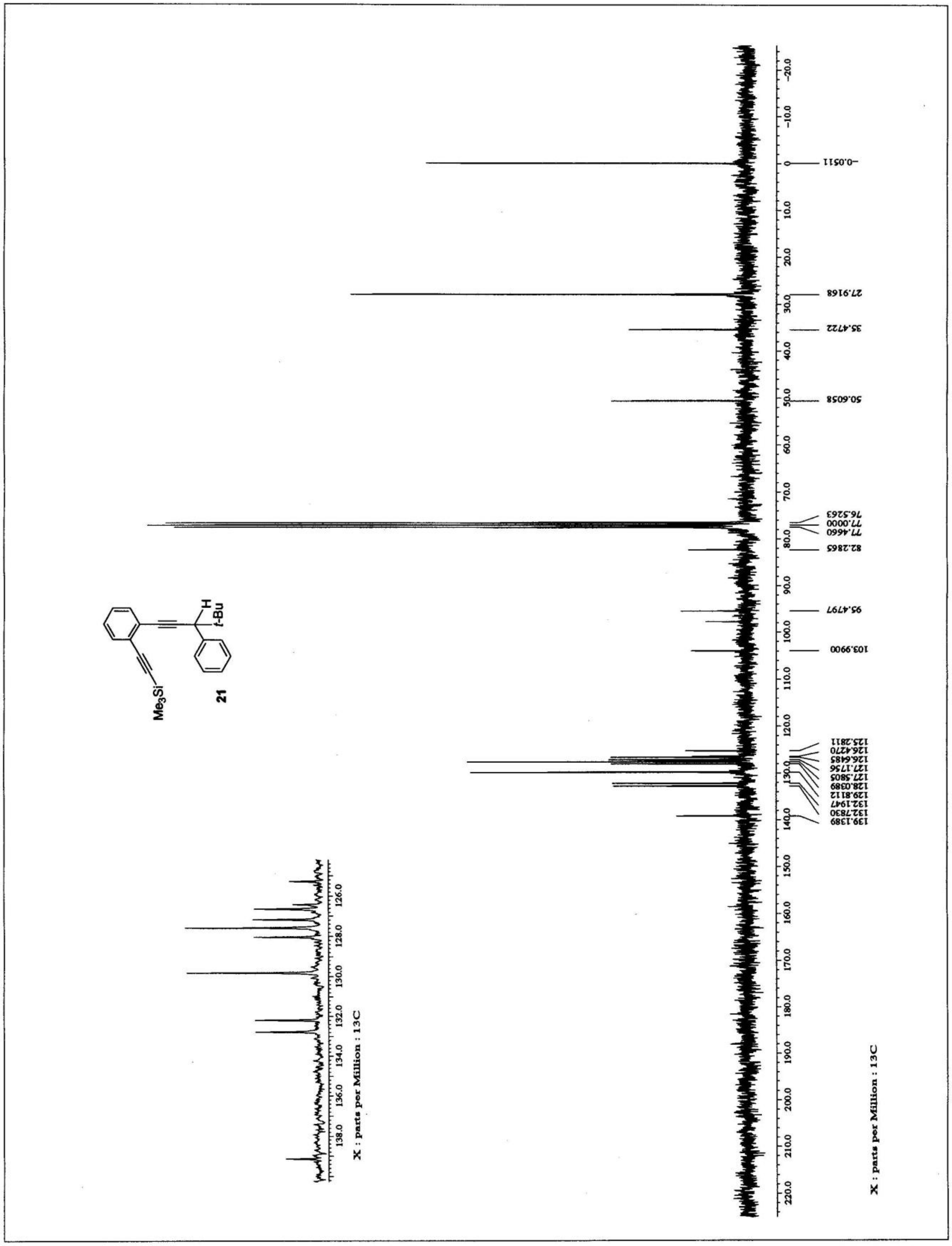
Me3Si
H
t-Bu
21
-0.0511
27.9168
35.4722
50.6058
76.5263
77.0000
77.4660
82.2865
95.4797
103.9900
125.2811
126.4270
126.6485
127.1756
127.5805
128.0389
128.8112
132.1947
132.7830
139.1389
X : parts per Million : 13C
X : parts per Million : 13C

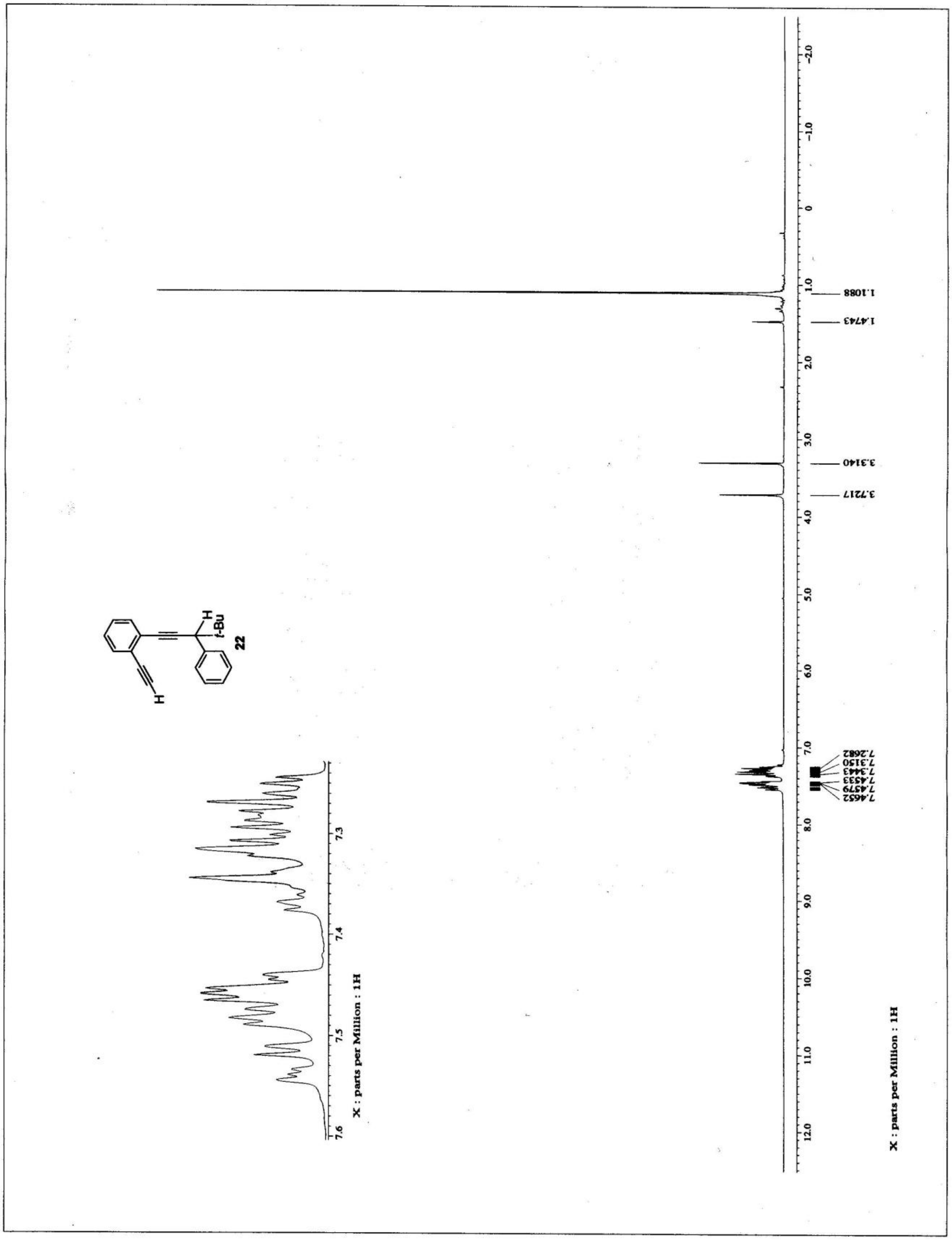
H
t-Bu
22
1.1088
1.4743
3.3140
3.7217
7.2682
7.3150
7.3443
7.4533
7.4579
7.4652
X : parts per Million : 1H
X : parts per Million : 1H

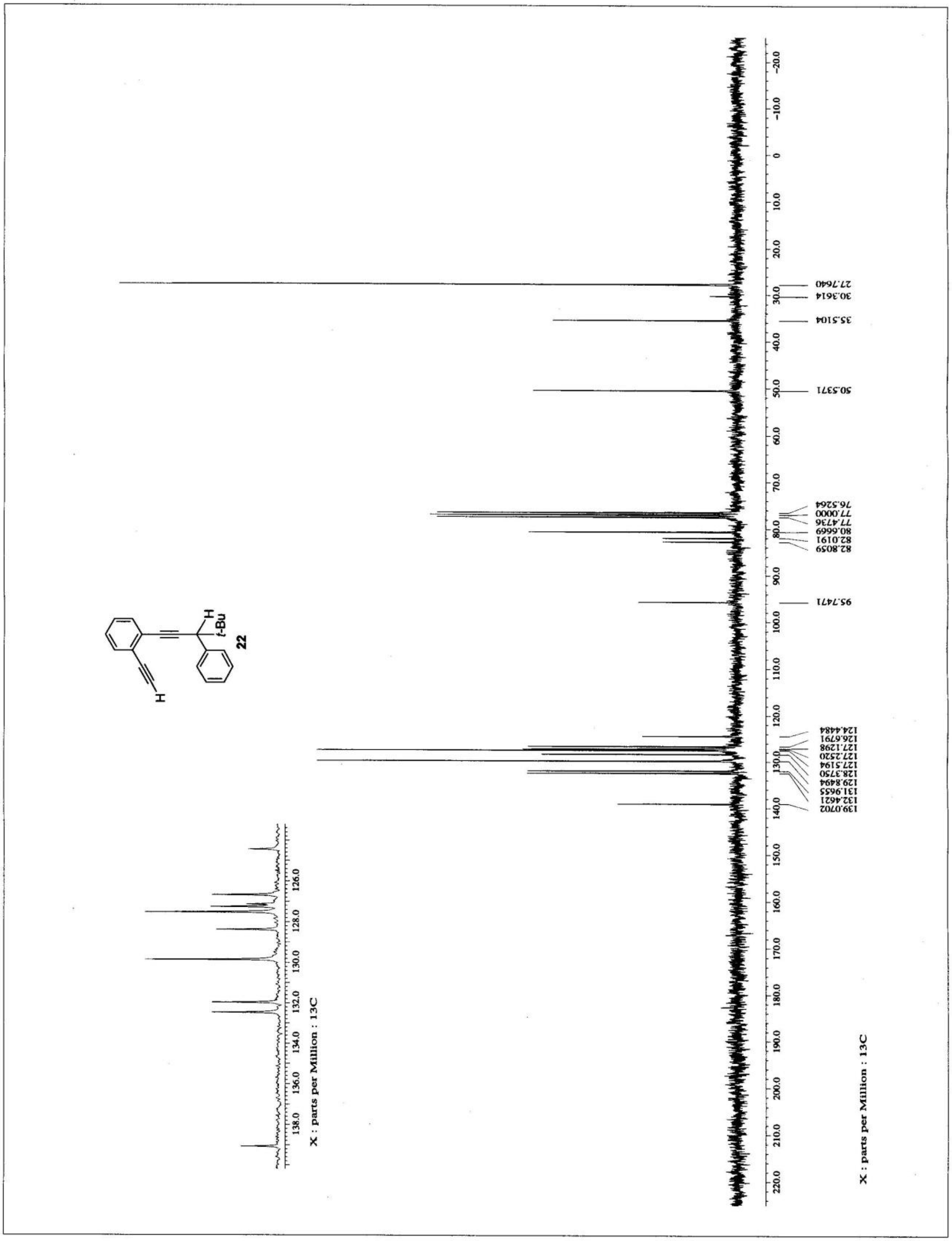

H
t-Bu
22
27.7640
30.3614
35.5104
50.5371
76.5264
77.0000
77.4736
80.6669
82.0191
82.8059
95.7471
124.4484
126.6791
127.1298
127.2520
127.5194
128.3750
129.8494
131.9655
132.4621
139.0702
X : parts per Million : 13C
X : parts per Million : 13C

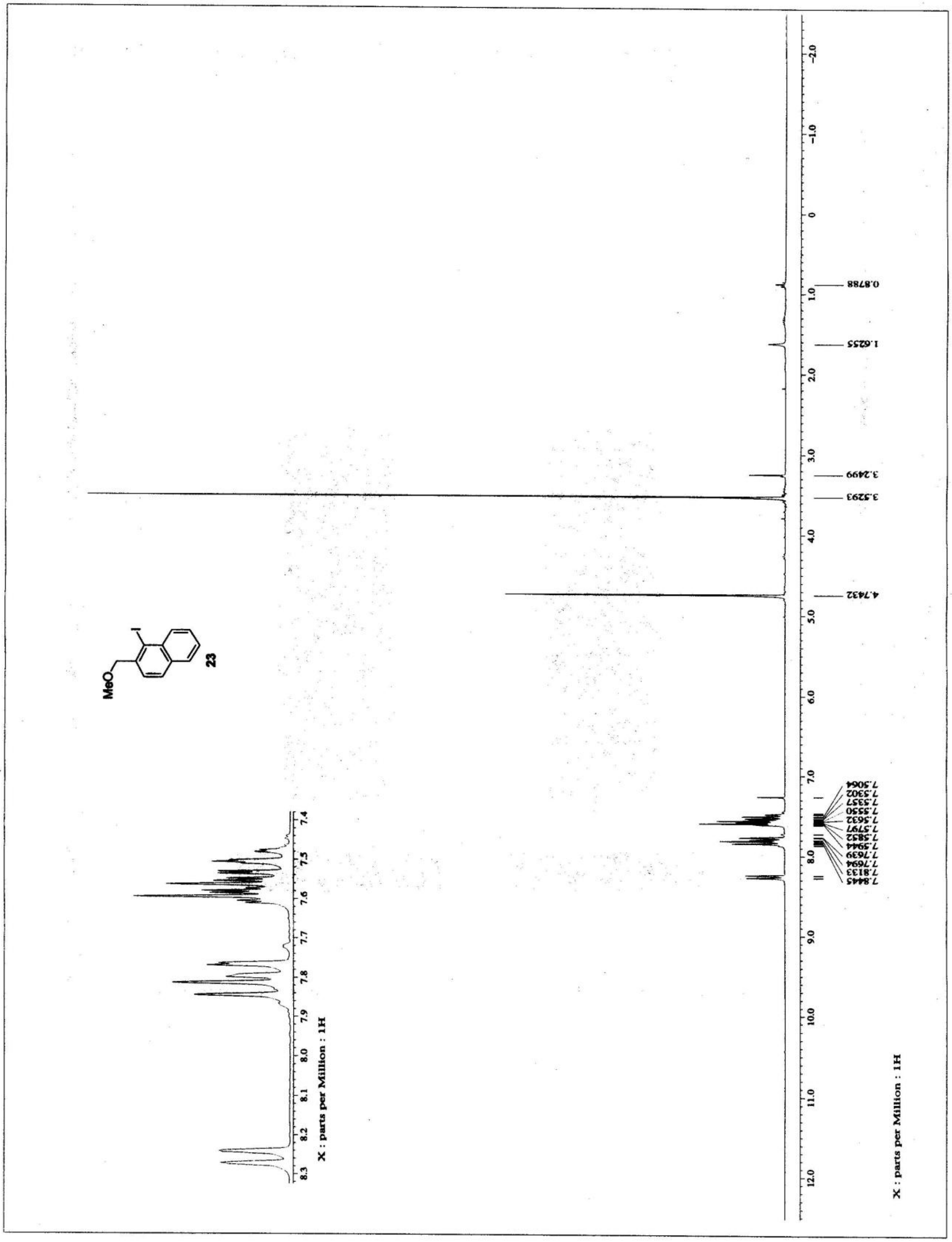
MeO
I
23
0.8788
1.6255
3.2499
3.5293
4.7432
7.3064
7.3302
7.3357
7.5550
7.5632
7.5797
7.5852
7.5944
7.7639
7.7694
7.8133
7.8445
X : parts per Million : 1H
X : parts per Million : 1H

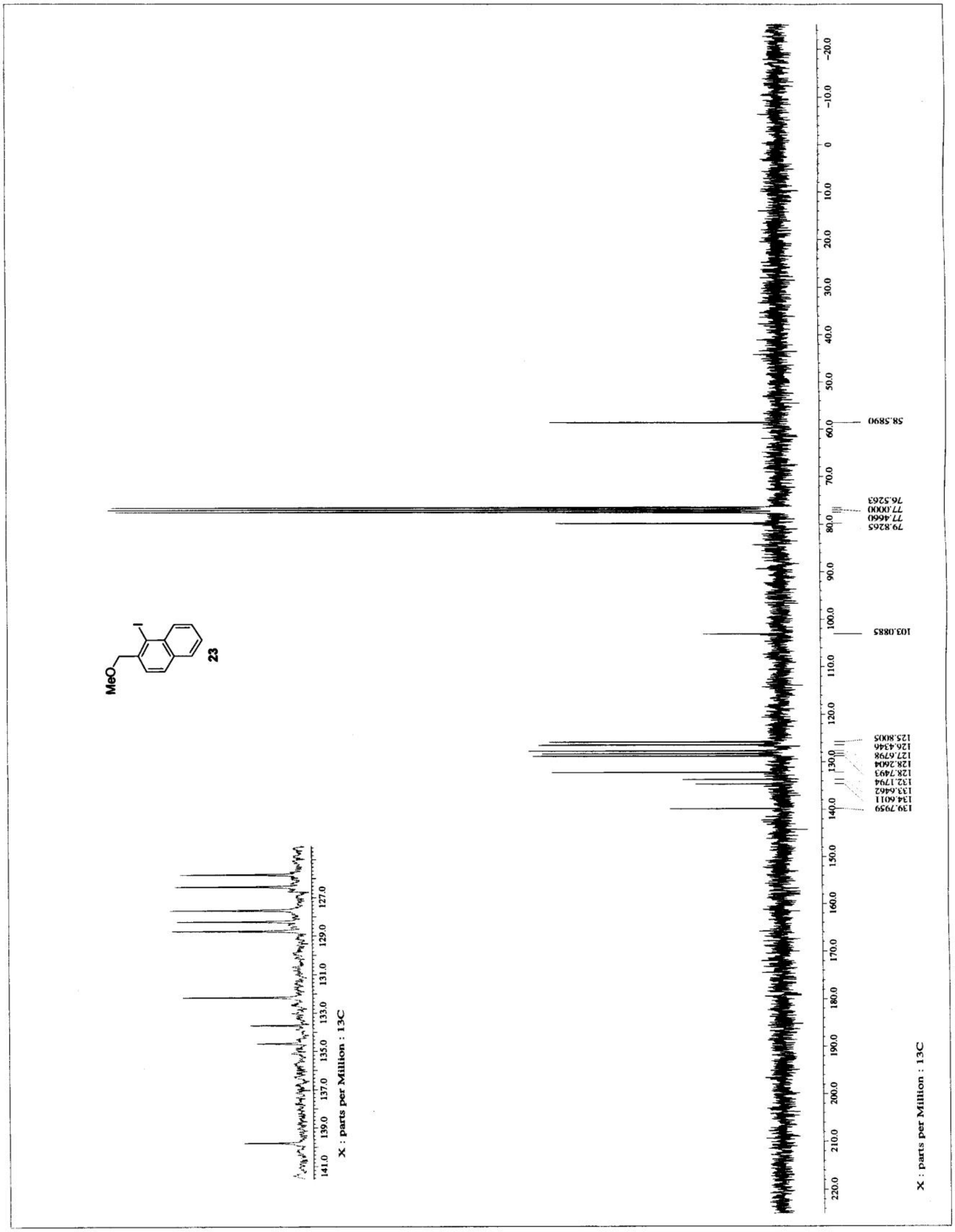
MeO
I
23
58.5890
76.5263
77.0000
77.4660
79.8265
103.0885
125.8005
126.4346
127.6798
128.2604
128.7493
132.1794
133.6462
134.6011
139.7959
X : parts per Million : 13C
X : parts per Million : 13C

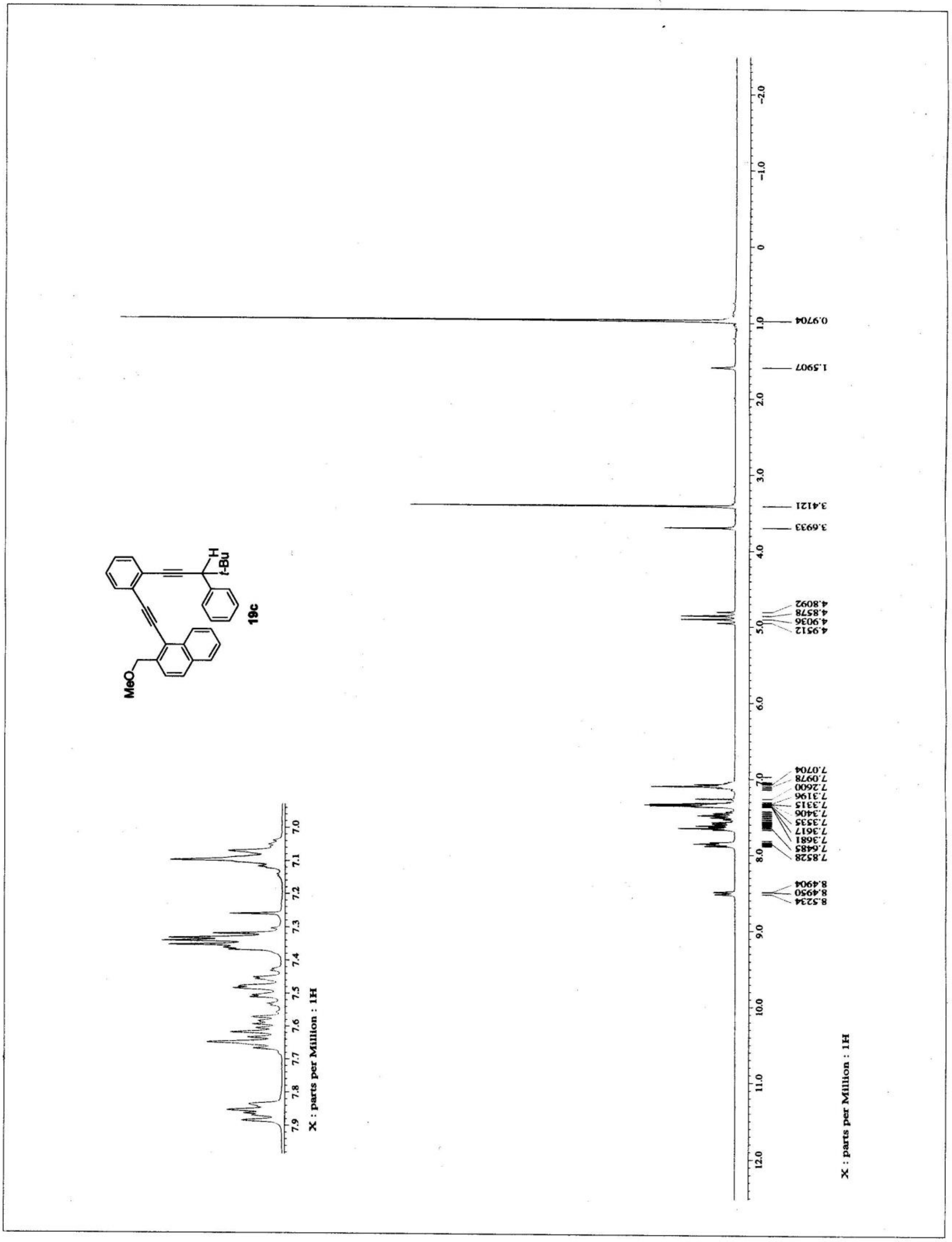
MeO
H
t-Bu
19c
0.9704
1.5907
3.4121
3.6933
4.8092
4.8578
4.9036
4.9512
7.0704
7.0978
7.2600
7.3196
7.3315
7.3406
7.3535
7.3617
7.3681
7.6485
7.8528
8.4904
8.4950
8.5234
X : parts per Million : 1H
X : parts per Million : 1H

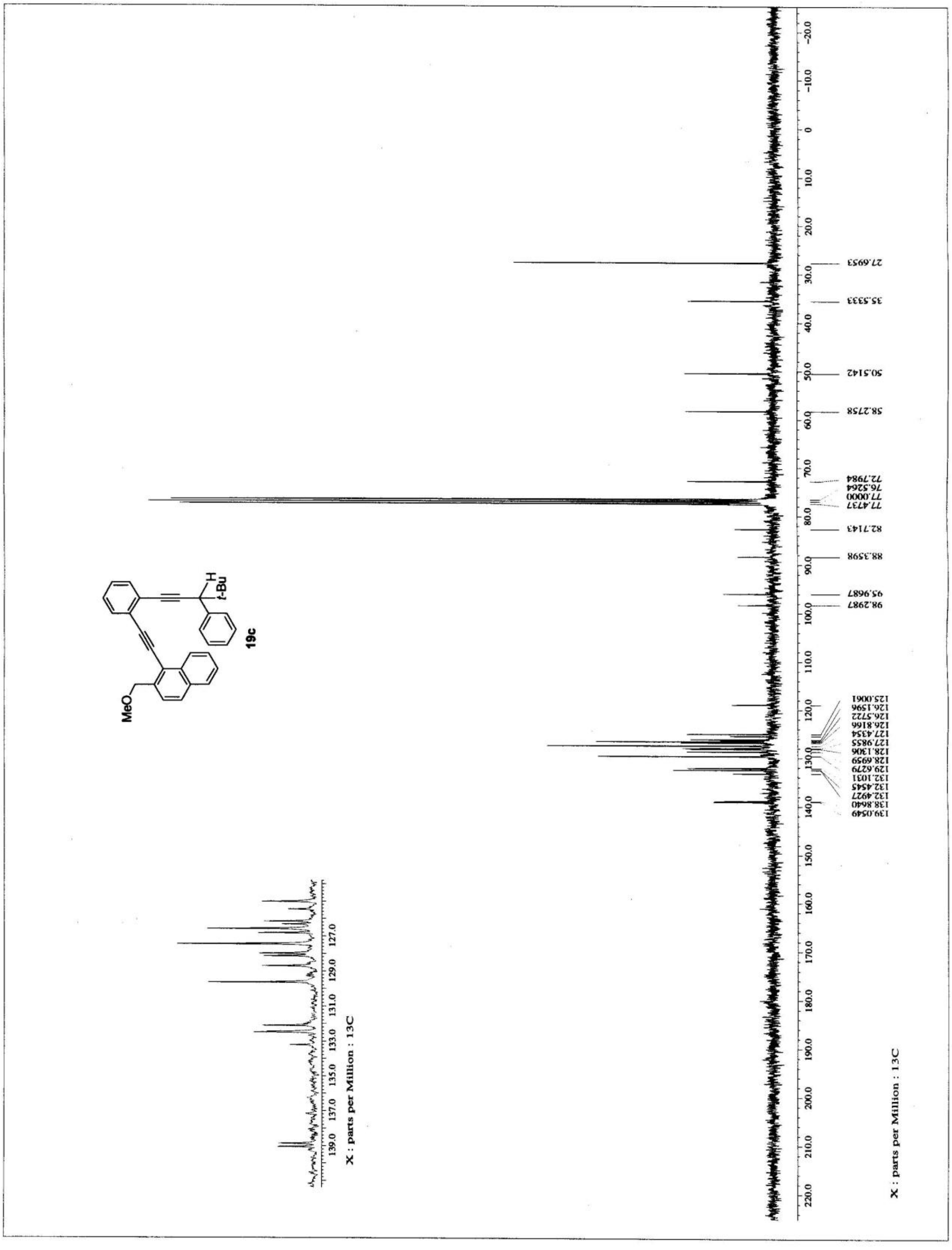
MeO
t-Bu
H
19c
27.6953
35.5333
50.5142
58.2758
72.7984
76.5264
77.0000
77.4737
82.7143
88.3598
95.9687
98.2987
125.0061
126.1596
126.5722
126.8166
127.4354
127.9855
128.1306
128.6959
129.6279
132.1031
132.4545
132.4927
138.8640
139.0549
X : parts per Million : 13C
X : parts per Million : 13C

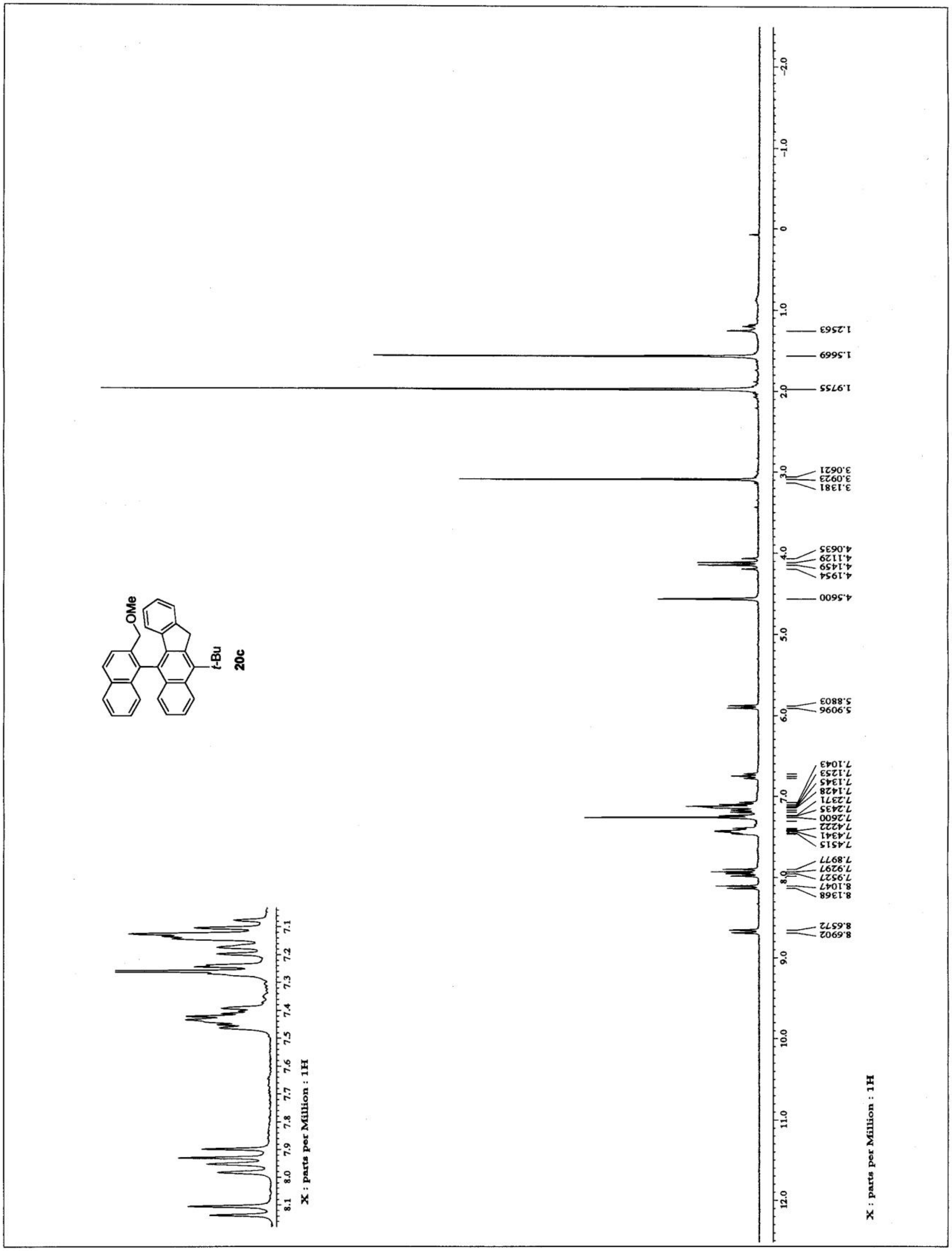

OMe
t-Bu
20c
1.2563
1.5669
1.9755
3.0621
3.0923
3.1381
4.0635
4.1129
4.1459
4.1954
4.5600
5.8803
5.9096
7.1043
7.1253
7.1345
7.1428
7.2371
7.2435
7.2600
7.4222
7.4341
7.4515
7.8977
7.9297
7.9527
8.1047
8.1368
8.6572
8.6902
X : parts per Million : 1H
X : parts per Million : 1H

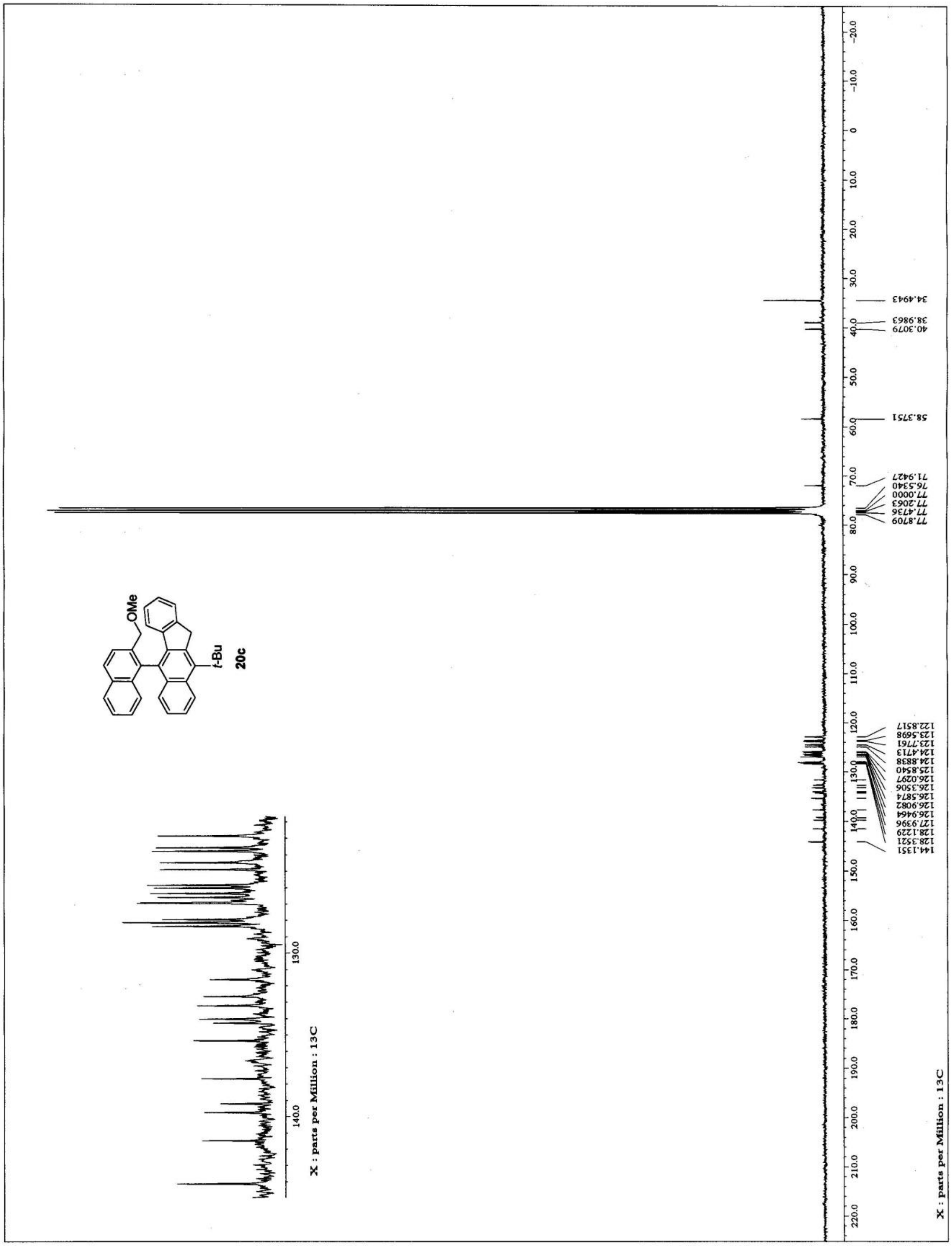
OMe
t-Bu
20c
34.4943
38.9863
40.3079
58.3751
71.9427
76.5340
77.0000
77.2063
77.4736
77.8709
122.8517
123.5698
123.7761
124.4713
124.8838
125.8540
126.0297
126.3506
126.5874
126.9082
126.9464
127.9396
128.1229
128.3521
144.1351
X : parts per Million : 13C
X : parts per Million : 13C

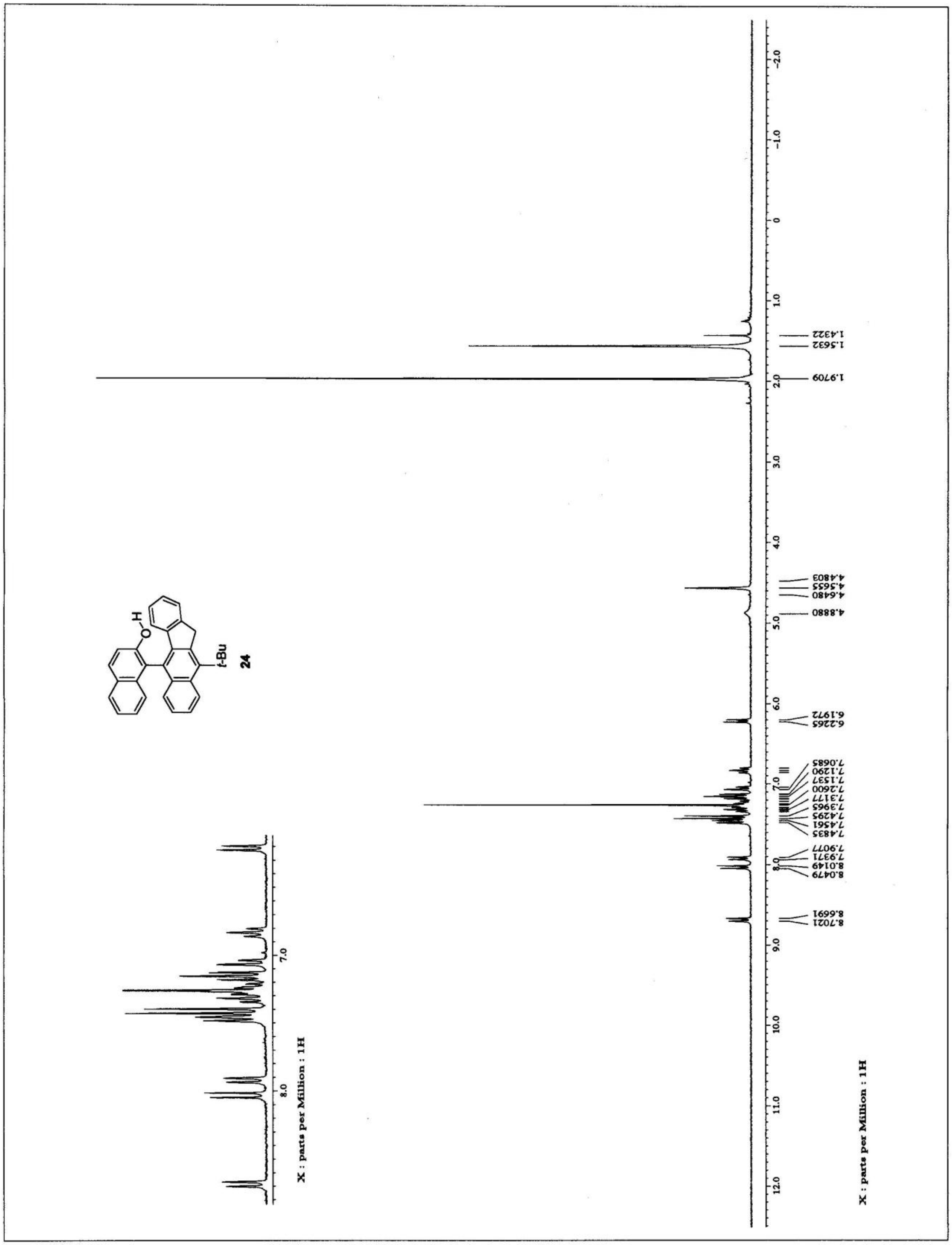

t-Bu
24
1.4322
1.5632
1.9709
4.4803
4.5655
4.6480
4.8880
6.1972
6.2265
7.0685
7.1290
7.1537
7.2600
7.3177
7.3965
7.4295
7.4561
7.4835
7.9077
7.9371
8.0149
8.0479
8.6691
8.7021
X : parts per Million : 1H
X : parts per Million : 1H

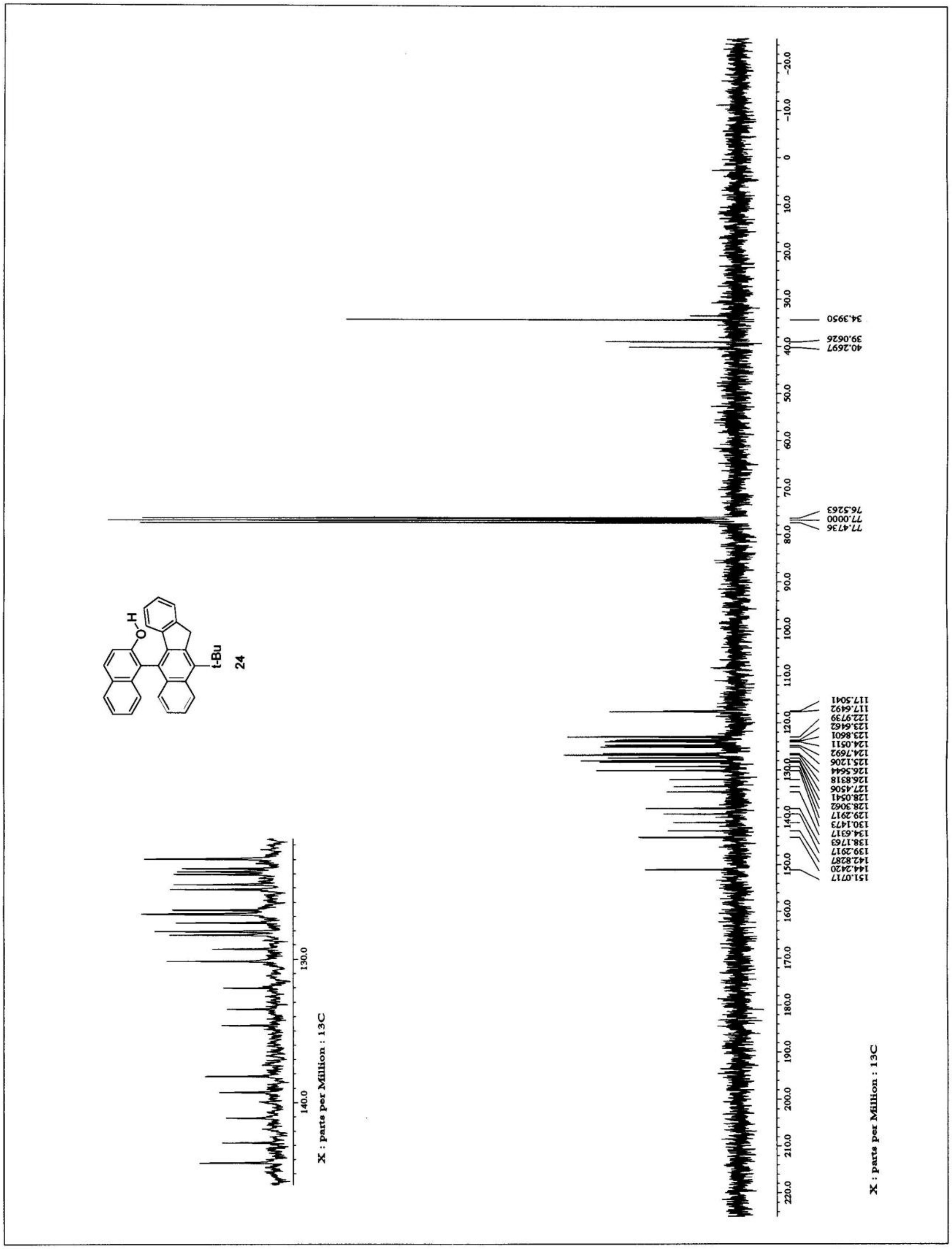

t-Bu
24
O
H
X : parts per Million : 13C
140.0
130.0
151.0717
144.2420
142.8287
139.2917
138.1763
134.6317
130.1473
129.2917
128.3062
128.0541
127.4506
126.8318
126.5644
125.1206
124.7692
124.0511
123.8601
123.6462
122.9739
117.6492
117.5041
77.4736
77.0000
76.5263
40.2697
39.0626
34.3950
220.0 210.0 200.0 190.0 180.0 170.0 160.0 150.0 140.0 130.0 120.0 110.0 100.0 90.0 80.0 70.0 60.0 50.0 40.0 30.0 20.0 10.0 0 −10.0 −20.0
X : parts per Million : 13C

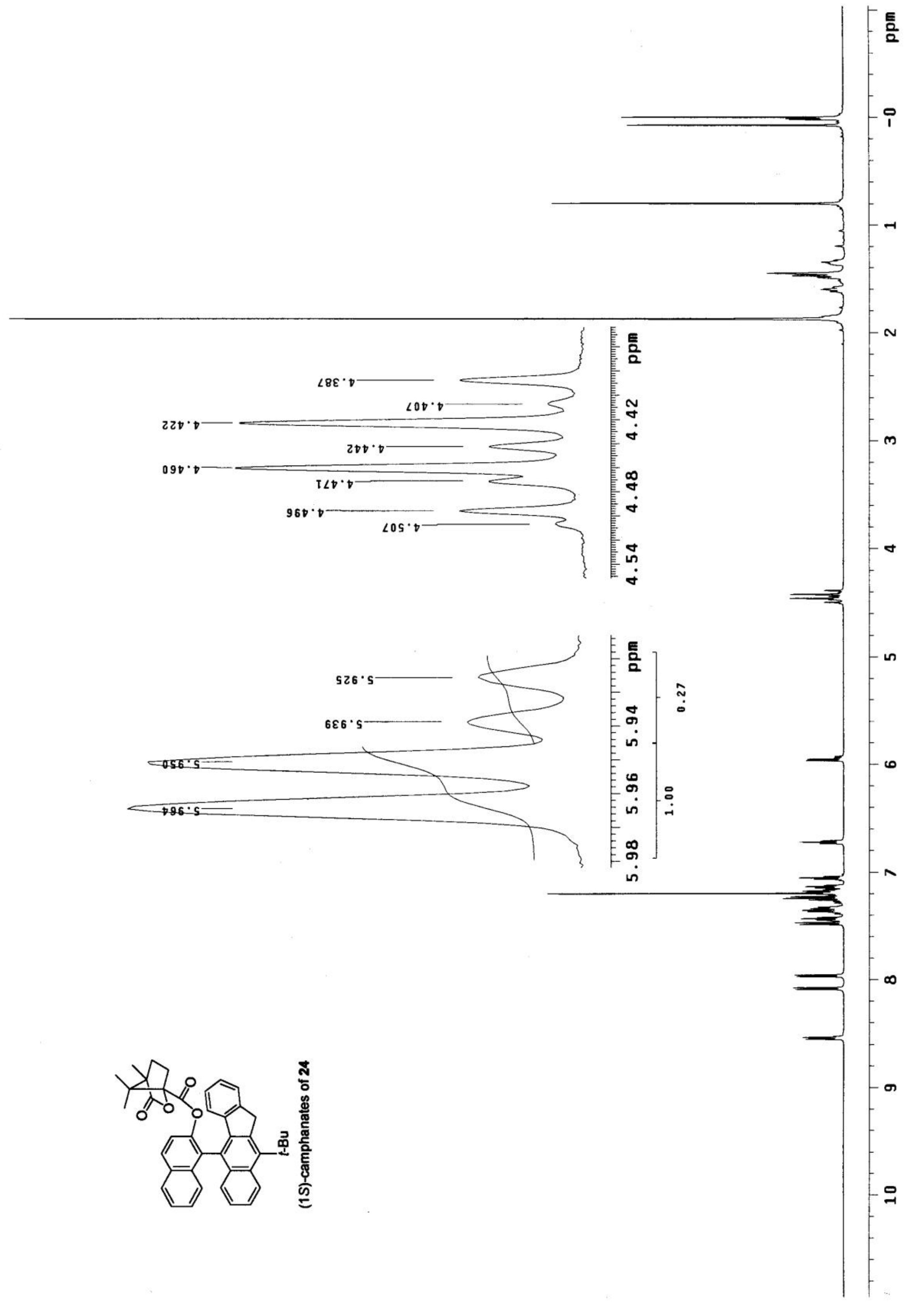
(1S)-camphanates of 24
t-Bu
ppm
4.387
4.407
4.422
4.442
4.460
4.471
4.496
4.507
4.42
4.48
4.54
5.925
5.939
5.950
5.964
5.94
5.96
5.98
0.27
1.00
